# Transcriptomic changes triggered by ouabain in rat cerebellum granule cells: Role of α3- and α1-Na^+^,K^+^-ATPase-mediated signaling

**DOI:** 10.1371/journal.pone.0222767

**Published:** 2019-09-26

**Authors:** Larisa V. Smolyaninova, Alexandra A. Shiyan, Leonid V. Kapilevich, Alexander V. Lopachev, Tatiana N. Fedorova, Tatiana S. Klementieva, Aleksey A. Moskovtsev, Aslan A. Kubatiev, Sergei N. Orlov

**Affiliations:** 1 Department of Biomembranes, Faculty of Biology, M. V. Lomonosov Moscow State University, Moscow, Russia; 2 Department of Sports Tourism Sports Physiology and Medicine, National Research Tomsk State University, Tomsk, Russia; 3 Laboratory of Clinical and Experimental Neurochemistry, Research Center of Neurology, Moscow, Russia; 4 Department of Molecular and Cell Pathophysiology, Institute of General Pathology and Pathophysiology, Moscow, Russia; 5 Central Research Laboratory, Siberian Medical State University, Tomsk, Russia; Universidade Federal do Rio de Janeiro, BRAZIL

## Abstract

It was shown previously that inhibition of the ubiquitous α1 isoform of Na^+^,K^+^-ATPase by ouabain sharply affects gene expression profile via elevation of intracellular [Na^+^]_i_/[K^+^]_i_ ratio. Unlike other cells, neurons are abundant in the α3 isoform of Na^+^,K^+^-ATPase, whose affinity in rodents to ouabain is 10^4^-fold higher compared to the α1 isoform. With these sharp differences in mind, we compared transcriptomic changes in rat cerebellum granule cells triggered by inhibition of α1- and α3-Na^+^,K^+^-ATPase isoforms. Inhibition of α1- and α3-Na^+^,K^+^-ATPase isoforms by 1 mM ouabain resulted in dissipation of transmembrane Na^+^ and K^+^ gradients and differential expression of 994 transcripts, whereas selective inhibition of α3-Na^+^,K^+^-ATPase isoform by 100 nM ouabain affected expression of 144 transcripts without any impact on the [Na^+^]_i_/[K^+^]_i_ ratio. The list of genes whose expression was affected by 1 mM ouabain by more than 2-fold was abundant in intermediates of intracellular signaling and transcription regulators, including augmented content of *Npas4*, *Fos*, *Junb*, *Atf3*, and *Klf4* mRNAs, whose upregulated expression was demonstrated in neurons subjected to electrical and glutamatergic stimulation. The role [Na^+^]_i_/[K^+^]_i_-mediated signaling in transcriptomic changes involved in memory formation and storage should be examined further.

## Introduction

Na^+^,K^+^-ATPase is an integral plasma membrane protein consisting of α-, β- and γ-subunits. It has been shown that ATP hydrolysis leads to phosphorylation of residue Asp369 in the α-subunit, providing E_1_–E_2_ conformational transition and electrogenic ion transport (3 Na^+^ versus 2 K^+^) at a baseline rate of 60–80 phosphorylation-dephosphorylation cycles per second. In addition to the ubiquitous α1-subunit, three other α-subunits are expressed in a tissue-specific manner. The α2-subunit is mainly present in skeletal muscle, heart, and brain, the α3-subunit predominates in nervous tissue, and the α4-subunit was found in testis [1–4]. In nervous tissue, the α1-subunit is expressed in both neurons and glial cells, the α2-subunit is primarily found in astrocytes and oligodendrocytes, whereas neurons are abundant in the α3-subunit [5]. Three β isoforms (β1, β2, β3) have been characterized in mammalian cells [6]. The β isoforms also exhibit a tissue-specific pattern of expression. The β1-subunit is found in nearly every tissue, the β2 isoform is found in skeletal muscle, pineal gland, and nervous tissue, and β3 is present in lung, retina, liver, and testis [7]. The β-subunits are highly glycosylated and are obligatory for delivery, conformational stability, and enzymatic activity. The γ-subunit from the family of FXYD proteins (that share a Pro-Phe-X-Tyr-Asp motif) is involved in regulation of Na^+^/K^+^-ATPase by interacting with the α- and β-subunits. The expression of FXYD proteins is tissue-specific, for example, FXYD1, mainly expressed in heart and skeletal muscle, FXYD2 (γ-subunit) and FXYD4 expressed in renal tubule, FXYD3 expressed in stomach, colon, and numerous tumors, and FXYD7 is exclusively expressed in the brain in both neurons and glial cells. It has been shown that FXYD proteins affect the apparent affinity of Na^+^/K^+^-ATPase for extracellular K^+^ [8–11] and intracellular Na^+^ [12–14].

Cardiotonic steroids (CTSs) are known as compounds sharing a common structure formed by a steroid nucleus with a lactone ring at C-17 and a hydroxyl group at C-14. Lactone rings with 5 or 6 members are the most significant feature of cardenolides and bufadienolides, which are isolated from plants and amphibians, respectively. The mechanism of inhibition of Na^+^,K^+^-ATPase by CTSs has been explored mainly with ouabain extracted from *Strophanthus gratus*. As predicted, exposure to CTSs affects numerous cellular functions related to maintenance of the transmembrane gradient of Na^+^ and K^+^, such as electrical membrane potential (E_m_), cell volume, transepithelial movement of salt and osmotically obliged water, Na^+^/H^+^ and Na^+^(K^+^)/Ca^2+^ exchange, symport of Na^+^ with inorganic phosphate, glucose, amino acids, and nucleotides, etc. During the last two decades, it has been shown that along with the above-mentioned canonical [Na^+^]_i_,[K^+^]_i_-, E_m_-, and cell volume-mediated cellular responses, CTSs can affect gene expression, membrane trafficking, and cell adhesion, proliferation, and death. The data on relative impacts of [Na^+^]_i_,[K^+^]_i_-mediated and -independent signaling in these noncanonical cellular responses triggered by CTSs are controversial (for reviews, see [15–19]).

Unlike most other cells, pyramidal and dentate neurons of the cerebral cortex and hippocampus [20–21] as well as rat granule cell culture [22, 23] are abundant with the α3-Na^+^,K^+^-ATPase isoform. Using HeLa cells transfected with cDNAs encoding distinct catalytic subunits, Jewell and Lingrel found that apparent affinity for Na^+^ of the α1 and α2 isoforms is 2-3-fold higher compared to the α3 isoform [24]. In α1-α3-transfected, monensin-treated HeLa cells the apparent K_0.5_ values of ouabain-sensitive ^86^Rb influx for cytoplasmic Na^+^ activation were ~18, 20, and 64 mM for the α1, α2, and α3 isoforms, respectively [25]. The attenuated affinity of the α3 isoform for [Na^+^]_i_ was also confirmed in a study of the kinetics of Na^+^ efflux from α1-α3-transfected HeLa cells loaded with Na^+^-sensitive fluorescent dye SBFI [26]. Keeping these differences in mind, it has been proposed that α1-Na^+^,K^+^-ATPase isoform plays a key role in the maintenance of low [Na^+^]_i_/[K^+^]_i_ ratio under baseline conditions, whereas the α3- Na^+^,K^+^-ATPase isoform is involved in normalization of the gain of [Na^+^]_i_ evoked by sustained neuronal excitation [26–28].

Numerous research teams have reported that in rodents the affinity of α3 isoform of Na^+^,K^+^-ATPase for CTSs is three orders of magnitude higher than for the α1 isoform. Thus, in transfected NIH 3T3 fibroblasts, the apparent affinity constant of rat α3-, α2-, and α1-Na^+^,K^+^-ATPase for ouabain calculated from [^3^H]-ouabain binding and ATPase assay yielded values of ~2, 115, and 48,000 nM, respectively [29]. In BALB/c 3T3 cells transfected with rat α3 and α1-Na^+^,K^+^-ATPase, K_i_ values for ouabain estimated by inhibition of ^86^Rb influx were 8 x 10^−8^ and 4.5 x 10^−5^ M, respectively [30]. These differences were confirmed by comparative analysis of the dose-dependent action of ouabain on Na^+^,K^+^-ATPase activity in adult and newborn rat brain [31] and in cultured and separated neuronal cells from rat cerebellum [32].

In the present study, we used the sharp differences in the affinity of rat α1 and α3-isoforms of Na^+^,K^+^-ATPase for CTSs to examine the relative impact of α1 and α3-mediated excitation–transcription coupling by comparing the action of low and high doses of ouabain on the intracellular Na^+^ and K^+^ content and gene expression profile in primary culture of rat cerebellar granule cells.

## Materials and methods

### Chemicals

Ouabain octahydrate (Cat #O3125) was purchased from Sigma Aldrich (USA) and was diluted to 10 mM in ultra-pure water and further used for cell treatment. Trypsin-EDTA, fetal bovine serum, penicillin-streptomycin, Hank’s Balanced Salt Solution, and trypan blue were purchased from Paneco (Russia). Neurobasal Medium, Supplement B-27, and 0.5 mM GlutaMax were purchased from Gibco (USA). MMLV RT Kit and TaqMan qPCR mix-HS were purchased from Evrogen (Russia). Primers were purchased from DNA-synthesis (Russia). GeneChip Rat Gene 2.0 ST Array; GeneChip WT PLUS Reagent Kit; GeneChipH WT Terminal Labeling Kit; and GeneChip Hybridization, Wash, and Stain Kit were purchased from Affymetrix (USA). Poly-L-ornithine, RIPA buffer, MgCl_2_, trichloroacetic acid, KCl, reagents for SDS-PAGE, cocktails of protease and phosphatase inhibitors, 3-(4,5-dimethylthiazol-2-yl)-2,5-diphenyltetrazolium bromide (MTT), and dimethyl sulfoxide were purchased from Sigma Aldrich. DC Protein Assay Kit was purchased from Bio-Rad (USA). Antibodies phospho-CREB (Ser133) (87G3) Rabbit mAb; β-Actin (D6A8) Rabbit mAb; Bcl-2 Rabbit pAb; Bax Rabbit pAb; and anti-rabbit IgG-HRP were purchased from Cell Signaling Technology (USA). SuperSignal West Pico Chemiluminescent Substrate, SuperSignal West Femto Chemiluminescent Substrate, DNase I, and PureLink RNA Mini Kit, SYTO13 were purchased from Thermo Fisher Scientific (USA).

### Primary culture of cerebellar granule cells

Commercially available Wistar rats were purchased from Pushchino Animal Incubator (Russia) and kept under standard conditions with natural light regime and freely available water and food. Decapitation was used for euthanasia and all efforts were made to minimize suffering. The animal experiments were approved by the Bioethics Committee of the Faculty of Biology, Lomonosov Moscow State University (#82-O). Cerebellum was isolated from 7- to 8-day-old rat pups under sterile conditions as described previously [33]. Cerebellum cells were dissociated with 0.25% Trypsin-EDTA (Paneco) for 15 min at 37°C. The trypsin was inactivated by incubation with 10% fetal bovine serum (Paneco). Cells were cultivated in Neurobasal-A Medium (Gibco) containing 2% Supplement B-27 (Gibco), 0.5 mM GlutaMax (Gibco), 100 U/ml penicillin-streptomycin (Paneco), and 20 mM KCl plated at density 1.2·10^5^ cells/cm^2^ onto 6-well plates pretreated with poly-L-ornithine (Sigma). The cells were maintained at 37°C, 90% humidity, and 5% CO_2_ for 7 or 8 days, at which time they had reached an advanced degree of morphological differentiation [34]. Cytosine arabinoside was added at final concentration 10 μM after 24 h in culture for inhibition of astrocyte proliferation.

### Cell death assay

Cells were seeded onto 96-well plates at density 4·10^4^ cells/cm^2^. After 3 h of ouabain treatment, cells were washed twice in Hank’s Balanced Salt Solution (Paneco). Dead cells were stained with 0.5% trypan blue (Paneco) for 5 min, the live cells were stained for 2 min with 2 μm DNA stain SYTO13 and then cells were washed in Hank’s Balanced Salt Solution. For each experimental point images of fields of view at 200x magnification were obtained in the bright field and upon excitation of fluorescence light with a wavelength of 488 nm (emission 509 nm) for SYTO 13 using Eclipse TS100 fluorescent microscope system (Nikon, Japan). The number of living neurons in culture was assessed as total number of neurons whose nuclei were stained by SYTO 13 but cells were not has been stained with trypan blue.

### MTT assay

Cells were seeded onto 96-well plates at density 4·10^4^ cells/cm^2^. After 3 h of ouabain treatment, the medium was aspirated and replaced by fresh 0.5 mg/ml MTT solution. After 3 h, the MTT solution was removed and the resulting formazan crystals were solubilized by adding 100 μl dimethyl sulfoxide to each well. After 10 min of vigorous vortexing, the absorbance in each well was read using a Synergy H4 microplate reader (Biotek, USA) at 570 nm and 660 nm. Absorbance values at 660 nm were subtracted from absorbance values at 570 nm to correct for nonspecific background.

### Quantification of intracellular Na^+^ and K^+^ content by Inductively Coupled Plasma Mass Spectrometry (ICP-MS)

Cells were seeded onto 6-well plates at density 1.2·10^5^ cells/cm^2^. After 3 h of ouabain treatment, the medium was discarded and the cells were washed twice with cold 0.1 M MgCl_2_ and lysed in 200 μl of trichloroacetic acid. The lysates were transferred to acid-washed and metal-free 0.5-ml clear microtubes and incubated for 12 h at 4°C. After the incubation, the samples were brought to 500 μl with doubly deionized water. The samples were assayed on a Varian 820-MS (USA). Concentrations of the selected elements (mg/liter) were measured by an external calibration method. The data were analyzed using ICP-MS Expert version 2.1 b-107 (USA). Na^+^ and K^+^ contents are expressed as part per million (ppm) per sample.

### RNA isolation, reverse transcription, and qPCR

Total RNA was extracted from cells grown in 6-well plates using a PureLink RNA Mini Kit (Ambion) followed by DNase I treatment (Thermo Fisher Scientific) according to the manufacturers’ protocols. One microgram of each RNA sample was reverse transcribed using random decamer primers with an MMLV RT Kit (Evrogen) following the manufacturer’s instructions. The obtained cDNA was used for qRT-PCR, the reaction being carried out with a CXF96 Real-Time PCR System (Bio-Rad Laboratories, USA) and TaqMan qPCR mix-HS (Evrogen). The primers sequences for c-Jun, c-Fos, Bcl-2, Bax, and GAPDH were: *c-fos*-forward (5’-GACAGCCTTTCCTACTACCAT-3’), *c-fos*-reverse (5’-GCTGGTGGAGATGGCTGTCA-3’), *c-fos* (5’-FAM-CTGTCAACACACAGGACTTTTGCGC-BHQ1-3’); *c-jun*-forward (5’-AAGCTCACAAGTCCCGGCAC-3’), *c-jun*-reverse (5’-CCTGTGCGAGCTGGTATGAG-3’), *c-jun* (5’-FAM-GTTCGCTCCGGGCCACTTGTTCC-BHQ1-3’); *bcl-2*-forward (5’-CACGGTGGTGGAGGAACTCT-3’), *bcl-2*-reverse (5’-CACATGACCCCACCGAACTC-3’), *bcl-2* (5’-FAM -CCACAATCCTCCCCCAGTTCACCC- BHQ1-3’); *bax*-forward (5’-GGAGACACCTGAGCTGACCT-3’), *bax*-reverse (5’-ATCGCCAATTCGCCTGAGACA-3’), *bax* (5’-FAM-CTTCTTGGTGGATGCGTCCTGGGG- BHQ1-3’); *GAPDH*-forward (5’- ACCCACGGCAAGTTCAACGG-3’), *GAPDH*-reverse (5’-CCCTTCAGGTGAGCCCCAG-3’), *GAPDH* (5’- FAM-CGGGATCTCGCTCCTGGAAGATG-BHQ1-3’). The expression of GAPDH mRNA as an internal control was used to normalize and compare the expression value of each gene of interest using the 2^–ΔΔCt^ method. The p-values were calculated using one-way ANOVA with repeated measures and Tukey’s multiple comparison test of all data to the control. Significance was accepted at p<0.05.

### Microarray analysis

RNA samples that had more than 7.0 RNA integrity number (RIN) and no detectable genomic DNA contamination were used for the subsequent gene microarray analyses. RNA quality was assessed using a 2100 Bioanalyzer (Agilent Technologies, USA). RNA samples were hybridized to a GeneChip Rat Gene 2.0 ST Array gene expression array (Affymetrix). This array detects total RefSeq 28407 transcripts. Five hundred nanograms of total RNA for each sample was processed with GeneChip WT PLUS Reagent Kit (Affymetrix). This kit uses a reverse transcription priming method that specifically primes non-ribosomal RNAs, including both poly(A) and non-poly(A) mRNAs, and generates amplified and biotinylated sense-stranded DNA as the final product. The single-stranded cDNA (5.5 mg) was fragmented and labeled using an Affymetrix GeneChipH WT Terminal Labeling Kit (Affymetrix), and 3.5 mg of the resulting cDNA was hybridized on the chip using a GeneChip Hybridization, Wash, and Stain Kit (Affymetrix). Scans of microarrays were converted into CEL files by the scanner software and then jointly preprocessed in the Affymetrix Expression Console (build 1.4.1.46). The expression levels were statistically analyzed using the Affymetrix Transcriptome Analysis Console (version 3.0). The data were initially normalized by the Robust Multichip Average (RMA)-Sketch algorithm, which uses background adjustment, quantile normalization, and summarization. Then, the normalized data were analyzed by principal component analysis (PCA) [35] to identify patterns in the dataset and to highlight similarities and differences among the samples. Major sources of variability identified within the dataset by PCA were used as grouping variabilities for analysis of variance (ANOVA) with n = 3 for each group of samples. The ensuing data were filtered to identify transcripts with statistically significant variation of expression among the groups that are modulated by at least 20%, with multiple testing correction by the false discovery rate (FDR). For interpreting genome-wide expression profiles, Gene Set Enrichment Analysis (GSEA) was applied.

### SDS-PAGE and Western blotting

Cells were seeded onto 6-well plates at density 1.2·10^5^ cells/cm^2^. After 3 h of ouabain treatment, the medium was discarded, and the cells were washed with cold Hank’s Balanced Salt Solution and lysed in RIPA buffer (Sigma) containing cocktails of protease and phosphatase inhibitors (Sigma). The total protein concentration was measured using a DC Protein Assay Kit (Bio-Rad) and 15 μg of protein from cell lysates per sample was separated by 15% SDS-PAGE and transferred to PVDF membrane. Biotinylated proteins served as molecular weight markers (Cell Signaling Technologies, Cat#7727). The membranes were blocked and incubated overnight with primary and appropriate secondary HRP-conjugated antibodies: phospho-CREB (Ser133) (87G3) Rabbit mAb (Cell Signaling Technologies, Cat#9198) at dilution 1:1000; β-Actin (D6A8) Rabbit mAb (Cell Signaling Technology, Cat#8457) at dilution 1:1000; Bcl-2 Rabbit pAb (Cell Signaling Technology, Cat#2876) at dilution 1:1000; Bax Rabbit pAb (Cell Signaling Technology, Cat#2772) at dilution 1:1000; anti-rabbit IgG-HRP (Cell Signaling Technology, Cat#7074) at dilution 1:3000; and anti-biotin, HRP-linked Antibody (Cell Signaling Technology, Cat#7075) at dilution 1:3000. The desired protein bands were visualized using SuperSignal West Pico Chemiluminescent Substrate and SuperSignal West Femto Chemiluminescent Substrate (Thermo Fisher Scientific) according to the manufacturer’s protocol. Chemiluminescence was detected using the ChemiDoc XRS+ System (Bio-Rad, USA) and intensity counted using Image Lab 3.0 software (Bio-Rad, USA). In each experiment the control value was taken for 100%. β-Actin was used as loading control to ensure that the total amount of protein did not change.

### Statistics

The data were analyzed using GraphPad Prism4 Software and by applying one-way ANOVA with repeated measures and Dunnett’s comparison of all data to the control. Significance was accepted at p<0.05.

## Results and discussion

**Fig 1:**
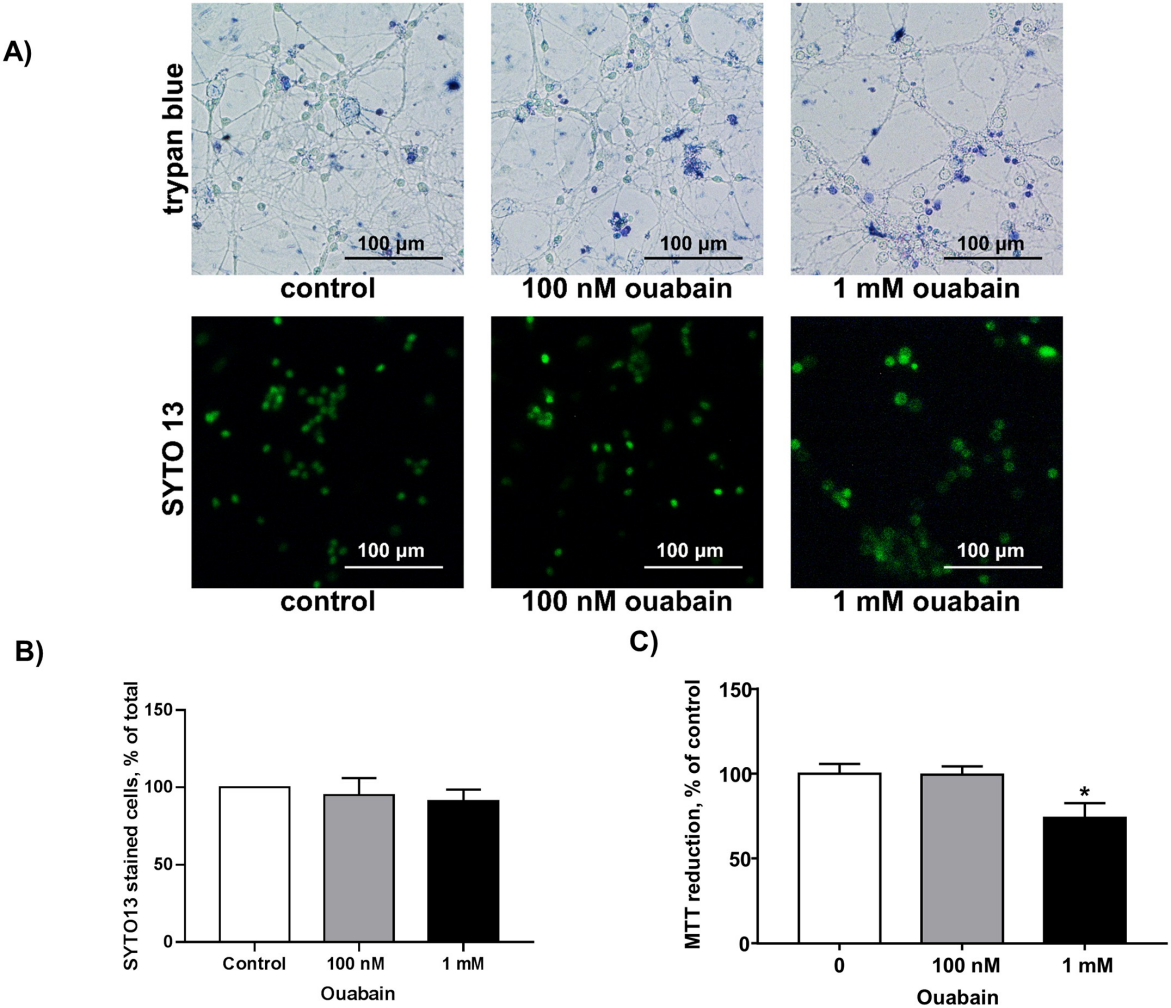
Estimation of viability of rat cerebellum granule cells by trypan blue staining and SYTO13 (A,B) and MTT reduction assay (C). **A)** Representative micrographs of control cells and cells exposed to 100 nM and 1 mM ouabain for 3 h. Bars = 100 μm. **B)** Percentage of SYTO 13-stained control cells and cells exposed to 100 nM and 1 mM ouabain for 3 h. **C)** MTT reduction assay of control cells and cells exposed to 100 nM and 1 mM ouabain for 3 h. Means ± SD for 4 (**B**) and 8 (**C**) independent experiments are shown. *—p<0.05.

**Fig 2:**
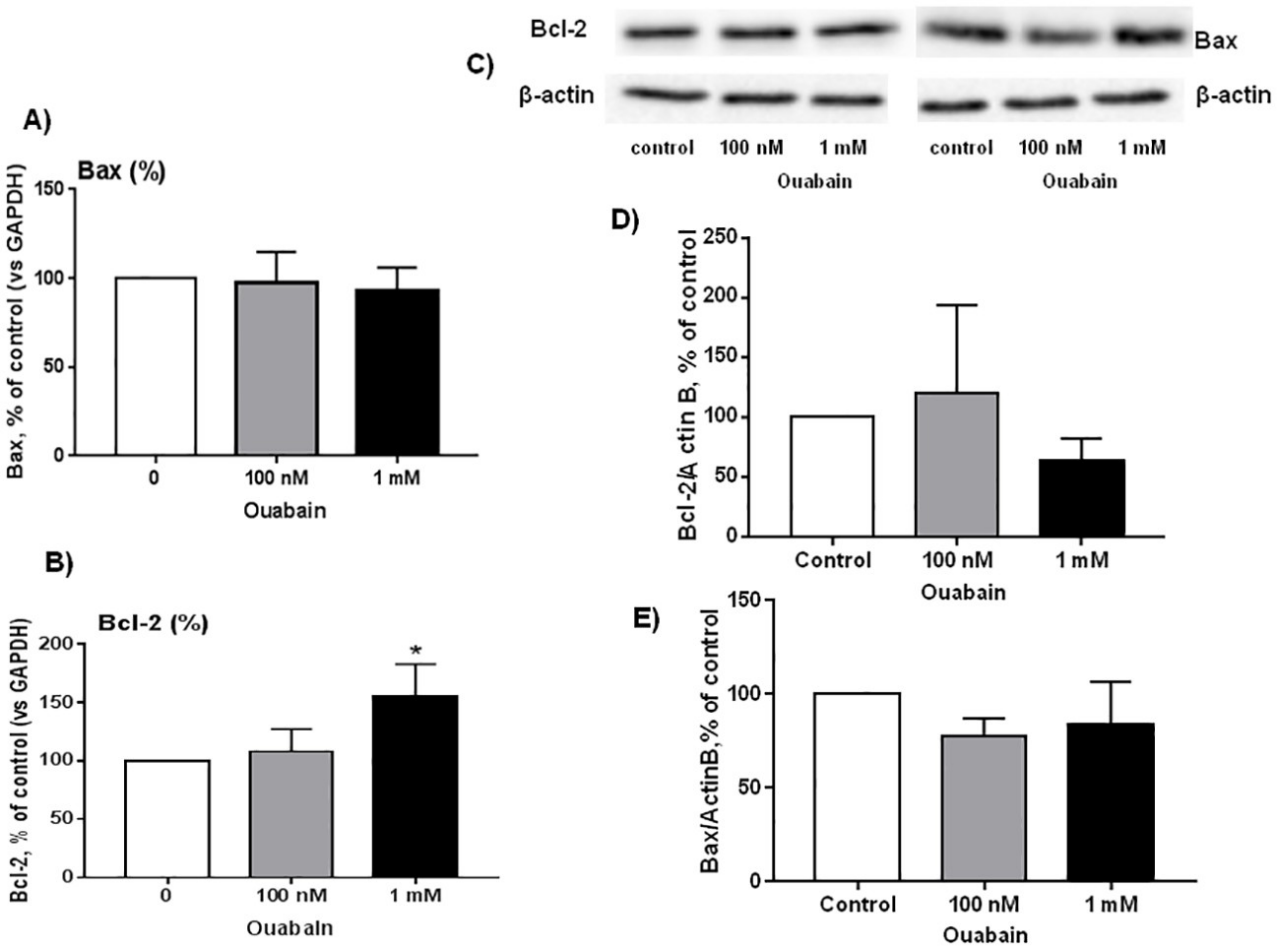
Effect of ouabain on content Bcl-2 and Bax mRNAs (A,B) and protein (C-E) in rat cerebellum granule cells. Cerebellum granule cells were incubated in the absence or presence 100 nM and 1 mM ouabain for 3 h. Bcl-2/GAPDH (**A**) and Bax/GAPDH (**B**) ratios in the absence of ouabain were taken as 100%. Representative Western blot (**C**) and Bcl-2/Actin B (**D**) and Bax/Actin B (**E**) ratio. Bcl-2/Actin B and Bax/Actin B ratios in the absence of ouabain were taken as 100%. Means ± SD for 4 independent experiments are shown. *—p<0.05.

Previously, it has been shown that long-term (24 h) exposure to 3 mM ouabain did not affect survival of rat astrocytes, vascular smooth muscle, and endothelial cells, in contrast with the cytotoxic action of ouabain on the human cells. Importantly, transfection of human endothelial cells with rat α1-Na^+^,K^+^-ATPase protected them from the cytotoxic action of high doses of ouabain (3–3000 μM) [36]. Consistent with a previous report [37], we observed that 3 h exposure to ouabain did not affect survival of rat cerebellum granule cells (Fig 1A). Thus, the number of trypan blue-stained cells was less than 3–5% in control conditions as well as in the presence of 100 nM or 1 mM ouabain. We also did not observe any significant action of ouabain on the number of neurons stained with SYTO13 (Fig 1B). In additional experiments, we estimated cell viability by MTT assay. We found that 3 h exposure of rat cerebellum granule cells to 1 mM ouabain decreased MTT reduction by 25% (Fig 1C). It should be noted, however, that exposure of rat aortic endothelial cells and primary astrocytes to 5 mM ouabain led to rapid decline of MTT reduction via attenuation of intracellular accumulation of MTT, without any impact on cell survival estimated by chromatin cleavage, caspase-3, and attachment assays [38]. Keeping these data in mind, we quantified the transcription and expression level of pro-apoptotic Bax and anti-apoptotic Bcl-2 by qPCR and Western Blotting, respectively (Fig 2). Bcl-2 family proteins are related to the formation of channels in mitochondrial membranes and regulate cytochrome c release. The released cytochrome c from mitochondria may activate the intrinsic apoptotic pathway via apoptosome formation and caspase-9 activation and thus drive cells to apoptosis. We found that exposure to 1 mM ouabain elevated *Bcl-2* transcription by 50% (Fig 2B) without any impact on Bcl-2 protein content (Fig 2D). Neither *Bax* mRNA (Fig 2A) nor Bax protein (Fig 2E) content was affected by low and high doses of ouabain. Viewed collectively these results show that similar to other rodent cells [37] exposure to ouabain does not affected the survival of rat cerebellum granule cells.

Table 1 shows that 3 h inhibition of both α1- and α3-Na^+^,K^+^-ATPase in rat cerebellum granule cells by 1 mM ouabain increased intracellular Na^+^ content 8-fold and decreased [K^+^]_i_ by 6-fold, whereas selective inhibition of CTS-sensitive α3-Na^+^,K^+^-ATPase by 100 nM ouabain had no any significant impact on these parameters. These results suggest that under the baseline conditions, α3-Na^+^,K^+^-ATPase has negligible impact on intracellular Na^+^ and K^+^ content. This conclusion is consistent with recent data showing very modest increment of [Na^+^]_i_ in SBFI-loaded cultured rat hippocampal and striatal neurons treated with 1000 nM ouabain, which is in contrast with 7-fold elevation of [Na^+^]_i_ evoked by 1 mM ouabain [28]. In rat cerebellum granule cells, the ouabain-resistant α1 isoform accounts for about 70%, and the ouabain-sensitive α3 isoform accounts for about 30% of total Na^+^, K^+^-ATPase activity in primary rat cerebellar neurons [23]. Thus, it might be proposed that inhibition of the α3 subunit results in local perturbation of Na^+^ and K^+^ concentrations, but increased activity of the α1 subunit can compensate for these changes.

**Table 1:** Effect of ouabain on intracellular Na^+^ and K^+^ content in rat cerebellum granule cells.

	[Na^+^]_i_ content, ppm	[K^+^]_i_ content, ppm
Control cells	1.15 ± 0.39	11.5 ± 2.93
Ouabain, 100 nM	1.05 ± 0.24	11.2 ± 2.77
Ouabain, 1 mM	9.92 ± 6.49	1.97 ± 0.60

The cells were incubated in the absence or presence of 100 nM or 1 mM ouabain for 3 h. Intracellular Na^+^ and K^+^ content in the absence of ouabain was taken as 100%. Mean ± SD obtained in 3 independent experiments.

Exposure of rat cerebellum granule cells to 100 nM or 1 mM ouabain for 3 h resulted in appearance of 144 and 994 differentially expressed transcripts, with the maximal degree of activation and inhibition of 3.22 versus 18.82 and 2.91 versus 2.14, respectively (Table 2) (for complete lists of transcripts whose expression was affected by 100 nM or 1 mM ouabain, see S1 and S2 Tables). Assuming that transcriptomic changes in the presence of 100 nM or 1 mM ouabain are independent events, the number of identified common transcripts (N_COM_) might be less than N_COM_ = (N_100_ × N_1_) × N_TOT_^–1^, where N_100_ and N_1_ are numbers of differential expressed transcripts triggered by 100 nM and 1 mM ouabain (144 and 994, respectively), and N_TOT_ = 28407 is the total transcript number covered by the microarray assay. The calculated number N_COM_ = 5.04 was higher than the number of common differentially expressed transcripts identified in our study (n = 2; Table 3). This analysis strongly suggests that signaling pathways underlining transcriptomic changes triggered by low and high doses of ouabain are different. This conclusion is supported by data considered below.

**Table 2:** Number of differentially expressed transcripts in ouabain-treated rat cerebellum granule cells.

	Ouabain, 100 nM	Ouabain, 1 mM
***Upregulated***		
Number of transcripts	73	403
Maximal fold of activation	3.22	18.82
***Downregulated***		
Number of transcripts	71	591
Maximal fold of inhibition	2.14	2.91

**Table 3:** Genes whose expression was affected by both 100 nM and 1 mM ouabain.

Gene Symbol, Title	Fold of inhibition by 100 nM ouabain / p-value	Fold of inhibition by 1 mM ouabain / p-value
*Oxsm*, 3-oxoacyl-ACP synthase	–1.34 / 0.030341	–1.66 / 0.031923
*OC685989*, hypothetical protein	–1.37 / 0.036192	–1.48 / 0.039919

Transcripts whose expression was altered by more than 1.30-fold with p<0.05 were subject to analysis. All experiments were repeated 3 times.

For detailed assessment of effects of low and high doses of ouabain, we performed Affymetrix whole-transcriptome-gene-expression analysis with subsequent functional annotation of the classes of genes that are enriched among the genes differentially regulated in ouabain-treated cells using Gene Set Enrichment Analysis (GSEA). In 1 mM ouabain-treated cells, GSEA revealed at false discovery rate FDR<1%: 269 upregulated (S3 Table) and 103 downregulated (S4 Table) gene sets belonging to the sub-ontology biological process; 32 upregulated (S5 Table) and 16 downregulated (S6 Table) gene sets belonging to sub-ontology molecular function; 1 upregulated (S7 Table) and 8 downregulated (S8 Table) gene sets belonging to the sub-ontology cellular component. We summarized the annotations by removing redundant GO terms and visualized the remaining terms in semantic similarity-based graphs using REViGO [39] with the SimRel measure [40]. We present these graphs in Supporting Information (S1–S6 Figs).

In 100 nM ouabain-treated cells, GSEA revealed almost no significant processes at FDR<1%. We used NES cut-off ± 1.35 to represent the data. In 100 nM ouabain-treated cells, GSEA revealed at FDR<25%: 293 upregulated (S9 Table) and 114 downregulated (S10 Table) gene sets belonging to the sub-ontology biological process; 30 upregulated (S11 Table) and 48 downregulated (S12 Table) gene sets belonging to sub-ontology molecular function; 8 upregulated (S13 Table) and 27 downregulated (S14 Table) gene sets belonging to the sub-ontology cellular component. We represent these graphs in Supporting Information (S7–S12 Figs).

**Fig 3:**
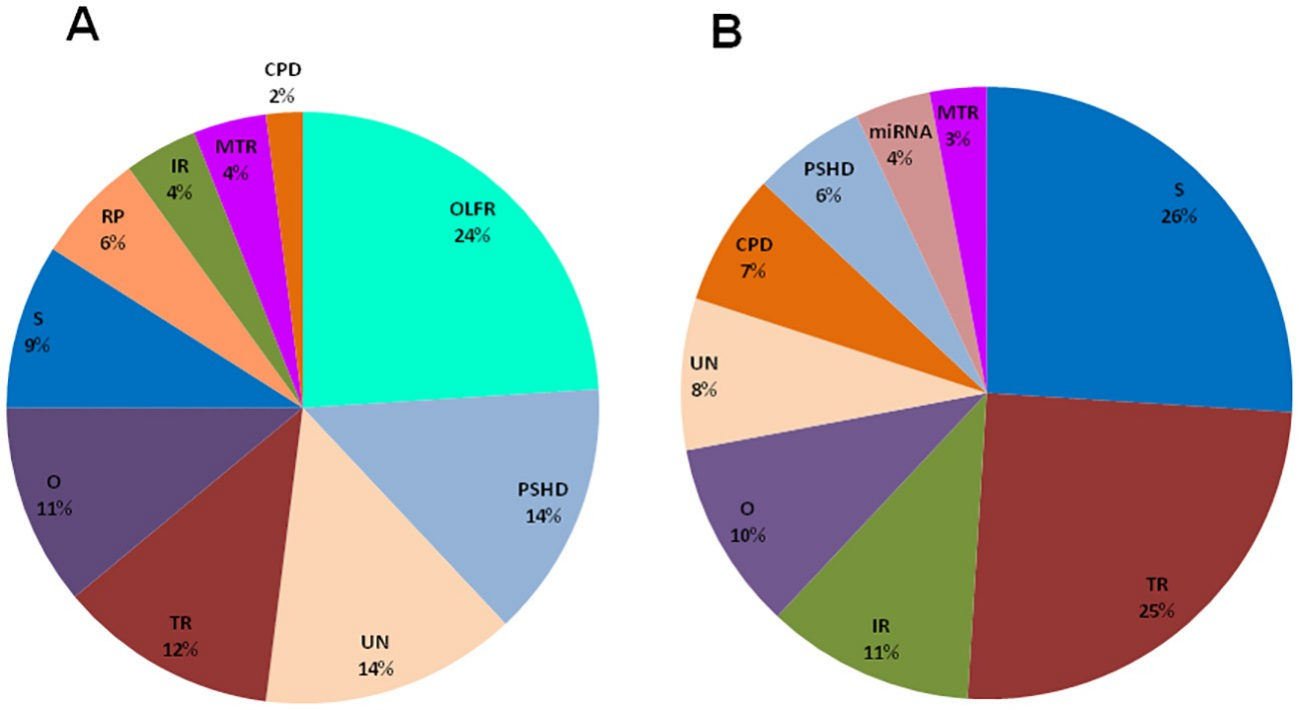
Distribution of ouabain-sensitive genes detected in cerebellum granule cells and listed in Tables 4–7 among major functional categories. Cells were incubated in the presence of 100 nM (**A**) or 1 mM ouabain (**B**) for 3 h. Functional categories: CPD—cell proliferation and death; IR—immune and inflammatory responses; miRNA—microRNAs; MTR—membrane transporters; O—others; OLFR—olfactory receptors; PSHD—protein, synthesis, holding, and degradation; RP—ribosomal proteins; S—signaling; TR—transcription regulation; UN—uncharacterized.

Keeping in mind protein multifunctionality, we limited this analysis to ten major functional categories (Fig 3, Tables 4–7). Fig 3A shows that the genes whose expression was affected by 100 nM ouabain is abundant for olfactory receptors. Olfactory receptors belong to a large gene superfamily comprising approximately 900 genes in the rat genome [41]. Importantly, the relative number of olfactory receptors among differentially expressed genes identified in neurons treated with low doses of ouabain (24%) was 8-fold higher than in the total rat genome. The function of these proteins as seven-transmembrane-type G-protein-coupled receptors is well defined in olfactory epithelium cells. In these highly specialized cells, the binding of pheromones by olfactory receptors leads to activation of adenylate cyclase, elevation of intracellular cAMP, opening of cAMP-gated, Ca^2+^-permeable channels [42, 43], which in turn, triggers diverse social responses [44]. During the last two decades, expression of olfactory receptors has also been demonstrated in skeletal muscle cells and other non-chemosensory tissues [45]. Recently, we reported that in cultured C2C12 myotubes, both ouabain and electrical pulse stimulation affect expression of olfactory receptors via Ca^2+^-mediated signaling [46]. The functional significance of altered expression of olfactory receptors in neurons triggered by low doses of ouabain remains unknown.

**Table 4:** Genes whose expression was increased by 100 nM ouabain by more than 1.3-fold.

Gene Symbol, Title	Functional category	Fold of activation / p-value
*Olr551*, olfactory receptor 551	OLFR	1.87 / 0.018489
*SPC24*, NDC80 kinetochore complex component	PSHD	1.55 / 0.04222
*Olr1334*, olfactory receptor 1334	OLFR	1.5 / 0.037193
*RGD1310495*, similar to KIAA1919 protein	MTR	1.47 / 0.044616
*Olr397*, olfactory receptor 397	OLFR	1.47 / 0.014367
*LOC102548248*, calphotin-like	UN	1.47 / 0.032125
*Csdc2*, cold shock domain containing C2, RNA binding	TR	1.45 / 0.033715
*Olr1394*, olfactory receptor 1394	OLFR	1.44 / 0.032407
*LOC102556805*, rho GTPase-activating protein 20-like	S	1.43 / 0.023919
*Drgx*, dorsal root ganglia homeobox	TR	1.41 / 0.044668
*Akap3*, A kinase (PRKA) anchor protein 3	PSHD	1.39 / 0.026649
*Map2k4*, mitogen activated protein kinase kinase 4	S	1.38 / 0.01881
*LOC498601*, similar to cyclin B2	UN	1.38 / 0.03072
*Snrpf*, small nuclear ribonucleoprotein polypeptide F	TR	1.38 / 0.003448
*Mageb16*, melanoma antigen family B, 16	IR	1.37 / 0.006286
*Pnoc*, prepronociceptin	S	1.37 / 0.016375
*LOC100361079*, ribosomal protein L36-like	RP	1.36 / 0.007825
*Emd*, emerin	PSHD	1.35 / 0.034983
*Tcp1-ps1*, t-complex protein 1, pseudogene 1	PSHD	1.35 / 0.00873
*Rpl22*, ribosomal protein L22	RP	1.35 / 0.018718
*Akr1c12*, aldo-keto reductase family 1, member C12	O	1.35 / 0.031111
*Rpl36*, ribosomal protein L36	TR	1.34 / 0.043525
*Fam174a*, family with sequence similarity 174, member A	O	1.33 / 0.028861
*Olr352*, olfactory receptor 352	OLFR	1.33 / 0.021153
*Ctla2a*, cytotoxic T lymphocyte-associated protein 2 alpha	PSHD	1.33 / 0.020505
*MGC114246*, similar to cathepsin R	PSHD	1.33 / 0.045924
*LOC102551267*, paired box protein Pax-7-like	TR	1.32 / 0.001153
*LOC296235*, similar to Cystatin S precursor (LM protein)	PSHD	1.32 / 0.010687
*Rpl34-ps1*, ribosomal protein L34, pseudogene 1	RP	1.32 / 0.049551
*Atmin*, ATM interactor	TR	1.32 / 0.044838
*RGD1559972*, similar to ribosomal protein L27a	RP	1.32 / 0.034465
*Acot13*, acyl-CoA thioesterase 13	O	1.32 / 0.047266
*Pcp4*, Purkinje cell protein 4	O	1.31 / 0.024075

The cells were incubated in the absence or presence of 100 nM ouabain for 3 h. **Functional categories: CPD**—cell proliferation and death; **IR**—immune and inflammatory responses; **miRNA**—microRNAs; **MTR**—membrane transporters; **O**—others; **OLFR**—olfactory receptors; **PSHD**—protein, synthesis, holding and degradation; **RP**—ribosomal proteins; **S**—signaling; **TR**—transcription regulation; **UN**—uncharacterized. All experiments are repeated 3 times.

**Table 5:** Genes whose expression was decreased by 100 nM ouabain by more than 1.3-fold.

Gene Symbol, Title	Functional category	Fold of inhibition / p-value
*Olr1393*, olfactory receptor 1393	OLFR	–1.31 / 0.007599
*Olr587*, olfactory receptor 587	OLFR	–1.31 / 0.035127
*Olr1410*, olfactory receptor 1410	OLFR	–1.31 / 0.02515
*Cox6c–ps1*, cytochrome c oxidase subunit VIc, pseudogene	O	–1.31 / 0.016536
*Usp49*, ubiquitin specific peptidase 49	PSHD	–1.31 / 0.036476
*Tmem266*, transmembrane protein 266	UN	–1.31 / 0.02683
*Gnb1l*, guanine nucleotide binding protein (G protein)	S	–1.31 / 0.005584
*Sntb1*, syntrophin, beta 1	PSHD	–1.32 / 0.027004
*LOC102549817*, zinc finger protein 124-like	TR	–1.33 / 0.047193
*LOC10369054*1, uncharacterized	UN	–1.33 / 0.011505
*RGD1311300*, similar to T cell receptor V delta 6	UN	–1.34 / 0.014249
*Lrrc72*, leucine rich repeat containing 72	UN	–1.34 / 0.00591
*Oxsm*, 3–oxoacyl-ACP synthase, mitochondrial	O	–1.34 / 0.030341
*Olr1695*, olfactory receptor 1695	OLFR	–1.35 / 0.034048
*Cyyr1*, cysteine / tyrosine–rich 1	UN	–1.35 / 0.037929
*Olr1374*, olfactory receptor 1374	OLFR	–1.36 / 0.031302
*Defb25*, defensin beta 25	IR	–1.36 / 0.016742
*Defb41*, defensin beta 41	IR	–1.36 / 0.042335
*Spata31e1*, SPATA31 subfamily E, member 1	CPD	–1.36 / 0.0433
*Ric8b*, RIC8 guanine nucleotide exchange factor B	S	–1.37 / 0.042087
*LOC689679*, similar to Discs large homolog	UN	–1.37 / 0.010808
*LOC685989*, hypothetical protein LOC685989	UN	–1.37 / 0.036192
*Snap25*, synaptosome–associated protein 25	MTR	–1.39 / 0.036352
*Olr1192*, olfactory receptor 1192	OLFR	–1.39 / 0.00279
*Zkscan3*, zinc finger with KRAB and SCAN domains 3	TR	–1.39 / 0.02762
*Olr1875*, olfactory receptor 1875	OLFR	–1.40 / 0.039398
*Cdkl5*, cyclin-dependent kinase-like 5	S	–1.41 / 0.008764
*Olr687*, olfactory receptor 687	OLFR	–1.42 / 0.015524
*Olr639*, olfactory receptor 639	OLFR	–1.44 / 0.03603
*Olr1065*, olfactory receptor 1065	OLFR	–1.44 / 0.033742
*RGD1566085*, similar to pyridoxal kinase	O	–1.47 / 0.044618
*Olr1246*, olfactory receptor 1246	OLFR	–1.54 / 0.015191
*Slc6a12*, solute carrier family 6 neurotransmitter transporter	MTR	–1.56 / 0.015467

The cells were incubated in the absence or presence of 100 nM ouabain for 3 h. For functional categories see Table 4. All experiments are repeated 3 times.

**Table 6:** Genes whose expression was increased by 1 mM ouabain by more than 2-fold.

Gene Symbol, Title	Functional Category	Fold of activation / p-value
*Il6*, interleukin 6	IR	18.82 / 0.017424
*Ptgs2*, prostaglandin-endoperoxide synthase 2	IR	16.59 / 0.00836
*Tfpi2*, tissue factor pathway inhibitor 2	PSHD	15.82 / 0.035903
*Npas4*, neuronal PAS domain protein 4	TR	12.97 / 0.013906
*Gem*, GTP binding protein overexpressed in skeletal muscle	S	8.45 / 0.014514
*Cyr61*, cysteine-rich, angiogenic inducer, 61	IR	8.09 / 0.005394
*Zfp36*, zinc finger protein 36	TR	7.56 / 0.032644
*Ccl7*, chemokine (C-C motif) ligand 7	IR	6.88 / 0.002458
*Has2*, hyaluronan synthase 2	O	6.84 / 0.001877
*Cxcl1*, chemokine (C-X-C motif) ligand 1	IR	5.62 / 0.014029
*Klf6*, Kruppel-like factor 6	TR	5.52 / 0.025668
*Adamts1*, ADAM metallopeptidase with thrombospondin motif	PSHD	5.08 / 0.005289
*Ptx3*, pentraxin 3, long	IR	5.02 / 0.00991
*Inhba*, inhibin beta-A	CPD	4.94 / 0.018744
*Bhlhe40*, basic helix-loop-helix family, member e40	TR	4.34 / 0.034122
*Trib1*, tribbles pseudokinase 1	S	4.33 / 0.00474
*Coq10b*, coenzyme Q10B	PSHD	4.32 / 0.001009
*Junb*, jun B proto-oncogene	TR	4.22 / 0.00198
*Fosb*, FBJ osteosarcoma oncogene B	TR	4.21 / 0.021086
*Procr*, protein C receptor, endothelial	S	4.12 / 0.030968
*Rd3l*, retinal degeneration 3-like	UN	3.94 / 0.031525
*Per1*, period circadian clock 1	CPD	3.9 / 0.024427
*Cxcl6*, chemokine (C-X-C motif) ligand 6	IR	3.86 / 0.027669
*Emp1*, epithelial membrane protein 1	O	3.68 / 0.033698
*Ccnl1*, cyclin L1	CPD	3.56 / 0.008671
*Dusp6*, dual specificity phosphatase 6	S	3.55 / 0.018941
*Klf4*, Kruppel-like factor 4 (gut)	TR	3.49 / 0.020354
*Spry4*, sprouty RTK signaling antagonist	S	3.48 / 0.004545
*Vgf*, VGF nerve growth factor inducible	CPD	3.32 / 0.023502
*Nfkbia*, nuclear factor of kappa light polypeptide gene enhancer	TR	3.14 / 0.001767
*Atf3*, activating transcription factor 3	TR	3.02 / 0.021591
*Rnf122*, ring finger protein 122	PSHD	2.94 / 0.028845
*Slc25a25*, mitochondrial phosphate carrier	MTR	2.92 / 0.016024
*Rgs16*, regulator of G-protein signaling 16	S	2.89 / 0.027214
*Pnrc1*, proline-rich nuclear receptor coactivator 1	TR	2.87 / 0.005126
*Mstn*, myostatin	IR	2.87 / 0.049177
*Spry1*, sprouty RTK signaling antagonist 1	S	2.84 / 0.031365
*Rnd3*, Rho family GTPase 3	S	2.83 / 0.010714
*Jun*, jun proto-oncogene	TR	2.78 / 0.001458
*Pfkfb3*, 6-phosphofructo-2-kinase/fructose-2,6-biphosphatase 3	S	2.77 / 0.018456
*Rel*, v-rel avian reticuloendotheliosis viral oncogene homolog	TR	2.67 / 0.012933
*Plk3*, polo-like kinase 3	S	2.63 / 0.002178
*Pmaip1*, phorbol-12-myristate-13-acetate-induced protein 1	CPD	2.61 / 0.026542
*Icam1*, intercellular adhesion molecule 1	IR	2.6 / 0.035928
*Gch1*, GTP cyclohydrolase 1	S	2.53 / 0.002142
*Lysmd3*, LysM, putative peptidoglycan-binding	UN	2.48 / 0.034801
*Tmem2*, transmembrane protein 2	O	2.47 / 0.035654
*LOC102552920*, armadillo repeat-containing X-linked protein	UN	2.45 / 0.021451
*Plau*, plasminogen activator, urokinase	PSHD	2.43 / 0.014206
*Arid5b*, AT rich interactive domain 5B (Mrf1-like)	TR	2.35 / 0.02648
*Xirp1*, xin actin-binding repeat containing 1	O	2.35 / 0.039713
*Fgf9*, fibroblast growth factor 9	CPD	2.33 / 0.032372
*Ldlr*, low density lipoprotein receptor	S	2.31 / 0.000417
*Rcan1*, regulator of calcineurin 1	S	2.27 / 0.009104
*Lysmd3*, LysM, putative peptidoglycan-binding	UN	2.21 / 0.017634
*Rgs2*, regulator of G-protein signaling 2	S	2.19 / 0.012669
*Il1ra*, interleukin 1 receptor accessory protein	IR	2.15 / 0.029089
*Slc39a14*, solute carrier family 39 (zinc transporter), member 14	MTR	2.14 / 0.012704
*Thbs2*, thrombospondin 2	IR	2.13 / 0.020824
*Arl5b*, ADP-ribosylation factor-like 5B	S	2.12 / 0.041265
*Gcnt4*, glucosaminyl (N-acetyl) transferase 4, core 2	O	2.12 / 0.006454
*Il1r1*, interleukin 1 receptor, type I	IR	2.11 / 0.041316
*Zfp711*, zinc finger protein 711	TR	2.08 / 0.025828
*Clcn5*, chloride channel, voltage-sensitive 5	MTR	2.06 / 0.020143
*Arrdc3*, arrestin domain containing 3	S	2.05 / 0.012561
*Timp1*, TIMP metallopeptidase inhibitor 1	PSHD	2.05 / 0.01694
*Gucy1a2*, guanylate cyclase 1, soluble, alpha 2	S	2.04 / 0.02076
*Ahr*, aryl hydrocarbon receptor	TR	2.04 / 0.020671
*Alkbh1*, alkB homolog 1, histone H2A dioxygenase	TR	2.02 / 0.046413
*Mxd*, max dimerization protein 1	TR	2.01 / 0.04203
*LOC498465*, similar to RIKEN cDNA 1700001F09	UN	2.01 / 0.011855

The cells were incubated in the absence or presence of 1 mM ouabain for 3 h. For functional categories, see Table 4. All experiments were repeated 3 times.

**Table 7:** Genes whose expression was decreased by 1 mM ouabain by more than 2-fold.

Gene Symbol, Title	Functional Category	Fold of inhibition / p-value
***Downregulated***		
*Otub2*, OTU deubiquitinase, ubiquitin aldehyde binding 2	O	–2.01 / 0.049957
*Wscd1*, WSC domain containing 1	O	–2.01 / 0.014985
*Prkag2*, protein kinase, AMP–activated, gamma 2 subunit	S	–2.01 / 0.039038
*Mki67*, marker of proliferation Ki-67	UN	–2.03 / 0.036377
*Mavs*, mitochondrial antiviral signaling protein	IR	–2.1 / 0.031824
*Eya2*, EYA transcriptional coactivator and phosphatase 2	TR	–2.13 / 0.035164
*Zfp703*, zinc finger protein 703	TR	–2.16 / 0.047628
*RGD1308147*, similar to expressed sequence AW209491	UN	–2.19 / 0.000092
*Mir181b1*, microRNA 181b-1	miRNA	–2.21 / 0.038541
*Ptpn14*, protein tyrosine phosphatase, non-receptor type 14	S	–2.21 / 0.03322
*Ddit4l*, DNA-damage-inducible transcript 4-like	S	–2.23 / 0.029361
*Mir421*, microRNA 421	miRNA	–2.24 / 0.019258
*Nudt13*, nudix (nucleoside diphosphate linked moiety X)-type	O	–2.25 / 0.020326
*Mir410*, microRNA 410	miRNA	–2.27 / 0.000698
*Rabif (RGD1563962)*, RAB interacting factor	S	–2.28 / 0.03624
*Pth1r*, parathyroid hormone 1 receptor	S	–2.29 / 0.000959
*Gdpgp1*, GDP-D-glucose phosphorylase 1	O	–2.31 / 0.010957
*Vof16*, ischemia related factor vof-16	UN	–2.35 / 0.006096
*LOC100359748*, zinc finger CCCH type, antiviral 1	O	–2.36 / 0.004982
*Mir544*, microRNA 544	miRNA	–2.36 / 0.010114
*Grtp1*, growth hormone regulated TBC protein 1	S	–2.41 / 0.036684
*Iqub*, IQ motif and ubiquitin domain containing	S	–2.42 / 0.027833
*Rcor2 (Rcor2l1)*, REST corepressor 2	TR	–2.44 / 0.035033
*Rxra*, retinoid X receptor alpha	CPD	–2.48 / 0.028688
*Adgra1*, adhesion G protein-coupled receptor A1	S	–2.48 / 0.000943
*Mir434*, microRNA 434	miRNA	–2.51 / 0.004484
*Krcc1*, lysine-rich coiled-coil 1	TR	–2.57 / 0.012588
*Atoh8*, atonal bHLH transcription factor 8	TR	–2.68 / 0.03377

The cells were incubated in the absence or presence of 1 mM ouabain for 3 h. For functional categories, see Table 4. All experiments were repeated 3 times.

Unlike for low dose, for 1 mM ouabain the list of differentially expressed genes was abundant in intermediates of intracellular signaling, transcription regulators, and proteins involved in inflammatory and immune responses (26%, 25%, and 11%, respectively) (Tables 6 and 7; Fig 3B). Among transcription regulators whose expression was increased more than 3-fold, we found *Npas4*, *Zfp36*, *Klf6*, *Fosb*, *Junb*, *Klf4*, *Nfkbia*, and *Atf3* (Table 6). We also observed a modest elevation of mRNAs encoding several other immediate-early-response genes (IERGs) including cAMP responsive element transcription regulator *(Crem)* and CREB/ATF bZIP transcription factor *(Crebzf)* (S2 Table).

The *Zfp36 (TTP)* gene encodes an RNA-binding protein that regulates the metabolism of mRNAs by targeting them for degradation. TTP interacts with many mRNAs encoding a wide range of growth factors, cytokines, and proto-oncogenes and destabilizes them. It has been shown that TTP downregulation is necessary for proper neuronal differentiation *in vitro* and might be a novel post-transcriptional repressor of nervous-system-specific genes that participate during brain development [47].

Activating transcription factor 3 (*Atf3*) belongs to the ATF/CREB transcription factor family involved in cell growth and apoptosis. It has been shown that ATF3 is a downstream target of the JNK/c-Jun pathway and contributes to apoptosis induced by potassium deprivation in rat cerebellum cell culture [48]. Numerous studies have demonstrated that augmented expression of these genes might be involved in learning and memory consolidation via modulation of synaptic plasticity and adult hippocampal neurogenesis [49, 50]. Another upregulated IERG Kruppel-like factor 4 (*Klf4*) is also involved in cell proliferation, differentiation, and death. Zhu et al demonstrated that glutamatergic stimulation of cultured cortical neurons results in overexpression of KLF4, which regulates cell cycle proteins and sensitizes neurons to NMDA-induced caspase-3 activity [51].

*Bdnf* encodes a neurotrophin that plays a key role in neuronal development [52, 53]. We observed that 1 mM ouabain evokes 13-fold increase of *Npas4*, which was found to be associated with *Bdnf* promoters I and IV (Table 6). It has been shown that Npas4 expression is rapidly activated by excitatory synaptic activity and turns on a program of gene expression that triggers the formation and/or maintenance of inhibitory synapses on excitatory neurons [54].

Table 3 shows that the list of transcripts affected by both 100 nM and 1 mM ouabain is limited to two down-regulated genes (*Oxsm* and *OC685989*). Based on the UniProt database, Oxsm protein may be involved in biosynthesis of lipoic acid as well as longer chain fatty acids required for optimal mitochondrial function [55]. The function of hypothetical OC685989 protein remains unknown.

**Fig 4:**
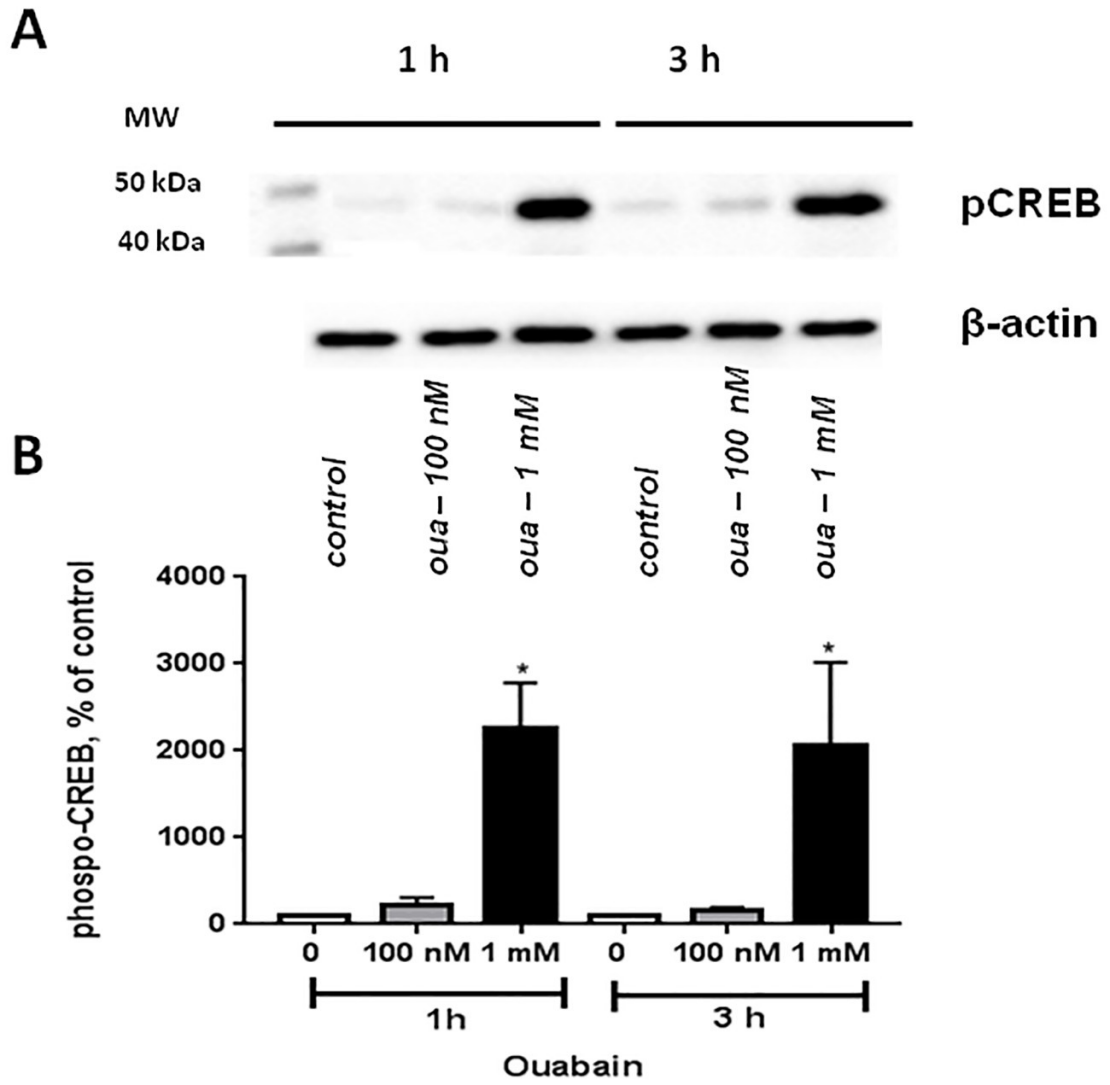
Effect of ouabain (oua) on CREB phosphorylation in cerebellum granule cells. A representative Western blot (**A**) and means ± SD for 4 independent experiments (**B**) are shown. *–p<0.05 compared to control.

Transcriptomic changes in cerebellum granule cells treated with 1 mM ouabain are probably mediated by inhibition of α1-Na^+^,K^+^-ATPase and elevation of the [Na^+^]_i_/[K^+^]_i_ ratio. This conclusion is supported by comparative analysis of dose- and time-dependent action of ouabain and another cardiotonic steroid, marinobufagenin, on intracellular content of Na^+^ and K^+^ and gene expression in human endothelial cells abundant with α1-Na^+^,K^+^-ATPase [56]. In several types of cells, elevation of the [Na^+^]_i_/[K^+^]_i_ ratio increases [Ca^2+^]_i_ via activation of an Na^+^/Ca^2+^ exchanger [57] and/or voltage-gated Ca^2+^ channel [58]. Thus, it might be assumed that excitation–transcription coupling triggered by high doses of ouabain is “at least partially” driven by changes in [Ca^2+^]_i_ and activation of several Ca^2+^-mediated pathways including phosphorylation of cAMP response-element-binding protein CREB (for reviews, see [59, 60]). Fig 4 shows that 1 h exposure to 1 mM ouabain increased CREB phosphorylation by ~20-fold, thus suggesting an impact of Ca^2+^- and/or cAMP-mediated signaling.

**Fig 5:**
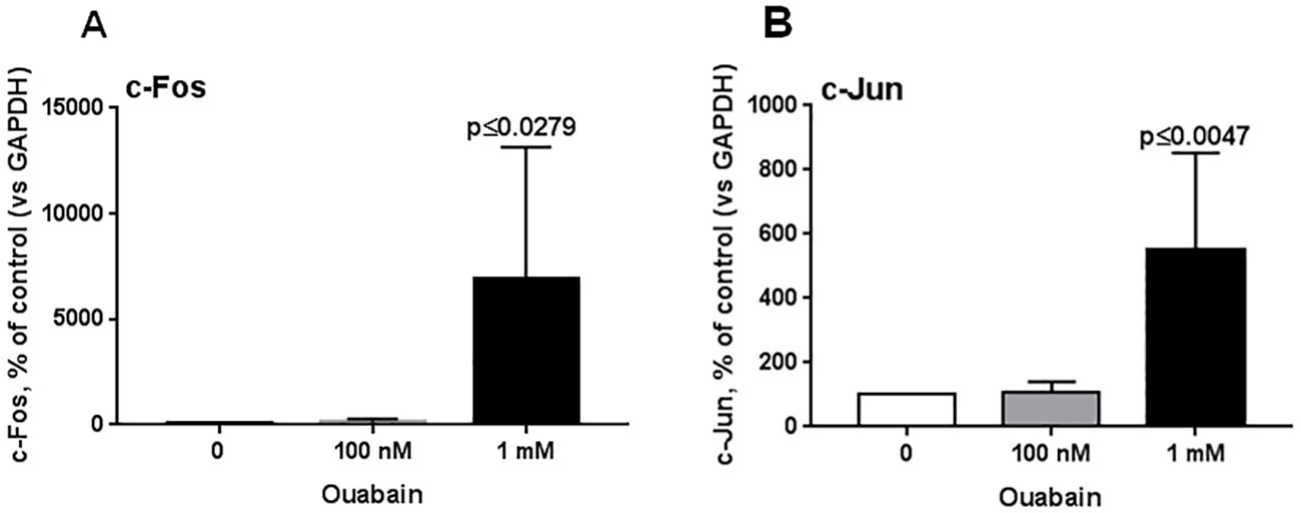
Expression of *c-Fos* (A) and *c-Jun* (B) mRNAs in cerebellum granule cells after 3 h of incubation with 100 nM or 1 mM ouabain. Data obtained in 5 independent experiments are presented as the mean ± SD. Gene expression in the absence of ouabain was taken as 100%.

The presence of cAMP-response element (CRE) within promoters of diverse IERGs including *c-Fos* and *c-Jun B* is well-documented [61, 62]. Consistent with microarray data (Table 6), we documented a sharp increment of these gene transcripts in cells treated with 1 mM ouabain using the qPCR approach (Fig 5). It should be noted, however, that in vascular smooth muscle and endothelial cells, augmented expression of these and several other IERGs were preserved in the presence of extra- and intracellular Ca^2+^ chelators [63–65] and selective inhibitors of calmodulin and Ca^2+^-sensitive protein kinase and phosphatases [58]. Thus, additional experiments should be performed to examine the relative impact of [Ca^2+^]_i_-dependent and -independent signaling in transcriptomic changes triggered in rat cerebellum granule cells by α1-Na^+^,K^+^-ATPase-mediated elevation of the [Na^+^]_i_/[K^+^]_i_ ratio.

Considering the physiological implications of the extended [Na^+^]_i_,[K^+^]_i_-sensitive transcriptome identified in our study, it should be noted that side-by-side with α1-Na^+^,K^+^-ATPase inhibition, dissipation of transmembrane gradients of monovalent cations might be triggered by activation of monovalent cation-permeable ionotropic AMPA receptors [66] and voltage-gated Na^+^ channels [67, 68]. Indeed, using acute tissue slices of mouse cerebellum loaded with fluorescent Na^+^ indicators, it was shown that in dendrites of Purkinje neurons glutamate transiently increases [Na^+^]_i_ by 5–10 mM via activation of AMPA receptors [69]. In Purkinje neurons, transient increments of [Na^+^]_i_ were also evoked by brief (50 Hz/100 ms) electrical pulse stimulation [46]. In dopamine cells of the substantia nigra pars compacta, electrical hyperpolarization induced a rise in [Na^+^]_i_ from 10 to 25 mM [70]. Because NMDA receptors exhibit low cation selectivity (P_Na_ ~ P_K_> P_Ca_), they also contribute to elevation of the [Na^+^]_i_/[K^+^]_i_ ratio triggered by synaptic excitation. Consistent with these data, selective activation of NMDA receptors in rat cerebellar granule cells resulted in reversible elevation of [Na^+^]_i_ from 5 to 60 mM [71]. Using cultured cerebellar neurons, Linden et al. showed that an increase in [Na^+^]_i_ rather than [Ca^2+^]_i_ is required for the induction of long-term depression [72].

Modest transcriptomic changes evoked by 100 nM ouabain suggest implication of the α3 isoform as an upstream intermediate of signal transduction. Importantly, at this concentration, ouabain did not affect intracellular content of monovalent cations (Table 1). Recent studies have revealed that CTSs can affect cells independently of suppression of Na^+^,K^+^-ATPase-mediated ion fluxes. Thus, at low concentrations ouabain triggered the interaction of the Na^+^,K^+^-ATPase α-subunit with the membrane-associated nonreceptor tyrosine kinase Src, activation of Ras/Raf/ERK1,2, phosphatidyl inositol 3-kinase (PI(3)K), PI(3)K-dependent protein kinase B, phospholipase C, and [Ca^2+^]_i_, and oscillations and augmented production of reactive oxygen species (for reviews, see [16, 17, 19, 73]). Earlier it was shown that in rat cerebellum granule cells, 100 nM ouabain activates MAP kinase via PKC and PIP(3) kinase, whereas 1 mM ouabain regulates this MAP kinase signaling cascade via an Src kinase-dependent pathway [74]. Unlike 1 mM ouabain, we did not detect any elevation of CREB phosphorylation in the presence of 100 nM ouabain (Fig 4). The role of other intermediates of [Na^+^]_i_/[K^+^]_i_-independent signaling in transcriptomic changes triggered by low doses of ouabain should be examined in forthcoming studies.

In conclusion, we report here that complete inhibition of Na^+^,K^+^-ATPase by 1 mM ouabain results in dissipation of transmembrane Na^+^ and K^+^ gradients, whereas selective inhibition of α3- Na^+^,K^+^-ATPase by 100 nM ouabain has no impact on the [Na^+^]_i_/[K^+^]_i_ ratio. Among hundreds of differentially expressed transcripts detected in the presence of high and low doses of ouabain, we identified only 2 common genes, thus indicating different mechanisms of excitation–transcription coupling mediated by α1- and α3-Na^+^,K^+^-ATPase. Among genes sharply upregulated by 1 mM ouabain, we found *Npas4*, *Fos*, *Junb*, *Atf3*, and *Klf4* mRNAs, whose augmented expression was demonstrated in neurons. The list of genes affected by 100 nM ouabain is abundant in olfactory receptors, whose function in neuronal cells remains poorly understood. Downstream mediators of [Na^+^]_i_/[K^+^]_i_-independent excitation–transcription coupling signaling triggered by interaction of ouabain with α3-Na^+^,K^+^-ATPase remains unknown.

## Supporting information

S1 Fig Summary of upregulated gene sets (GeneOntology—Biological process) in 1mM ouabain-treated granular neurons. (TIF)

S2 Fig Summary of downregulated gene sets (GeneOntology—Biological process) in 1mM ouabain-treated granular neurons. (TIF)

S3 Fig Summary of upregulated gene sets (GeneOntology—Molecular function) in 1mM ouabain-treated granular neurons. (TIF)

S4 Fig Summary of downregulated gene sets (GeneOntology—Molecular function) in 1mM ouabain-treated granular neurons. (TIF)

S5 Fig Summary of upregulated gene sets (GeneOntology—Cellular component) in 1mM ouabain-treated granular neurons. (TIF)

S6 Fig Summary of downregulated gene sets (GeneOntology—Cellular component) in 1mM ouabain-treated granular neurons. (TIF)

S7 Fig Summary of upregulated gene sets (GeneOntology—Biological process) in 100nM ouabain-treated granular neurons. (TIF)

S8 Fig Summary of downregulated gene sets (GeneOntology—Biological process) in 100nM ouabain-treated granular neurons. (TIF)

S9 Fig Summary of upregulated gene sets (GeneOntology—Molecular function) in 100nM ouabain-treated granular neurons. (TIF)

S10 Fig Summary of downregulated gene sets (GeneOntology—Molecular function) in 100nM ouabain-treated granular neurons. (TIF)

S11 Fig Summary of upregulated gene sets (GeneOntology—Cellular component) in 100nM ouabain-treated granular neurons. (TIF)

S12 Fig Summary of downregulated gene sets (GeneOntology—Cellular component) in 100nM ouabain-treated granular neurons. (TIF)

S1 Table Transcripts whose expression was change by more than 1.3-fold by 100 nM ouabain. (PDF)

S2 Table Transcripts whose expression was change by more than 1.3-fold by 1 mM ouabain. (PDF)

S3 Table Upregulated gene sets (GeneOntology—Biological process) in 1mM ouabain-treated granular neurons significant at FDR < 1%. (DOCX)

S4 Table Downregulated gene sets (GeneOntology—Biological process) in 1mM ouabain-treated granular neurons significant at FDR < 1%. (DOCX)

S5 Table Upregulated gene sets (GeneOntology—Molecular function) in 1mM ouabain-treated granular neurons significant at FDR < 1%. (DOCX)

S6 Table Downregulated gene sets (GeneOntology—Molecular function) in 1mM ouabain-treated granular neurons significant at FDR < 1%. (DOCX)

S7 Table Upregulated gene sets (GeneOntology—Cellular component) in 1mM ouabain-treated granular neurons significant at FDR < 1%. (DOCX)

S8 Table Downregulated gene sets (GeneOntology—Cellular component) in 1mM ouabain-treated granular neurons significant at FDR < 1%. (DOCX)

S9 Table Upregulated gene sets (GeneOntology—Biological process) in 100nM ouabain-treated granular neurons at NES < -1.35. (DOCX)

S10 Table Downregulated gene sets (GeneOntology—Biological process) in 100nM ouabain-treated granular neurons at NES > 1.35. (DOCX)

S11 Table Upregulated gene sets (GeneOntology—Molecular function) in 100nM ouabain-treated granular neurons at NES < -1.35. (DOCX)

S12 Table Downregulated gene sets (GeneOntology—Molecular function) in 100nM ouabain-treated granular neurons at NES > 1.35. (DOCX)

S13 Table Upregulated gene sets (GeneOntology—Cellular component) in 100nM ouabain-treated granular neurons at NES < -1.35. (DOCX)

S14 Table Downregulated gene sets (GeneOntology—Cellular component) in 100nM ouabain-treated granular neurons at NES > 1.35. (DOCX)
