# Supplementary figures and images for: Transcriptomic changes triggered by ouabain in rat cerebellum granule cells: Role of α3- and α1-Na+,K+-ATPase-mediated signaling
Source: PLoS One. 2019 Sep 26;14(9):e0222767. doi: 10.1371/journal.pone.0222767 (PMC6762055; doi:10.1371/journal.pone.0222767)

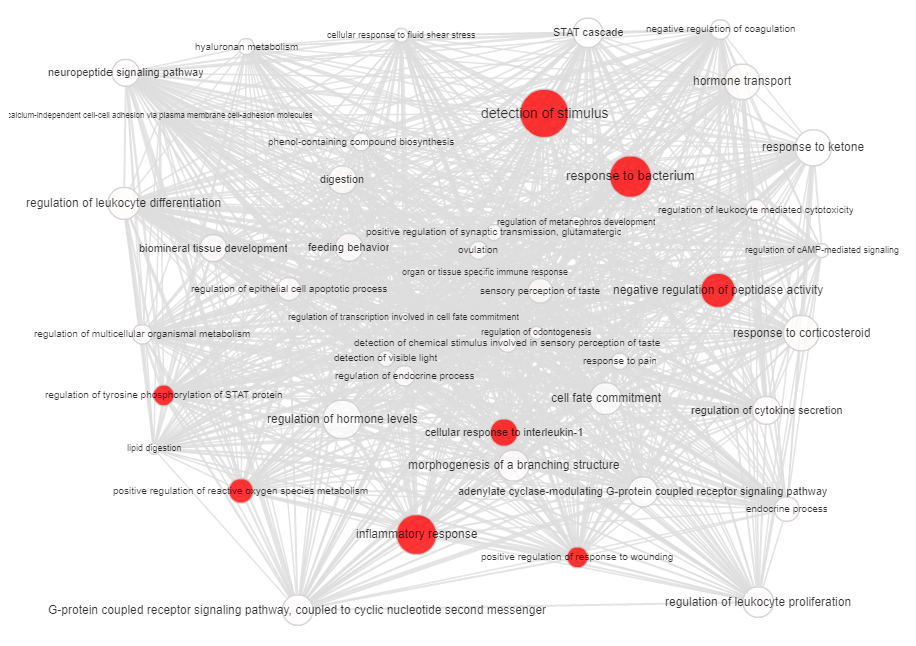

Supplement: S1 Fig — (TIF) [file pone.0222767.s001.tif]

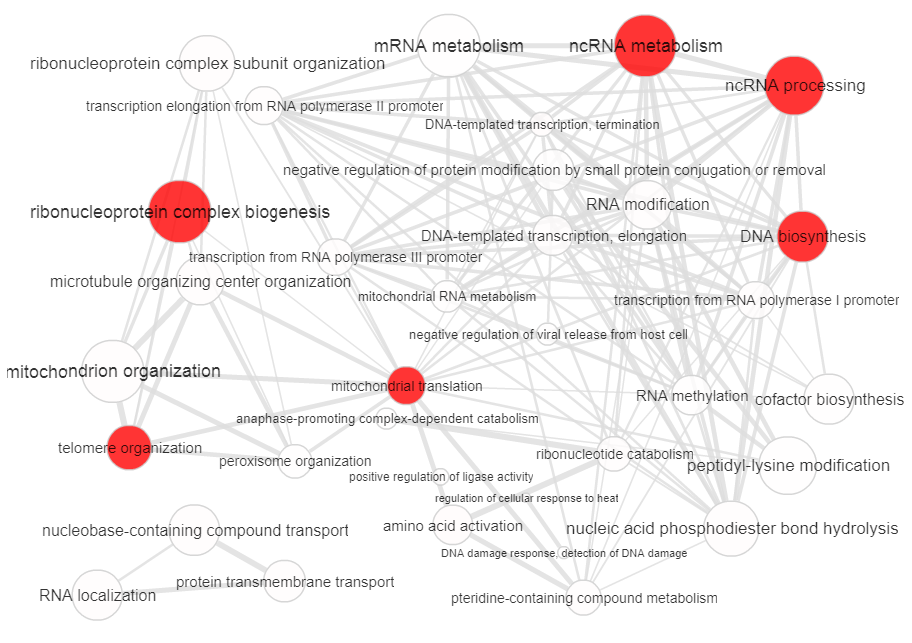

Supplement: S2 Fig — (TIF) [file pone.0222767.s002.tif]

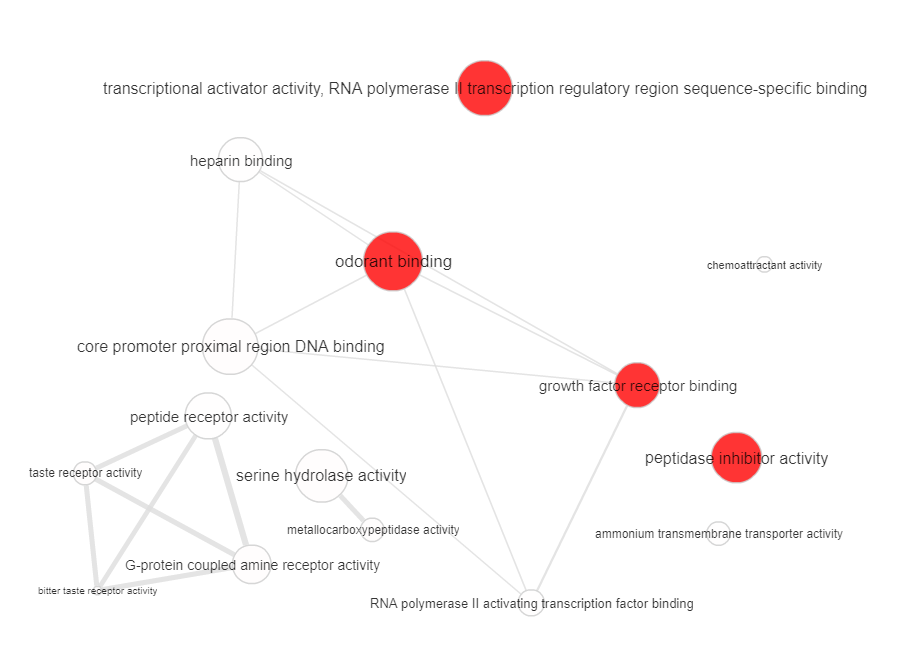

Supplement: S3 Fig — (TIF) [file pone.0222767.s003.tif]

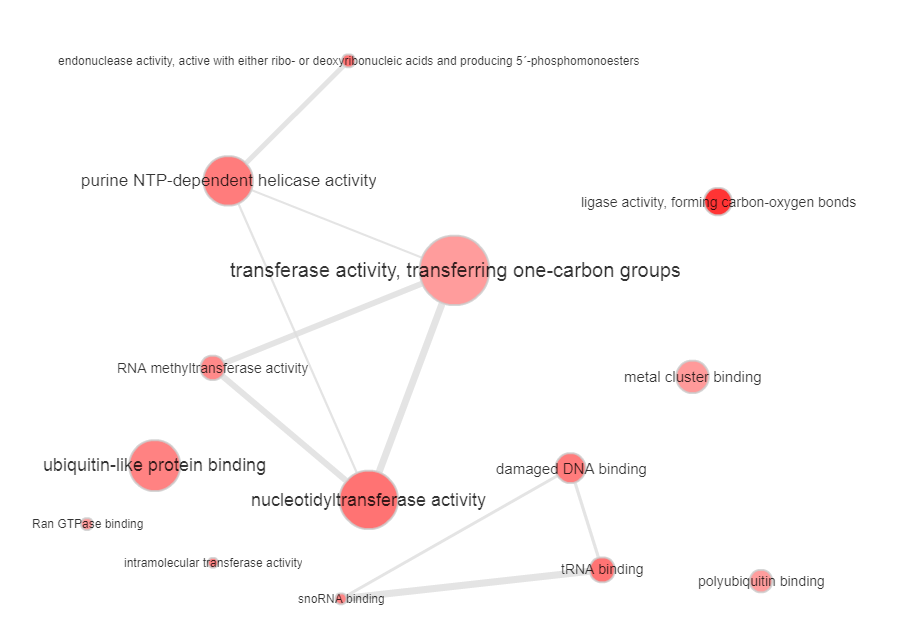

Supplement: S4 Fig — (TIF) [file pone.0222767.s004.tif]

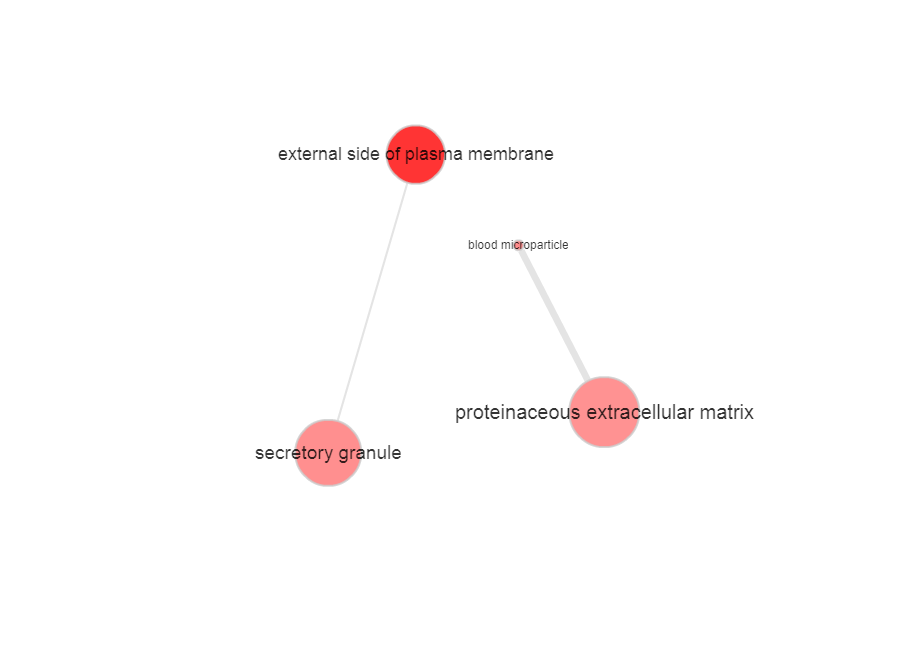

Supplement: S5 Fig — (TIF) [file pone.0222767.s005.tif]

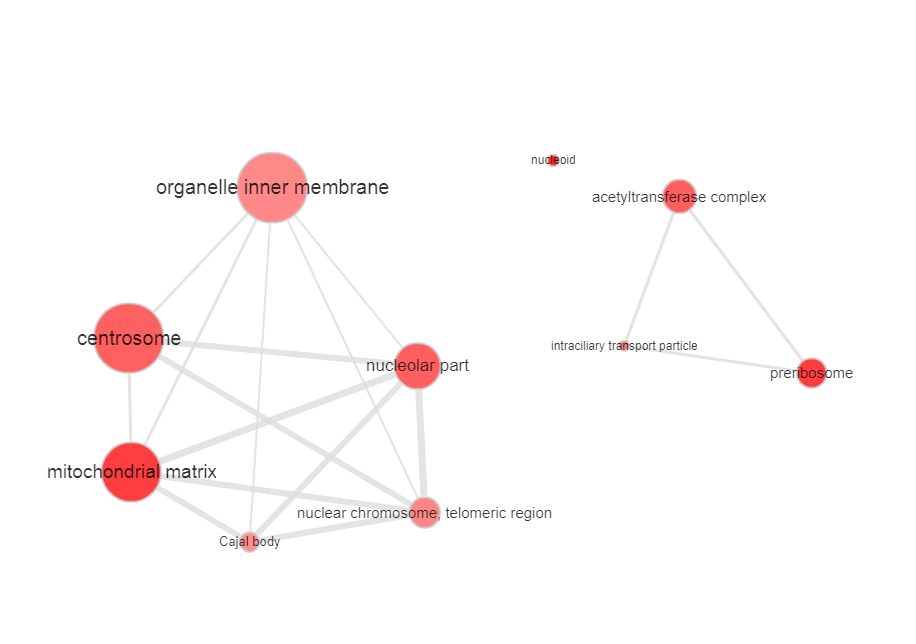

Supplement: S6 Fig — (TIF) [file pone.0222767.s006.tif]

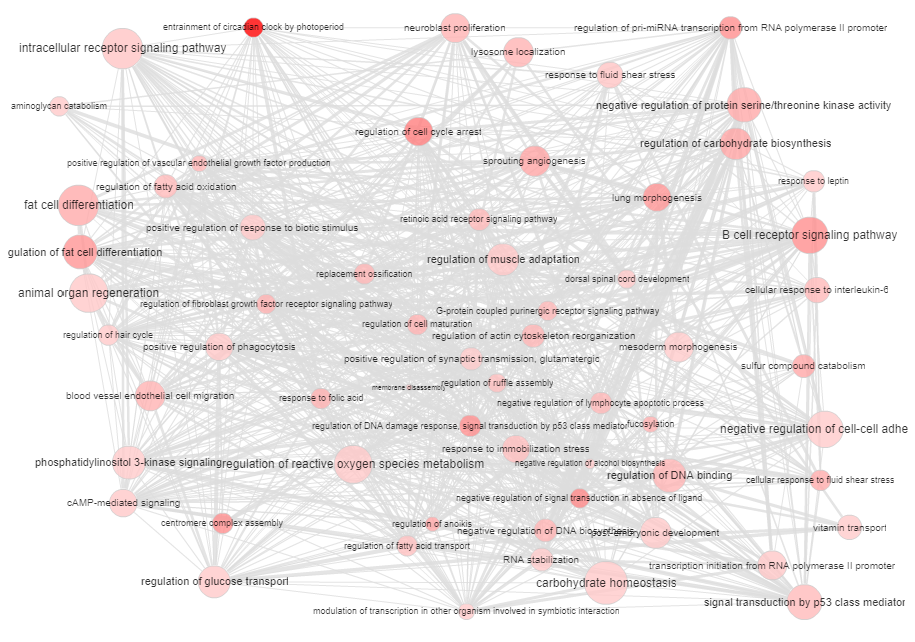

Supplement: S7 Fig — (TIF) [file pone.0222767.s007.tif]

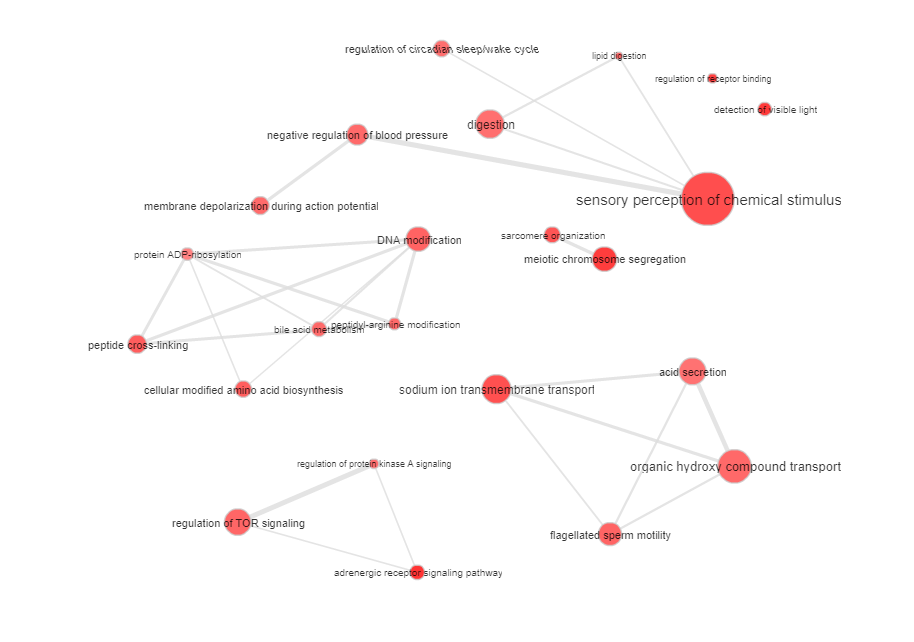

Supplement: S8 Fig — (TIF) [file pone.0222767.s008.tif]

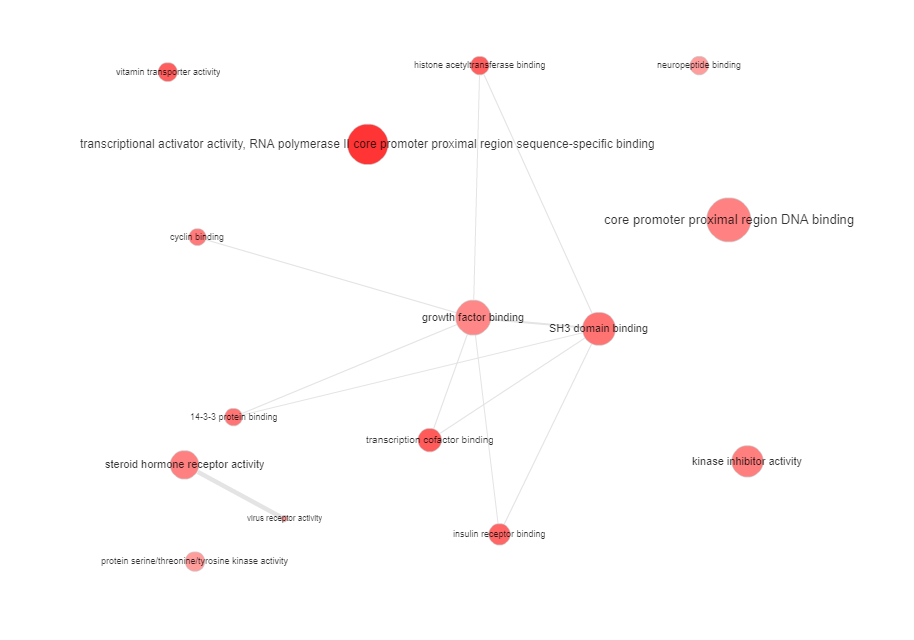

Supplement: S9 Fig — (TIF) [file pone.0222767.s009.tif]

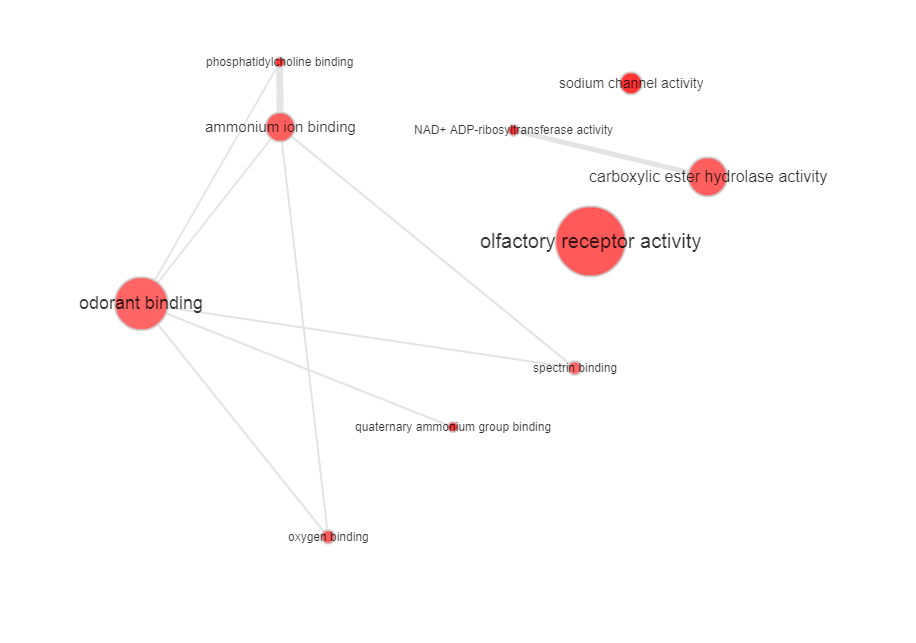

Supplement: S10 Fig — (TIF) [file pone.0222767.s010.tif]

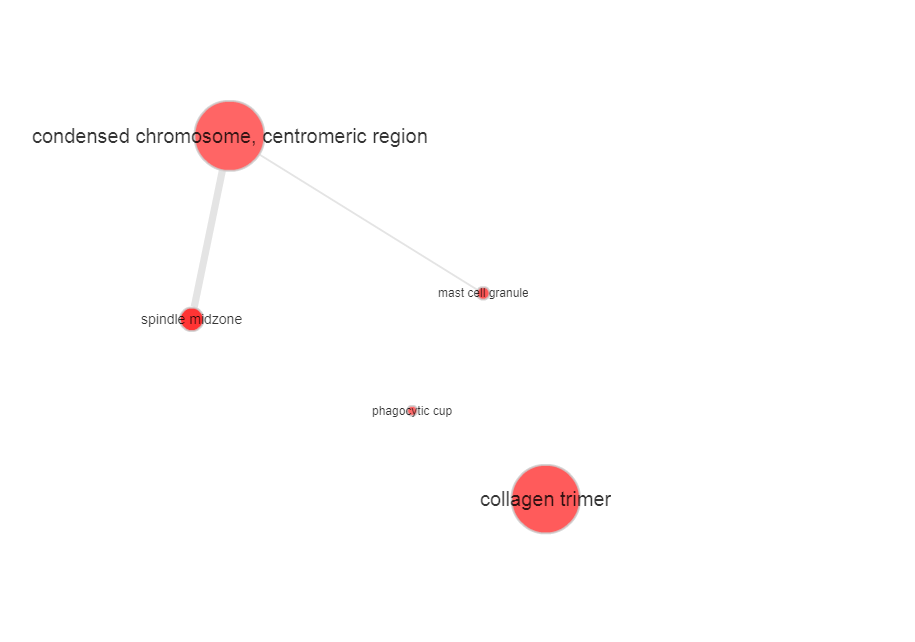

Supplement: S11 Fig — (TIF) [file pone.0222767.s011.tif]

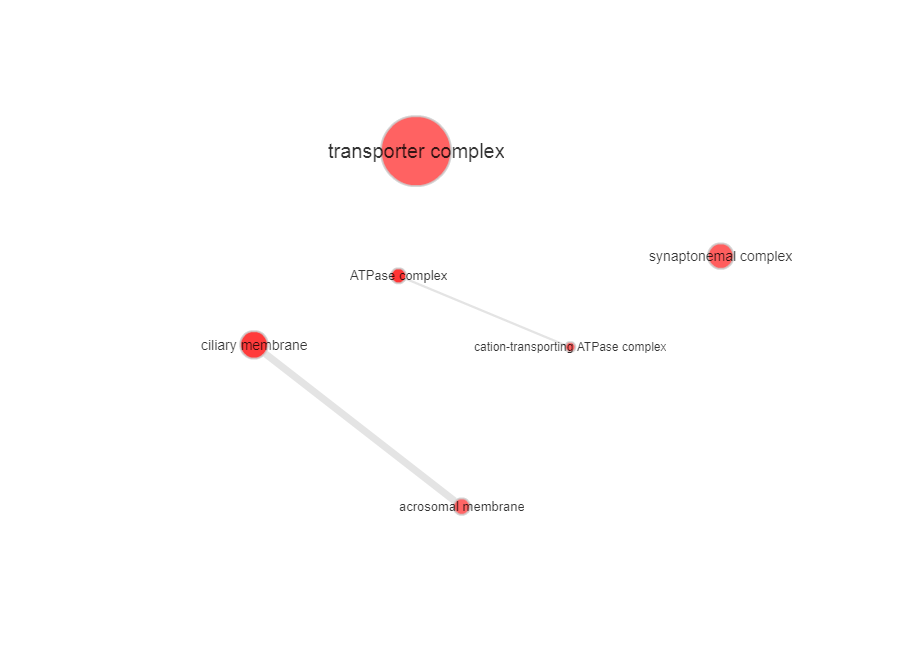

Supplement: S12 Fig — (TIF) [file pone.0222767.s012.tif]
