# Supplementary material for: Transcriptomic changes triggered by ouabain in rat cerebellum granule cells: Role of α3- and α1-Na+,K+-ATPase-mediated signaling
Source: PLoS One. 2019 Sep 26;14(9):e0222767. doi: 10.1371/journal.pone.0222767 (PMC6762055; doi:10.1371/journal.pone.0222767)
Supplement: S1 Table — (PDF) [file pone.0222767.s013.pdf]

Table S1. Transcripts whose expression was change by more than 1.3-fold by 100 nM ouabain.

Probe Set ID	Gene Symbol	Gene Description	Fold Change (100 nM vs. Control)	p-value
17883209	---	---	3,22	0,048002
17867328	---	---	1,89	0,016802
17774208	*Olr551*	olfactory receptor 551	1,87	0,018489
17829201	---	---	1,74	0,044548
17724289	---	---	1,74	0,028494
17628668	---	---	1,71	0,045111
17775348	---	---	1,66	0,01531
17882929	---	---	1,66	0,01531
17665497	---	---	1,63	0,035465
17850348	*Spc24*	SPC24, NDC80 kinetochore complex component	1,55	0,04222
17668363	---	---	1,54	0,014277
17828153	---	---	1,51	0,035098
17851367	*Olr1334*	olfactory receptor 1334	1,5	0,037193
17701205	---	---	1,49	0,000407
17759128	*RGD1310495*	similar to KIAA1919 protein	1,47	0,044616
17761757	*Olr397*	olfactory receptor 397	1,47	0,014367
17880303	*LOC102548248*	calphotin-like	1,47	0,032125
17831014	*Csdc2*	cold shock domain containing C2, RNA binding	1,45	0,033715
17862391	---	---	1,45	0,008313
17645350	*Olr1394*	olfactory receptor 1394	1,44	0,032407
17628078	*LOC102556805*	rho GTPase-activating protein 20-like	1,43	0,023919
17882769	---	---	1,43	0,044904
17839270	---	---	1,43	0,03685
17706353	*Drgx*	dorsal root ganglia homeobox	1,41	0,044668
17827557	---	---	1,4	0,04706
17675699	---	---	1,39	0,042268
17787401	*Akap3*	A kinase (PRKA) anchor protein 3	1,39	0,026649
17646852	*Map2k4*	mitogen activated protein kinase kinase 4	1,38	0,01881
17706772	*LOC498601*	similar to cyclin B2	1,38	0,03072

17835259	*Snrpf*	small nuclear ribonucleoprotein polypeptide F	1,38	0,003448
17686822	---	---	1,37	0,03483
17739417	---	---	1,37	0,036329
17615597	---	---	1,37	0,029837
17877579	*Mageb16*	melanoma antigen family B, 16	1,37	0,006286
17703808	*Pnoc*	prepronociceptin	1,37	0,016375
17611071	---	---	1,36	0,033265
17734750	*LOC100361079*	ribosomal protein L36-like	1,36	0,007825
17880086	*Emd*	emerin	1,35	0,034983
17790469	---	---	1,35	0,020103
17875397	---	---	1,35	0,03943
17802806	---	---	1,35	0,025503
17793044	*Tcp1-ps1*	t-complex protein 1, pseudogene 1	1,35	0,00873
17804598	*Rpl22*	ribosomal protein L22	1,35	0,018718
17716731	*Akr1c12*	aldo-keto reductase family 1, member C12	1,35	0,031111
17707675	---	---	1,34	0,012088
17700524	---	---	1,34	0,034992
17869228	*Rpl36*	ribosomal protein L36	1,34	0,043525
17625633	---	---	1,34	0,009297
17651686	---	---	1,34	0,017883
17861924	*Fam174a*	family with sequence similarity 174, member A	1,33	0,028861
17624337	*Olr352*	olfactory receptor 352	1,33	0,021153
17713990	*Ctla2a*	cytotoxic T lymphocyte-associated protein 2 alpha	1,33	0,020505
17713951	*MGC114246*	similar to cathepsin R	1,33	0,045924
17765498	---	---	1,33	0,011225
17880941	*LOC102551267*	paired box protein Pax-7-like	1,32	0,001153
17778090	*LOC296235*	similar to Cystatin S precursor (LM protein)	1,32	0,010687
17849826	---	---	1,32	0,033884
17822958	---	---	1,32	0,038197

17717113	*Rpl34-ps1*	ribosomal protein L34, pseudogene 1 [Source:RGD Symbol;Acc:1595454]	1,32	0,049551
17730423	*Atmin*	ATM interactor	1,32	0,044838
17650228	---	---	1,32	0,02478
17670194	*RGD1559972*	similar to ribosomal protein L27a [Source:RGD Symbol;Acc:1559972]	1,32	0,034465
17870837	---	---	1,32	0,048634
17715631	*Acot13*	acyl-CoA thioesterase 13	1,32	0,047266
17854574	---	---	1,32	0,008048
17664979	*Pcp4*	Purkinje cell protein 4	1,31	0,024075
17743773	---	---	1,31	0,040693
17629023	---	---	1,31	0,010947
17629290	*Mir290*	microRNA 290	1,3	0,031793
17868073	---	---	1,3	0,014677
17827534	---	---	1,3	0,01285
17778749	*Scand1*	SCAN domain-containing 1	1,3	0,049441
17744890	*Ccnb1*	cyclin B1	1,3	0,0175
17616851	*Ano5*	anoctamin 5	-1,3	0,019476
17645348	*Olr1393*	olfactory receptor 1393	-1,3	0,007599
17760877	*Pkn3*	protein kinase N3	-1,3	0,006272
17774230	*Olr587*	olfactory receptor 587	-1,3	0,035127
17645575	*Olr1410*	olfactory receptor 1410	-1,31	0,02515
17734418	*Cox6c-ps1*	cytochrome c oxidase subunit VIc, pseudogene	-1,31	0,016536
17805284	---	---	-1,31	0,0218
17793954	---	---	-1,31	0,037151
17862643	*Usp49*	ubiquitin specific peptidase 49	-1,31	0,036476
17844336	*Tmem266*	transmembrane protein 266	-1,31	0,02683
17670584	*Gnb1l*	guanine nucleotide binding protein (G protein), beta polypeptide 1-like	-1,31	0,005584
17828197	---	---	-1,32	0,02701
17676156	---	---	-1,32	0,01348
17837395	*Sntb1*	syntrophin, beta 1	-1,32	0,027004

17841133	---	---	-1,32	0,013165
17833289	*LOC102549817*	zinc finger protein 124-like	-1,33	0,047193
17859903	*LOC103690541*	uncharacterized LOC103690541	-1,33	0,011505
17668348	---	---	-1,33	0,018842
17698585	*RGD1311300*	similar to T cell receptor V delta 6	-1,34	0,014249
17646341	---	---	-1,34	0,034687
17821629	*Lrrc72*	leucine rich repeat containing 72	-1,34	0,00591
17784285	---	---	-1,34	0,026258
17697472	*Oxsm*	3-oxoacyl-ACP synthase, mitochondrial	-1,34	0,030341
17755938	*Olr1695*	olfactory receptor 1695	-1,35	0,034048
17624370	---	---	-1,35	0,006393
17667606	*Cyyr1*	cysteine/tyrosine-rich 1	-1,35	0,037929
17797711	---	---	-1,36	0,032131
17654068	*Olr1374*	olfactory receptor 1374	-1,36	0,031302
17778340	*Defb25*	defensin beta 25	-1,36	0,016742
17699515	*Defb41*	defensin beta 41	-1,36	0,042335
17698463	---	---	-1,36	0,014
17857312	---	---	-1,36	0,007646
17863598	*Spata31e1*	SPATA31 subfamily E, member 1	-1,36	0,0433
17834586	*Ric8b*	RIC8 guanine nucleotide exchange factor B	-1,37	0,042087
17835175	---	---	-1,37	0,016033
17868846	*LOC689679*	similar to Discs large homolog 5 (Placenta and prostate DLG) (Discs large protein P-dlg)	-1,37	0,010808
17648339	---	---	-1,37	0,005315
17698268	---	---	-1,37	0,005147
17681401	---	---	-1,37	0,020181
17870189	*LOC685989*	hypothetical protein LOC685989	-1,37	0,036192
17681399	---	---	-1,39	0,04931
17766669	*Snap25*	synaptosomal-associated protein 25	-1,39	0,036352
17853199	---	---	-1,39	0,043556
17740451	---	---	-1,39	0,046225

17842286	*Olr1192*	olfactory receptor 1192	-1,39	0,00279
17611410	---	---	-1,39	0,040678
17715873	*Zkscan3*	zinc finger with KRAB and SCAN domains 3	-1,39	0,02762
17635997	*Olr1875*	olfactory receptor 1875	-1,4	0,039398
17850566	---	---	-1,4	0,036517
17708174	---	---	-1,4	0,049278
17872330	*Cdkl5*	cyclin-dependent kinase-like 5	-1,41	0,008764
17714278	---	---	-1,41	0,007355
17869576	---	---	-1,42	0,020479
17774321	*Olr687*	olfactory receptor 687	-1,42	0,015524
17882681	---	---	-1,44	0,048231
17763726	*Olr639*	olfactory receptor 639	-1,44	0,03603
17825926	*Olr1065*	olfactory receptor 1065	-1,44	0,033742
17755350	---	---	-1,44	0,036144
17698348	---	---	-1,45	0,022814
17854670	---	---	-1,47	0,045076
17757496	*RGD1566085*	similar to pyridoxal (pyridoxine, vitamin B6) kinase	-1,47	0,044618
17633545	---	---	-1,47	0,003783
17880818	---	---	-1,52	0,037503
17878407	---	---	-1,52	0,000618
17712092	---	---	-1,53	0,020509
17843308	*Olr1246*	olfactory receptor 1246	-1,54	0,015191
17786706	*Slc6a12*	solute carrier family 6 (neurotransmitter transporter), member 12	-1,56	0,015467
17845633	---	---	-1,6	0,039133
17868457	---	---	-1,78	0,003186
17697276	---	---	-1,81	0,003875
17761963	---	---	-2,14	0,032177
