# Supplementary material for: Transcriptomic changes triggered by ouabain in rat cerebellum granule cells: Role of α3- and α1-Na+,K+-ATPase-mediated signaling
Source: PLoS One. 2019 Sep 26;14(9):e0222767. doi: 10.1371/journal.pone.0222767 (PMC6762055; doi:10.1371/journal.pone.0222767)
Supplement: S2 Table — (PDF) [file pone.0222767.s014.pdf]

Table S2. Transcripts whose expression was change by more than 1.3-fold by 1 mM ouabain.

Probe Set ID	Gene Symbol	Gene Description	Fold Change (1mM vs. Control)	p-value
17788345	*Il6*	interleukin 6	18,82	0,017424
17680795	*Ptgs2*	prostaglandin-endoperoxide synthase 2	16,59	0,00836
17789522	*Tfpi2*	tissue factor pathway inhibitor 2	15,82	0,035903
17639411	*Npas4*	neuronal PAS domain protein 4	12,97	0,013906
17797288	*Gem*	GTP binding protein overexpressed in skeletal muscle	8,45	0,014514
17751869	*Cyr61*	cysteine-rich, angiogenic inducer, 61	8,09	0,005394
17631103	*Zfp36*	zinc finger protein 36	7,56	0,032644
17649432	*Ccl7*	chemokine (C-C motif) ligand 7	6,88	0,002458
17837405	*Has2*	hyaluronan synthase 2	6,84	0,001877
17882157	---	---	5,92	0,006169
17693465	*Cxcl1*	chemokine (C-X-C motif) ligand 1	5,62	0,014029
17720472	*Klf6*	Kruppel-like factor 6	5,52	0,025668
17667616	*Adamts1*	ADAM metallopeptidase with thrombospondin type 1 motif, 1	5,08	0,005289
17739018	*Ptx3*	pentraxin 3, long	5,02	0,00991
17719769	*Inhba*	inhibin beta-A	4,94	0,018744
17882681	---	---	4,91	0,000599
17785849	*Bhlhe40*	basic helix-loop-helix family, member e40	4,34	0,034122
17829670	*Trib1*	tribbles pseudokinase 1	4,33	0,00474
17859270	*Coq10b*	coenzyme Q10B	4,32	0,001009
17728638	*Junb*	jun B proto-oncogene	4,22	0,00198
17630236	*Fosb*	FBJ osteosarcoma oncogene B	4,21	0,021086
17768032	*Procr*	protein C receptor, endothelial	4,12	0,030968
17824495	*Rd3l*	retinal degeneration 3-like	3,94	0,031525
17647373	*Per1*	period circadian clock 1	3,9	0,024427
17693487	*Cxcl6*	chemokine (C-X-C motif) ligand 6	3,86	0,027669
17882347	---	---	3,75	0,013714
17787756	*Emp1*	epithelial membrane protein 1	3,68	0,033698
17747788	*Ccnl1*	cyclin L1	3,56	0,008671
17827855	*Dusp6*	dual specificity phosphatase 6	3,55	0,018941
17807351	*Klf4*	Kruppel-like factor 4 (gut)	3,49	0,020354
17725883	*Spry4*	sprouty RTK signaling antagonist 4	3,48	0,004545
17672779	*Vgf*	VGF nerve growth factor inducible	3,32	0,023502
17833689	---	---	3,29	0,029415

17821997	*Nfkbia*	nuclear factor of kappa light polypeptide gene enhancer in B-cells inhibitor, alpha	3,14	0,001767
17687609	*Atf3*	activating transcription factor 3	3,02	0,021591
17712185	*Rnf122*	ring finger protein 122	2,94	0,028845
17771296	*Slc25a25*	solute carrier family 25 (mitochondrial carrier, phosphate carrier), member 25	2,92	0,016024
17681025	*Rgs16*	regulator of G-protein signaling 16	2,89	0,027214
17806272	*Pnrc1*	proline-rich nuclear receptor coactivator 1	2,87	0,005126
17864319	*Mstn*	myostatin	2,87	0,049177
17738042	*Spry1*	sprouty RTK signaling antagonist 1	2,84	0,031365
17772105	*Rnd3*	Rho family GTPase 3	2,83	0,010714
17808683	*Jun*	jun proto-oncogene	2,78	0,001458
17716849	*Pfkfb3*	6-phosphofructo-2-kinase/fructose-2,6-biphosphatase 3	2,77	0,018456
17696647	*Rel*	v-rel avian reticuloendotheliosis viral oncogene homolog	2,67	0,012933
17809625	*Plk3*	polo-like kinase 3	2,63	0,002178
17723942	*Pmaip1*	phorbol-12-myristate-13-acetate-induced protein 1	2,61	0,026542
17842370	*Icam1*	intercellular adhesion molecule 1	2,6	0,035928
17701753	*Gch1*	GTP cyclohydrolase 1	2,53	0,002142
17855024	---	---	2,51	0,041609
17626511	*Lysmd3*	LysM, putative peptidoglycan-binding, domain containing 3	2,48	0,034801
17624534	*Tmem2*	transmembrane protein 2	2,47	0,035654
17874015	*LOC102552920*	armadillo repeat-containing X-linked protein 5-like	2,45	0,021451
17700983	*Plau*	plasminogen activator, urokinase	2,43	0,014206
17754723	*Arid5b*	AT rich interactive domain 5B (Mrf1 like)	2,35	0,02648
17856543	*Xirp1*	xin actin-binding repeat containing 1	2,35	0,039713
17699345	*Fgf9*	fibroblast growth factor 9	2,33	0,032372
17842577	*Ldlr*	low density lipoprotein receptor	2,31	0,000417
17668004	*Rcan1*	regulator of calcineurin 1	2,27	0,009104
17784192	---	---	2,21	0,037131
17735006	*Lysmd3*	LysM, putative peptidoglycan-binding, domain containing 3	2,21	0,017634
17684906	*Rgs2*	regulator of G-protein signaling 2	2,19	0,012669

17881987	---	---	2,16	0,042304
17669997	*Il1rap*	interleukin 1 receptor accessory protein	2,15	0,029089
17704305	*Slc39a14*	solute carrier family 39 (zinc transporter), member 14	2,14	0,012704
17628839	*Thbs2*	thrombospondin 2	2,13	0,020824
17717253	*Arl5b*	ADP-ribosylation factor-like 5B	2,12	0,041265
17735357	*Gcnt4*	glucosaminyl (N-acetyl) transferase 4, core 2	2,12	0,006454
17858700	*Il1r1*	interleukin 1 receptor, type I	2,11	0,041316
17778973	---	---	2,09	0,030565
17873657	*Zfp711*	zinc finger protein 711	2,08	0,025828
17871271	*Clcn5*	chloride channel, voltage-sensitive 5	2,06	0,020143
17759590	*Arrdc3*	arrestin domain containing 3	2,05	0,012561
17875665	*Timp1*	TIMP metallopeptidase inhibitor 1	2,05	0,01694
17841780	*Gucy1a2*	guanylate cyclase 1, soluble, alpha 2	2,04	0,02076
17821591	*Ahr*	aryl hydrocarbon receptor	2,04	0,020671
17823444	*Alkbh1*	alkB homolog 1, histone H2A dioxygenase	2,02	0,046413
17792855	*Mxd1*	max dimerization protein 1	2,01	0,04203
17701586	*LOC498465*	similar to RIKEN cDNA 1700001F09	2,01	0,011855
17738992	*Tiparp*	TCDD-inducible poly(ADP-ribose) polymerase	2	0,01624
17747976	*Slitrk3*	SLIT and NTRK-like family, member 3	1,98	0,020589
17877765	---	---	1,98	0,017271
17632187	*Plekhf1*	pleckstrin homology domain containing, family F (with FYVE domain) member 1	1,96	0,007662
17806037	*Ripk2*	receptor-interacting serine-threonine kinase 2	1,95	0,010928
17745308	*Snx18*	sorting nexin 18	1,95	0,035326
17781681	*Met*	MET proto-oncogene, receptor tyrosine kinase	1,94	0,015684
17710887	*Jund*	jun D proto-oncogene	1,94	0,01466
17665937	*Stxbp5l*	syntaxin binding protein 5-like	1,93	0,003852
17758328	*Chst3*	carbohydrate (chondroitin 6) sulfotransferase 3	1,93	0,001786
17781511	*Tac1*	tachykinin, precursor 1	1,93	0,012962
17766250	*Nop56*	NOP56 ribonucleoprotein	1,93	0,044318
17881517	---	---	1,93	0,026325
17622730	*Chka*	choline kinase alpha	1,92	0,033161
17740954	---	---	1,92	0,001465
17707458	*LOC100912649*	uncharacterized LOC100912649	1,9	0,039288

17790747	*LOC100909424*	uncharacterized LOC100909424	1,9	0,024031
17721807	*Rnf138*	ring finger protein 138, E3 ubiquitin protein ligase	1,9	0,019447
17687951	*Tmed5*	transmembrane p24 trafficking protein 5	1,89	0,047315
17788437	*Insig1*	insulin induced gene 1	1,87	0,024515
17819669	*Cyp1b1*	cytochrome P450, family 1, subfamily b, polypeptide 1	1,86	0,003685
17718644	*Id4*	inhibitor of DNA binding 4	1,86	0,041135
17783933	*Ppm1k*	protein phosphatase, Mg2+/Mn2+ dependent, 1K	1,85	0,046144
17720423	*Idi1*	isopentenyl-diphosphate delta isomerase 1	1,85	0,009592
17822327	---	---	1,83	0,005046
17682903	*Dusp10*	dual specificity phosphatase 10	1,82	0,033058
17799798	*Dmrta1*	DMRT-like family A1	1,82	0,036453
17680058	*Nuak2*	NUAK family, SNF1-like kinase, 2	1,82	0,036879
17791612	*LOC100365259*	SPT2, Suppressor of Ty, domain containing 1-like	1,81	0,008922
17761562	---	---	1,81	0,032701
17829860	*Dennd3*	DENN/MADD domain containing 3	1,8	0,041285
17694397	*Nsun7*	NOP2/Sun domain family, member 7	1,8	0,02931
17627083	*Ginm1*	glycoprotein integral membrane 1	1,78	0,039753
17822161	*Fbxo33*	F-box protein 33	1,78	0,024646
17793352	*Adamts9*	ADAM metallopeptidase with thrombospondin type 1 motif, 9	1,78	0,001209
17676301	*Mafk*	v-maf avian musculoaponeurotic fibrosarcoma oncogene homolog K	1,78	0,000778
17764350	*Abtb2*	ankyrin repeat and BTB (POZ) domain containing 2	1,78	0,047569
17791993	---	---	1,78	0,042791
17854724	*Trpc1*	transient receptor potential cation channel, subfamily C, member 1	1,77	0,031083
17633894	*Arrdc4*	arrestin domain containing 4	1,77	0,017401
17648462	---	---	1,77	0,00744
17751449	*Sgms2*	sphingomyelin synthase 2	1,76	0,033924
17834490	*LOC102556259*	uncharacterized LOC102556259	1,76	0,03895
17718737	*Cd83*	CD83 molecule	1,75	0,031317
17852141	*Rbm7*	RNA binding motif protein 7	1,75	0,013158
17624893	*Cd274*	CD274 molecule	1,75	0,015925

17685904	*Suco*	SUN domain containing ossification factor	1,75	0,023032
17794626	*Slc2a3*	solute carrier family 2 (facilitated glucose transporter), member 3	1,75	0,012288
17720840	*Fam107b*	family with sequence similarity 107, member B	1,75	0,012546
17686123	*Gorab*	golgin, RAB6-interacting	1,75	0,033846
17710168	---	---	1,74	0,03344
17666544	*P3h2*	prolyl 3-hydroxylase 2	1,73	0,019865
17777661	*Jag1*	jagged 1	1,72	0,016019
17667701	*Grik1*	glutamate receptor, ionotropic, kainate 1	1,71	0,041126
17813490	*Crim1*	cysteine rich transmembrane BMP regulator 1 (chordin like)	1,71	0,041028
17761038	*Ntmt1*	N-terminal Xaa-Pro-Lys N-methyltransferase 1	1,71	0,042081
17701327	*Slc4a7*	solute carrier family 4, sodium bicarbonate cotransporter, member 7	1,71	0,009481
17728989	*Usp38*	ubiquitin specific peptidase 38	1,71	0,026977
17799870	---	---	1,71	0,005118
17705515	*Fgf14*	fibroblast growth factor 14	1,7	0,025604
17801572	*Slc2a1*	solute carrier family 2 (facilitated glucose transporter), member 1	1,7	0,014842
17763597	*Olr469*	olfactory receptor 469	1,7	0,031757
17801318	*Mutyh*	mutY DNA glycosylase	1,69	0,049748
17802767	*Gpr3*	G protein-coupled receptor 3	1,69	0,047659
17815374	*Nampt*	nicotinamide phosphoribosyltransferase	1,68	0,039146
17817771	*Flrt2*	fibronectin leucine rich transmembrane protein 2	1,68	0,005908
17794138	*Csgalnact2*	chondroitin sulfate N-acetylgalactosaminyltransferase 2	1,68	0,0089
17745831	*Dnajc21*	DnaJ (Hsp40) homolog, subfamily C, member 21	1,68	0,002787
17669931	*Fam43a*	family with sequence similarity 43, member A	1,67	0,007102
17755381	*Hs3st5*	heparan sulfate (glucosamine) 3-O-sulfotransferase 5	1,67	0,035607
17728370	*N4bp1*	Nedd4 binding protein 1	1,67	0,025058
17710585	*Tmem38a*	transmembrane protein 38a	1,66	0,047838
17716200	*Crem*	cAMP responsive element modulator	1,66	0,027695
17730047	*Cmtr2*	cap methyltransferase 2	1,66	0,003684
17758749	*Cep85l*	centrosomal protein 85-like	1,66	0,024921
17830875	*Mchr1*	melanin-concentrating hormone receptor 1	1,65	0,040021

17780150	*Gmeb2*	glucocorticoid modulatory element binding protein 2	1,65	0,017065
17726303	*Zfp608*	zinc finger protein 608	1,64	0,034496
17751179	*Ndst3*	N-deacetylase/N-sulfotransferase (heparan glucosaminyl) 3	1,63	0,018381
17694264	*Gabrb1*	gamma-aminobutyric acid (GABA) A receptor, beta 1	1,63	0,048284
17795863	*Gpr19*	G protein-coupled receptor 19	1,63	0,038139
17788148	*Rassf8*	Ras association (RalGDS/AF-6) domain family (N-terminal) member 8	1,63	0,00722
17725716	*Cd14*	CD14 molecule	1,62	0,006605
17767488	*Xkr7*	XK, Kell blood group complex subunit-related family, member 7	1,62	0,013656
17723990	*Tubb6*	tubulin, beta 6 class V	1,62	0,020484
17700455	*Klf5*	Kruppel-like factor 5	1,62	0,044604
17881797	---	---	1,62	0,039118
17841164	*Tfcp2*	transcription factor CP2	1,62	0,008816
17685793	*Rabgap1l*	RAB GTPase activating protein 1-like	1,61	0,04055
17691602	*LOC685655*	transmembrane protein 161B-like	1,61	0,007954
17882589	---	---	1,61	0,002671
17750853	*Gpr88*	G-protein coupled receptor 88	1,6	0,022576
17611128	*Clvs2*	clavesin 2	1,6	0,029352
17745578	*RGD1305938*	similar to expressed sequence AW549877	1,6	0,007008
17671671	*Zfp394*	zinc finger protein 394	1,6	0,014142
17726840	*Mc4r*	melanocortin 4 receptor	1,6	0,00723
17840726	*Ccnt1*	cyclin T1	1,6	0,004032
17680552	*Zfp281*	zinc finger protein 281	1,6	0,039801
17717091	*LOC102547897*	uncharacterized LOC102547897	1,6	0,039641
17655105	---	---	1,59	0,022392
17679878	*Yod1*	YOD1 deubiquitinase	1,59	0,015399
17772842	*Galnt3*	polypeptide N-acetylgalactosaminyltransferase 3	1,59	0,039645
17749555	*Ciart*	circadian associated repressor of transcription	1,59	0,014371
17752186	*Cth*	cystathionine gamma-lyase	1,59	0,015
17627225	*Grm1*	glutamate receptor, metabotropic 1	1,59	0,011867
17865965	*Irs1*	insulin receptor substrate 1	1,58	0,010853
17882197	---	---	1,57	0,049478
17881711	---	---	1,57	0,041701
17745151	*Rab3c*	RAB3C, member RAS oncogene family	1,55	0,039667

17782128	*Strip2*	striatin interacting protein 2	1,55	0,012908
17869792	*RGD1564665*	similar to RIKEN cDNA 4930555G01	1,55	0,045544
17686443	*Uap1*	UDP-N-acetylglucosamine pyrophosphorylase 1	1,55	0,045871
17806476	*B4galt1*	UDP-Gal:betaGlcNAc beta 1,4-galactosyltransferase, polypeptide 1	1,55	0,004714
17680721	*B3galt2*	UDP-Gal:betaGlcNAc beta 1,3-galactosyltransferase, polypeptide 2	1,55	0,044077
17700041	*Htr2a*	5-hydroxytryptamine (serotonin) receptor 2A, G protein-coupled	1,55	0,047805
17729787	*Sntb2*	syntrophin, beta 2	1,55	0,029402
17847470	*Rassf1*	Ras association (RalGDS/AF-6) domain family member 1	1,55	0,032747
17624866	*Jak2*	Janus kinase 2	1,54	0,007132
17804459	*Errfi1*	ERBB receptor feedback inhibitor 1	1,54	0,024585
17859571	---	---	1,54	0,02044
17738018	*Fgf2*	fibroblast growth factor 2	1,54	0,029665
17735224	*Lhfpl2*	lipoma HMGIC fusion partner-like 2	1,53	0,015029
17665637	*Cd200*	Cd200 molecule	1,53	0,038192
17690414	*Hs3st1*	heparan sulfate (glucosamine) 3-O-sulfotransferase 1	1,53	0,011354
17734705	*Lgi1*	leucine-rich, glioma inactivated 1	1,53	0,022471
17741788	*Kcna3*	potassium channel, voltage gated shaker related subfamily A, member 3	1,53	0,044498
17749370	*Pip5k1a*	phosphatidylinositol-4-phosphate 5-kinase, type 1, alpha	1,53	0,019888
17806027	*Osgin2*	oxidative stress induced growth inhibitor family member 2	1,53	0,017188
17774921	*Cd44*	CD44 molecule (Indian blood group)	1,52	0,026808
17815420	*Twistnb*	TWIST neighbor	1,52	0,018365
17827781	*Btg1*	B-cell translocation gene 1, anti-proliferative	1,51	0,0418
17742837	*Slc9b2*	solute carrier family 9, subfamily B (NHA2, cation proton antiporter 2), member 2	1,51	0,022526
17612331	*Chd1*	chromodomain helicase DNA binding protein 1	1,51	0,023452
17660630	*Cwc25*	CWC25 spliceosome-associated protein homolog	1,51	0,014607

17865182	*Erbb4*	erb-b2 receptor tyrosine kinase 4	1,51	0,047705
17846426	---	---	1,51	0,009148
17791666	*Nap1l5*	nucleosome assembly protein 1-like 5	1,5	0,003483
17763815	*Fnbp4*	formin binding protein 4	1,5	0,026209
17823726	*Nrde2*	NRDE-2, necessary for RNA interference, domain containing	1,5	0,026925
17722643	*Pcdhb3*	protocadherin beta 3	1,5	0,004533
17707340	*Zfp868*	zinc finger protein 868	1,5	0,008937
17788314	*RGD1309621*	similar to hypothetical protein FLJ10652	1,49	0,01043
17756290	*RT1-CE4*	RT1 class I, locus CE4	1,49	0,017392
17693612	*Mob1b*	MOB kinase activator 1B	1,49	0,022441
17661537	*Stat3*	signal transducer and activator of transcription 3 (acute-phase response factor)	1,49	0,002107
17728781	*Zswim4*	zinc finger, SWIM-type containing 4	1,49	0,033941
17618230	*Crebzf*	CREB/ATF bZIP transcription factor	1,49	0,030985
17882703	---	---	1,48	0,009894
17881875	---	---	1,48	0,00564
17877863	---	---	1,48	0,006247
17833449	*Matk*	megakaryocyte-associated tyrosine kinase	1,48	0,04607
17845526	*Rora*	RAR-related orphan receptor A	1,48	0,004293
17657405	*Ndel1*	nudE neurodevelopment protein 1-like 1	1,48	0,030163
17787777	---	---	1,48	0,02988
17722380	*Mir1949*	microRNA 1949	1,48	0,006753
17739217	*Fstl5*	follistatin-like 5	1,47	0,01974
17610655	*Olig3*	oligodendrocyte transcription factor 3	1,47	0,03129
17862389	---	---	1,47	0,011733
17717131	*Rpp38*	ribonuclease P/MRP 38 subunit	1,47	0,000244
17706035	---	---	1,47	0,026789
17857136	*Nfya*	nuclear transcription factor-Y alpha	1,47	0,048892
17702453	---	---	1,47	0,018854
17645364	*Olr1401*	olfactory receptor 1401	1,47	0,010726
17816673	*Rhoj*	ras homolog family member J	1,46	0,034417
17735456	*Serf1*	small EDRK-rich factor 1	1,46	0,045426
17655844	*Maml1*	mastermind-like transcriptional coactivator 1	1,46	0,04743
17837576	*LOC690120*	hypothetical protein LOC690120	1,46	0,008899
17759651	*Dusp14*	dual specificity phosphatase 14	1,46	0,037047
17723511	*Chsy3*	chondroitin sulfate synthase 3	1,46	0,035675

17732273	---	---	1,46	0,021192
17854789	*Rasa2*	RAS p21 protein activator 2	1,46	0,044661
17726916	*Ptpn2*	protein tyrosine phosphatase, non-receptor type 2	1,45	0,011425
17716545	*Chrm3*	cholinergic receptor, muscarinic 3	1,45	0,011583
17877652	*Il1rapl1*	interleukin 1 receptor accessory protein-like 1	1,45	0,029001
17742246	*Dpyd*	dihydropyrimidine dehydrogenase	1,45	0,034802
17843512	*Usp2*	ubiquitin specific peptidase 2	1,45	0,045477
17664484	*Bach1*	BTB and CNC homology 1, basic leucine zipper transcription factor 1	1,45	0,031436
17805178	---	---	1,44	0,010546
17781328	---	---	1,44	0,03287
17874778	*Slitrk2*	SLIT and NTRK-like family, member 2	1,44	0,011016
17740832	*Arnt*	aryl hydrocarbon receptor nuclear translocator	1,44	0,032396
17843241	---	---	1,44	0,034307
17805855	---	---	1,44	0,020362
17651986	---	---	1,44	0,005523
17841911	*Mmp8*	matrix metallopeptidase 8	1,43	0,023976
17718650	*Rnf144b*	ring finger protein 144B	1,43	0,03477
17795854	*Dusp16*	dual specificity phosphatase 16	1,43	0,043849
17868421	*LOC103693203*	uncharacterized LOC103693203	1,43	0,039463
17791070	*Olr801*	olfactory receptor 801	1,43	0,046613
17628292	*Ipcef1*	interaction protein for cytohesin exchange factors 1	1,43	0,000184
17810979	---	---	1,43	0,022043
17810981	---	---	1,43	0,022043
17871510	*Acsl4*	acyl-CoA synthetase long-chain family member 4	1,43	0,047452
17730862	*Rpl13*	ribosomal protein L13	1,43	0,001493
17836288	*Mirlet7i*	microRNA let-7i	1,43	0,011357
17746315	*Ralyl*	RALY RNA binding protein-like	1,42	0,00357
17715167	*Eef1e1*	eukaryotic translation elongation factor 1 epsilon 1	1,42	0,019707
17852451	*Zc3h12c*	zinc finger CCCH type containing 12C	1,42	0,025094
17725746	*Taf7*	TAF7 RNA polymerase II, TATA box binding protein (TBP)-associated factor	1,42	0,000814
17631727	*Ffar3*	free fatty acid receptor 3	1,42	0,006153
17722136	*Map3k2*	mitogen activated protein kinase kinase kinase 2	1,42	0,035454
17870238	*LOC102557419*	disks large homolog 5-like	1,41	0,033006

17697770	*Ptger2*	prostaglandin E receptor 2 (subtype EP2)	1,41	0,011162
17697852	*Ptger2*	prostaglandin E receptor 2 (subtype EP2)	1,41	0,011162
17694610	*Rell1*	RELT-like 1	1,41	0,031845
17779567	*B4galt5*	UDP-Gal:betaGlcNAc beta 1,4-galactosyltransferase, polypeptide 5	1,41	0,025218
17831523	*Pim3*	Pim-3 proto-oncogene, serine/threonine kinase	1,41	0,026779
17734956	*RGD1560883*	similar to KIAA0825 protein	1,41	0,022415
17811362	*Zfp593*	zinc finger protein 593	1,41	0,0062
17701193	---	---	1,41	0,013187
17725646	*Nrg2*	neuregulin 2	1,4	0,000591
17788929	*Sema3c*	sema domain, immunoglobulin domain (Ig), short basic domain, secreted, (semaphorin) 3C	1,4	0,0453
17881589	---	---	1,4	0,048757
17866320	---	---	1,4	0,001004
17648900	---	---	1,4	0,043197
17781204	*Steap2*	STEAP family member 2, metalloreductase	1,4	0,006538
17727551	*Rrad*	Ras-related associated with diabetes	1,39	0,038942
17646274	*Hist3h2a*	histone cluster 3, H2a	1,39	0,009747
17815851	*Akap6*	A kinase (PRKA) anchor protein 6	1,39	0,012552
17873917	*Armcx1*	armadillo repeat containing, X-linked 1	1,39	0,047505
17764240	*Lrrc4c*	leucine rich repeat containing 4C	1,39	0,016634
17727831	*Zfp319*	zinc finger protein 319	1,39	0,04005
17791554	*Neurod6*	neuronal differentiation 6	1,39	0,029247
17735693	*Pde4d*	phosphodiesterase 4D, cAMP-specific	1,38	0,019766
17744496	*Hmgcr*	3-hydroxy-3-methylglutaryl-CoA reductase	1,38	0,040084
17669086	*Tmem39a*	transmembrane protein 39a	1,38	0,031592
17719090	*Ripk1*	receptor (TNFRSF)-interacting serine-threonine kinase 1	1,38	0,040681
17717094	*Suv39h2*	suppressor of variegation 3-9 homolog 2 (Drosophila)	1,38	0,044049
17690757	*Tnip2*	TNFAIP3 interacting protein 2	1,37	0,045971
17619355	*Syt9*	synaptotagmin IX	1,37	0,008061
17707399	*Psd3*	pleckstrin and Sec7 domain containing 3	1,37	0,039268
17631334	*Spred3*	sprouty-related, EVH1 domain containing 3	1,37	0,018114
17797426	*Mmp16*	matrix metallopeptidase 16	1,37	0,000178

17745405	---	---	1,37	0,032324
17727484	*Dok6*	docking protein 6	1,37	0,003225
17817822	---	---	1,37	0,019751
17746241	*E2f5*	E2F transcription factor 5	1,37	0,048124
17634009	*Sv2b*	synaptic vesicle glycoprotein 2b	1,36	0,043252
17723147	*Tnfaip8*	tumor necrosis factor, alpha-induced protein 8	1,36	0,00002
17637634	*Fgfr2*	fibroblast growth factor receptor 2	1,36	0,03733
17867395	---	---	1,36	0,006426
17650463	*Phospho1*	phosphatase, orphan 1	1,36	0,018792
17871977	*Wwc3*	WWC family member 3	1,36	0,016105
17736596	*Golph3*	golgi phosphoprotein 3 (coat-protein)	1,36	0,044305
17798703	*Tgfbr1*	transforming growth factor, beta receptor 1	1,36	0,033951
17719969	*Epc1*	enhancer of polycomb homolog 1 (Drosophila)	1,36	0,027787
17791594	*Kbtbd2*	kelch repeat and BTB (POZ) domain containing 2	1,36	0,011347
17806428	*Topors*	topoisomerase I binding, arginine/serine-rich, E3 ubiquitin protein ligase	1,36	0,011344
17882743	---	---	1,35	0,047476
17649532	*Slfn2*	schlafen 2	1,35	0,032063
17736195	*Rictor*	RPTOR independent companion of MTOR, complex 2	1,35	0,000052
17796247	*Sox5*	SRY (sex determining region Y)-box 5	1,35	0,023926
17872514	*RGD1566060*	similar to Magea5	1,35	0,031122
17743101	---	---	1,35	0,03194
17823289	---	---	1,35	0,011827
17664367	---	---	1,35	0,023893
17688026	---	---	1,35	0,049117
17747057	---	---	1,35	0,042475
17821763	*Strn3*	striatin, calmodulin binding protein 3	1,35	0,013509
17655188	*Crebrf*	CREB3 regulatory factor	1,34	0,020357
17845871	*Onecut1*	one cut homeobox 1	1,34	0,042283
17873463	*Atp7a*	ATPase, Cu++ transporting, alpha polypeptide	1,34	0,021429
17616960	---	---	1,34	0,006347
17704837	*Pcdh9*	protocadherin 9	1,34	0,018464
17778114	---	---	1,34	0,023388
17881097	---	---	1,34	0,040259
17714077	*LOC102547665*	spermatogenesis-associated protein 31D1-like	1,34	0,012234
17748262	*Npy2r*	neuropeptide Y receptor Y2	1,34	0,034606

17639313	*Rbm4*	RNA binding motif protein 4	1,34	0,036809
17750733	---	---	1,34	0,017446
17667572	---	---	1,34	0,045716
17806216	*Fut9*	fucosyltransferase 9 (alpha (1,3) fucosyltransferase)	1,33	0,00367
17879988	*Plxna3*	plexin A3	1,33	0,034825
17746334	*Chmp4c*	charged multivesicular body protein 4C	1,33	0,019784
17690416	---	---	1,33	0,003409
17677132	*LOC103691310*	autism susceptibility gene 2 protein-like	1,33	0,035091
17665040	*Epha6*	Eph receptor A6	1,33	0,014253
17776252	*Lcmt2*	leucine carboxyl methyltransferase 2	1,33	0,027987
17882585	---	---	1,33	0,01242
17863125	*Ptchd4*	patched domain containing 4	1,33	0,016231
17648354	*Olr1490*	olfactory receptor 1490	1,33	0,004406
17628094	*Zbtb2*	zinc finger and BTB domain containing 2	1,33	0,015024
17799873	---	---	1,33	0,045582
17869882	*Cbx8*	chromobox homolog 8	1,33	0,031134
17692565	*Rpl5*	ribosomal protein L5	1,33	0,006586
17826838	*Zfp952*	zinc finger protein 952	1,32	0,033388
17874904	*Vma21*	VMA21 vacuolar H+-ATPase homolog (S. cerevisiae)	1,32	0,007224
17769003	*Cd40*	CD40 molecule, TNF receptor superfamily member 5	1,32	0,024638
17868698	---	---	1,32	0,003357
17641268	*Tjp2*	tight junction protein 2	1,32	0,00002
17693015	*Gpat3*	glycerol-3-phosphate acyltransferase 3	1,32	0,015276
17864341	*Nemp2*	nuclear envelope integral membrane protein 2	1,32	0,033415
17785704	---	---	1,32	0,007156
17755956	*Olr1701*	olfactory receptor 1701	1,32	0,000463
17738817	*Npy2r*	neuropeptide Y receptor Y2	1,32	0,040643
17853575	---	---	1,32	0,014317
17854672	---	---	1,32	0,00653
17797218	*Clvs1*	clavesin 1	1,31	0,014434
17746322	*LOC103691503*	RNA-binding Raly-like protein	1,31	0,023214
17722612	*Pcdha4*	protocadherin alpha 4	1,31	0,019917
17701636	*Txndc16*	thioredoxin domain containing 16	1,31	0,009172
17798181	*Dnajb5*	DnaJ (Hsp40) homolog, subfamily B, member 5	1,31	0,042618
17880362	*Kctd12*	potassium channel tetramerization domain containing 12	1,31	0,012963

17627142	*Samd5*	sterile alpha motif domain containing 5	1,31	0,027158
17872486	*Magea8*	melanoma antigen, family A, 8	1,31	0,033758
17750199	*Cttnbp2nl*	CTTNBP2 N-terminal like	1,31	0,00319
17791922	---	---	1,31	0,017722
17839817	*Brd1*	bromodomain containing 1	1,31	0,038412
17720548	*Calml5*	calmodulin-like 5	1,31	0,039317
17646229	*Olr1454*	olfactory receptor 1454	1,31	0,048363
17797653	*Epha7*	Eph receptor A7	1,31	0,029957
17877127	---	---	1,31	0,019981
17734933	*Mctp1*	multiple C2 domains, transmembrane 1	1,3	0,04256
17769874	*Oprl1*	opiate receptor-like 1	1,3	0,040899
17740492	*LOC102552542*	small proline-rich protein 2D-like	1,3	0,046547
17768367	---	---	1,3	0,005712
17865545	*Mir375*	microRNA 375	1,3	0,002759
17781276	*Fzd1*	frizzled class receptor 1	1,3	0,013771
17822331	*Vcpkmt*	valosin containing protein lysine (K) methyltransferase	1,3	0,027195
17668584	---	---	1,3	0,016666
17858023	*Mir30a*	microRNA 30a	1,3	0,040797
17698676	*Bcl2l2*	Bcl2-like 2	1,3	0,036641
17667744	---	---	1,3	0,03326
17715771	*Btn2a2*	butyrophilin, subfamily 2, member A2	1,3	0,020421
17626216	*As3mt*	arsenite methyltransferase	-1,3	0,003804
17844926	*Calml4*	calmodulin-like 4	-1,3	0,00521
17792262	*Rnf181*	ring finger protein 181	-1,3	0,016785
17820764	*Emilin1*	elastin microfibril interfacer 1	-1,3	0,009637
17844994	*Snapc5*	small nuclear RNA activating complex, polypeptide 5	-1,3	0,005204
17617432	*Ttc23*	tetratricopeptide repeat domain 23	-1,3	0,016374
17649300	*Adap2*	ArfGAP with dual PH domains 2	-1,3	0,017674
17808127	*Cdk5rap2*	CDK5 regulatory subunit associated protein 2	-1,3	0,003083
17800600	*Yipf1*	Yip1 domain family, member 1	-1,3	0,031089
17749387	*Scnm1*	sodium channel modifier 1	-1,3	0,027925
17611200	*Hint3*	histidine triad nucleotide binding protein 3	-1,3	0,031873
17764020	*Lrp4*	low density lipoprotein receptor-related protein 4	-1,3	0,024414
17869279	*Uhrf1*	ubiquitin-like with PHD and ring finger domains 1	-1,3	0,012957
17797015	*Mrpl15*	mitochondrial ribosomal protein L15	-1,3	0,017451
17788833	*Gsap*	gamma-secretase activating protein	-1,3	0,019262

17672282	*Pdgfa*	platelet-derived growth factor alpha polypeptide	-1,3	0,040543
17676588	*Gal3st4*	galactose-3-O-sulfotransferase 4	-1,3	0,020079
17617287	*Apba2*	amyloid beta (A4) precursor protein-binding, family A, member 2	-1,3	0,02355
17678138	---	---	-1,3	0,002531
17790889	*Ssbp1*	single-stranded DNA binding protein 1, mitochondrial	-1,3	0,012138
17806407	*Ddx58*	DEAD (Asp-Glu-Ala-Asp) box polypeptide 58	-1,3	0,012406
17690970	*Patz1*	POZ (BTB) and AT hook containing zinc finger 1	-1,3	0,02165
17630326	*Apoc1*	apolipoprotein C-I	-1,3	0,045465
17676874	*Alkbh4*	alkB homolog 4, lysine demthylase	-1,3	0,03294
17633668	*Peg12*	paternally expressed 12	-1,3	0,027363
17858656	*Map4k4*	mitogen-activated protein kinase kinase kinase kinase 4	-1,3	0,026294
17806156	*Pou3f2*	POU class 3 homeobox 2	-1,3	0,010471
17673475	---	---	-1,3	0,017296
17863966	*Tsga10*	testis specific 10	-1,3	0,0029
17839580	*Ttll12*	tubulin tyrosine ligase-like family, member 12	-1,3	0,047056
17711471	*Aadat*	aminoadipate aminotransferase	-1,3	0,014733
17729608	*Pskh1*	protein serine kinase H1	-1,3	0,007867
17646856	---	---	-1,3	0,024021
17618419	*Kctd14*	potassium channel tetramerization domain containing 14	-1,3	0,018878
17877456	*Cldn34e*	claudin 34E	-1,3	0,02895
17676513	*Zfp113*	zinc finger protein 3	-1,31	0,044793
17876142	*RGD1560784*	similar to RIKEN cDNA B630019K06	-1,31	0,02015
17761545	*Fam129b*	family with sequence similarity 129, member B	-1,31	0,035244
17882143	---	---	-1,31	0,035395
17774502	*vb Jhb}Y]v*	nuclear receptor subfamily 1, group H, member 3	-1,31	0,018638
17753066	*Egfl8*	EGF-like-domain, multiple 8	-1,31	0,026177
17708893	*Polb*	polymerase (DNA directed), beta	-1,31	0,015122
17626338	*Gsto1*	glutathione S-transferase omega 1	-1,31	0,013573
17659339	*Pex12*	peroxisomal biogenesis factor 12	-1,31	0,042526
17765014	*Rad51*	RAD51 recombinase	-1,31	0,042056

17711056	*Sugp1*	SURP and G patch domain containing 1	-1,31	0,042876
17880293	*LOC498369*	similar to neighbor of Brca1 gene 1	-1,31	0,040582
17700630	*Mir20a*	microRNA 20a	-1,31	0,029082
17835095	*Apaf1*	apoptotic peptidase activating factor 1	-1,31	0,049054
17785781	*Setmar*	SET domain and mariner transposase fusion gene	-1,31	0,002593
17737454	---	---	-1,31	0,001788
17834131	*Notch3*	notch 3	-1,31	0,008146
17695563	*LOC100910790*	spondin-2-like	-1,31	0,020688
17833278	*Olr1071*	olfactory receptor 1071	-1,31	0,036049
17694709	---	---	-1,31	0,016934
17753579	*Mapk14*	mitogen activated protein kinase 14	-1,31	0,011526
17868170	---	---	-1,31	0,006517
17628915	*Dll1*	delta-like 1 (Drosophila)	-1,31	0,046766
17835750	---	---	-1,31	0,004703
17692928	*Ptpn13*	protein tyrosine phosphatase, non-receptor type 13	-1,32	0,004915
17709537	*Zcchc24*	zinc finger, CCHC domain containing 24	-1,32	0,015556
17806617	*Enho*	energy homeostasis associated	-1,32	0,022708
17793760	*Rad18*	RAD18 E3 ubiquitin protein ligase	-1,32	0,032359
17833435	*Apba3*	amyloid beta (A4) precursor protein-binding, family A, member 3	-1,32	0,002806
17769911	*Pcmtd2*	protein-L-isoaspartate (D-aspartate) O-methyltransferase domain containing 2	-1,32	0,018273
17882393	---	---	-1,32	0,042808
17800675	*RGD1559786*	similar to RIKEN cDNA 0610037L13	-1,32	0,008335
17628406	*Rsph3*	radial spoke 3 homolog (Chlamydomonas)	-1,32	0,005719
17631464	*Zfp82*	zinc finger protein 82	-1,32	0,008146
17870134	*Irx1*	iroquois homeobox 1	-1,32	0,003292
17743018	*Adh1*	alcohol dehydrogenase 1 (class I)	-1,32	0,049465
17820393	*Ehd3*	EH-domain containing 3	-1,32	0,000522
17710092	*Mettl6*	methyltransferase like 6	-1,32	0,036566
17642198	*Cutc*	cutC copper transporter	-1,32	0,004866
17861048	*Armc9*	armadillo repeat containing 9	-1,32	0,014281
17772646	*Psmd14*	proteasome 26S subunit, non-ATPase 14	-1,32	0,017302
17728039	*Hmgxb4*	HMG box domain containing 4	-1,32	0,043808

17830726	*Mief1*	mitochondrial elongation factor 1	-1,32	0,002695
17831297	*Arhgap8*	Rho GTPase activating protein 8	-1,32	0,000852
17677384	*Frg1l1*	FSHD region gene 1-like 1	-1,32	0,046511
17731426	*Gins3*	GINS complex subunit 3 (Psf3 homolog)	-1,32	0,042323
17627529	*Pde7b*	phosphodiesterase 7B	-1,32	0,033795
17612300	*Fam120b*	family with sequence similarity 120B	-1,32	0,03747
17868371	*Fam228b*	family with sequence similarity 228, member B	-1,32	0,039128
17872819	---	---	-1,32	0,004505
17861671	*Olr1349*	olfactory receptor 1349	-1,32	0,037481
17721470	---	---	-1,32	0,011601
17739867	---	---	-1,32	0,015434
17710437	*RGD1562508*	similar to hypothetical protein 4930474N05	-1,32	0,014203
17881429	---	---	-1,33	0,040139
17733638	*Mlkl*	mixed lineage kinase domain-like	-1,33	0,012882
17651355	*Rnd2*	Rho family GTPase 2	-1,33	0,005417
17778476	*E2f1*	E2F transcription factor 1	-1,33	0,021427
17881965	---	---	-1,33	0,031658
17709944	*Nisch*	nischarin	-1,33	0,027794
17834557	*Btbd11*	BTB (POZ) domain containing 11	-1,33	0,014803
17648958	*Poldip2*	polymerase (DNA-directed), delta interacting protein 2	-1,33	0,02137
17638596	*Ifitm10*	interferon induced transmembrane protein 10	-1,33	0,009188
17876839	*Ribc1*	RIB43A domain with coiled-coils 1	-1,33	0,032971
17758871	---	---	-1,33	0,043005
17830086	*Exosc4*	exosome component 4	-1,33	0,046153
17877071	*Ap1s2*	adaptor-related protein complex 1, sigma 2 subunit	-1,33	0,016749
17868119	---	---	-1,33	0,000696
17772367	---	---	-1,33	0,024333
17813676	*Mta3*	metastasis associated 1 family, member 3	-1,34	0,041666
17856061	*Mlh1*	mutL homolog 1, colon cancer, nonpolyposis type 2 (E. coli)	-1,34	0,004581
17726352	*LOC100174910*	glutaredoxin-like protein	-1,34	0,00128
17625860	*Scd2*	stearoyl-Coenzyme A desaturase 2	-1,34	0,000442
17811083	*Gpn2*	GPN-loop GTPase 2	-1,34	0,048084
17643844	*Cdip1*	cell death-inducing p53 target 1	-1,34	0,001689

17831286	*Prr5*	proline rich 5 (renal)	-1,34	0,033036
17875077	*Abcd1*	ATP-binding cassette, subfamily D (ALD), member 1	-1,34	0,024919
17757850	*Cabin1*	calcineurin binding protein 1	-1,34	0,016133
17881813	---	---	-1,34	0,019869
17707040	*Mast3*	microtubule associated serine/threonine kinase 3	-1,34	0,020563
17813896	*Ttc7a*	tetratricopeptide repeat domain 7A	-1,34	0,031394
17817227	---	---	-1,34	0,043916
17796654	*Pex26*	peroxisomal biogenesis factor 26	-1,34	0,000253
17713436	*Qars*	glutaminyl-tRNA synthetase	-1,34	0,016092
17658132	*Vmo1*	vitelline membrane outer layer 1 homolog (chicken)	-1,34	0,034052
17730808	*Acsf3*	acyl-CoA synthetase family member 3	-1,34	0,003806
17796852	*Cpa6*	carboxypeptidase A6	-1,34	0,032579
17618423	*Ints4*	integrator complex subunit 4	-1,34	0,02861
17714337	*H2afy*	H2A histone family, member Y	-1,34	0,026294
17736724	---	---	-1,34	0,024924
17655874	*Rufy1*	RUN and FYVE domain containing 1	-1,35	0,007314
17719126	*Serpinb9*	serpin peptidase inhibitor, clade B (ovalbumin), member 9	-1,35	0,007762
17653172	*Endov*	endonuclease V	-1,35	0,021752
17856353	*Plcd1*	phospholipase C, delta 1	-1,35	0,01623
17855786	*Wdr6*	WD repeat domain 6	-1,35	0,030551
17634078	*Det1*	de-etiolated homolog 1 (Arabidopsis)	-1,35	0,04254
17734486	*Egln1*	egl-9 family hypoxia-inducible factor 1	-1,35	0,034607
17642510	*Ldb1*	LIM domain binding 1	-1,35	0,01774
17646550	*Dhrs7b*	dehydrogenase/reductase (SDR family) member 7B	-1,35	0,01311
17719626	---	---	-1,35	0,014344
17730428	*Bco1*	beta-carotene oxygenase 1	-1,35	0,04
17840528	*Rapgef3*	Rap guanine nucleotide exchange factor (GEF) 3	-1,35	0,014786
17782189	*Cpa2*	carboxypeptidase A2 (pancreatic)	-1,35	0,001545
17883073	---	---	-1,35	0,042709
17788697	*LOC100364673*	hypercoagulability-related protein-like	-1,35	0,006684
17627340	*Phactr2*	phosphatase and actin regulator 2	-1,36	0,033568
17677356	*Glt1d1*	glycosyltransferase 1 domain containing 1	-1,36	0,044194
17644606	*Tekt4*	tektin 4	-1,36	0,020942

17677600	*Snrnp35*	small nuclear ribonucleoprotein 35 (U11/U12)	-1,36	0,04672
17790962	---	---	-1,36	0,040227
17667257	*Ppil2*	peptidylprolyl isomerase (cyclophilin)-like 2	-1,36	0,049272
17783495	*Malsu1*	mitochondrial assembly of ribosomal large subunit 1	-1,36	0,036011
17797235	*MGC94199*	similar to RIKEN cDNA 2610301B20; EST AI428449	-1,36	0,04484
17737008	*LOC100360380*	zinc finger protein 457-like	-1,36	0,027981
17834292	*Cyp4f1*	cytochrome P450, family 4, subfamily f, polypeptide 1	-1,36	0,035638
17855584	*Slc38a3*	solute carrier family 38, member 3	-1,36	0,049689
17755414	*LOC102551702*	uncharacterized LOC102551702 [Source:RGD Symbol;Acc:7497946]	-1,36	0,033763
17847682	*Qars*	glutaminyl-tRNA synthetase	-1,36	0,044966
17675429	*LOC100360453*	chromobox homolog 3-like	-1,36	0,045027
17623100	*Eif1ad*	eukaryotic translation initiation factor 1A domain containing	-1,36	0,000588
17710308	*LOC100910026*	uncharacterized LOC100910026	-1,36	0,026997
17843057	*Pate2*	prostate and testis expressed 2	-1,36	0,030542
17883075	---	---	-1,36	0,036251
17740402	*S100a16*	S100 calcium binding protein A16	-1,36	0,044936
17716330	---	---	-1,36	0,02006
17769857	---	---	-1,36	0,039389
17834443	---	---	-1,36	0,019218
17830353	*Apol3*	apolipoprotein L, 3	-1,37	0,021416
17665449	*Dzip3*	DAZ interacting zinc finger protein 3	-1,37	0,011347
17695602	*Ywhah*	tyrosine 3-monooxygenase/tryptophan 5-monooxygenase activation protein, eta	-1,37	0,046478
17718501	*Iars*	isoleucyl-tRNA synthetase	-1,37	0,013718
17880973	*Safb2*	scaffold attachment factor B2	-1,37	0,033894
17674425	*Sirt4*	sirtuin 4	-1,37	0,033866
17801282	*Prdx1*	peroxiredoxin 1	-1,37	0,038537
17749608	*Bola1*	bolA family member 1	-1,37	0,008839
17872106	*Egfl6*	EGF-like-domain, multiple 6	-1,37	0,031908
17675431	*LOC102548389*	zinc finger protein 709-like	-1,37	0,023927
17759295	*Cep57l1*	centrosomal protein 57-like 1	-1,37	0,011237

17838576	*Sharpin*	SHANK-associated RH domain interactor	-1,37	0,045139
17629355	*Aurkc*	aurora kinase C	-1,38	0,042775
17655773	*Irgm*	immunity-related GTPase family, M	-1,38	0,025575
17818613	---	---	-1,38	0,049144
17794610	*Apobec1*	apolipoprotein B mRNA editing enzyme, catalytic polypeptide 1	-1,38	0,025441
17743777	---	---	-1,38	0,027589
17850971	*Chek1*	checkpoint kinase 1	-1,38	0,025292
17882395	---	---	-1,38	0,034174
17861900	*D2hgdh*	D-2-hydroxyglutarate dehydrogenase	-1,38	0,048603
17770675	*Notch1*	notch 1	-1,38	0,013296
17779395	*Zfp663*	zinc finger protein 663	-1,38	0,009835
17705829	*Il17rd*	interleukin 17 receptor D	-1,38	0,015255
17633477	*LOC102551282*	uncharacterized LOC102551282	-1,38	0,03153
17770205	*Ndor1*	NADPH dependent diflavin oxidoreductase 1	-1,38	0,022158
17670701	*Klhl22*	kelch-like family member 22	-1,38	0,005651
17730703	*Banp*	Btg3 associated nuclear protein	-1,38	0,03631
17726833	---	---	-1,38	0,038253
17654006	*Cluap1*	clusterin associated protein 1	-1,38	0,043964
17869930	*Sctr*	secretin receptor	-1,38	0,049533
17820444	*Fam179a*	family with sequence similarity 179, member A	-1,39	0,03165
17691150	*Gatsl3*	GATS protein-like 3	-1,39	0,00048
17777032	*LOC100909998*	zinc finger protein 2-like	-1,39	0,014245
17800346	*Oma1*	OMA1 zinc metallopeptidase	-1,39	0,003583
17818697	*Mir134*	microRNA 134	-1,39	0,001321
17825446	*Prim1*	primase, DNA, polypeptide 1	-1,39	0,040357
17817981	*Golga5*	golgin A5	-1,39	0,016026
17674119	*Adam1a*	a disintegrin and metallopeptidase domain 1a	-1,39	0,022508
17670816	*Snap29*	synaptosomal-associated protein 29	-1,39	0,015117
17721926	*Mocos*	molybdenum cofactor sulfurase	-1,39	0,023544
17798486	*Trmt10b*	tRNA methyltransferase 10B	-1,39	0,032614
17826580	*Rnf126*	ring finger protein 126	-1,39	0,041381
17820353	*Slc30a6*	solute carrier family 30 (zinc transporter), member 6	-1,39	0,033856
17613001	*Zfp865*	zinc finger protein 865	-1,39	0,031335
17637458	*Zfp689*	zinc finger protein 689	-1,39	0,044007
17866309	*Hjurp*	Holliday junction recognition protein	-1,39	0,007571
17823045	---	---	-1,39	0,032983

17881545	---	---	-1,39	0,005587
17639089	*Gstp1*	glutathione S-transferase pi 1	-1,39	0,00678
17820653	---	---	-1,39	0,029331
17712930	*Tfdp1*	transcription factor Dp-1	-1,39	0,030931
17770874	*Vav2*	vav 2 guanine nucleotide exchange factor	-1,4	0,030259
17706231	*Btd*	biotinidase	-1,4	0,048209
17658462	*Aspa*	aspartoacylase	-1,4	0,007047
17754172	*Pfkl*	phosphofructokinase, liver	-1,4	0,016174
17692728	*Lrrc8d*	leucine rich repeat containing 8 family, member D	-1,4	0,030861
17674290	*Rnft2*	ring finger protein, transmembrane 2	-1,4	0,025366
17848991	*Pomgnt2*	protein O-linked mannose N-acetylglucosaminyltransferase 2 (beta 1,4-)	-1,4	0,012976
17637841	*Dhx32*	DEAH (Asp-Glu-Ala-His) box polypeptide 32	-1,4	0,000444
17845161	*Parp16*	poly (ADP-ribose) polymerase family, member 16	-1,4	0,038336
17646671	*Pigl*	phosphatidylinositol glycan anchor biosynthesis, class L	-1,4	0,017381
17746806	*Mrpl47*	mitochondrial ribosomal protein L47	-1,4	0,026888
17671997	*Radil*	Ras association and DIL domains	-1,4	0,033454
17733006	*B3gnt9*	UDP-GlcNAc:betaGal beta-1,3-N-acetylglucosaminyltransferase 9	-1,4	0,003945
17623809	*Ints5*	integrator complex subunit 5	-1,4	0,034068
17718176	*Nsd1*	nuclear receptor binding SET domain protein 1	-1,4	0,042495
17724592	*Zadh2*	zinc binding alcohol dehydrogenase, domain containing 2	-1,4	0,040458
17711779	*Ccdc110*	coiled-coil domain containing 110	-1,4	0,024563
17868137	---	---	-1,4	0,028903
17616136	*Akt1s1*	AKT1 substrate 1 (proline-rich)	-1,4	0,00442
17656106	*Rad50*	RAD50 homolog, double strand break repair protein	-1,4	0,010363
17754023	*Pknox1*	PBX/knotted 1 homeobox 1	-1,4	0,049949
17669839	*Wdr53*	WD repeat domain 53	-1,4	0,030441
17699695	*Ccdc25*	coiled-coil domain containing 25	-1,4	0,026991
17814753	*Rdh14*	retinol dehydrogenase 14 (all-trans/9-cis/11-cis)	-1,4	0,022315
17754929	*Ascc1*	activating signal cointegrator 1 complex subunit 1	-1,4	0,009469

17852970	*Csk*	c-src tyrosine kinase	-1,41	0,01079
17758498	*H2afy2*	H2A histone family, member Y2	-1,41	0,038978
17758233	*Tet1*	tet methylcytosine dioxygenase 1	-1,41	0,022337
17744679	*Ptcd2*	pentatricopeptide repeat domain 2	-1,41	0,038893
17631487	*Lrfn3*	leucine rich repeat and fibronectin type III domain containing 3	-1,41	0,026147
17678896	*Ficd*	FIC domain containing	-1,41	0,018031
17806347	*Smim8*	small integral membrane protein 8	-1,41	0,003986
17678658	*Msi1*	musashi RNA-binding protein 1	-1,41	0,019678
17881845	---	---	-1,41	0,013641
17870180	---	---	-1,41	0,042545
17669104	*Popdc2*	popeye domain containing 2	-1,41	0,048592
17737173	*Zfand1*	zinc finger, AN1-type domain 1	-1,41	0,007074
17639867	*Sac3d1*	SAC3 domain containing 1	-1,41	0,002924
17620967	*Orai3*	ORAI calcium release-activated calcium modulator 3	-1,41	0,027393
17629549	*Zfp524*	zinc finger protein 524	-1,41	0,032026
17814206	*Ppm1g*	protein phosphatase, Mg2+/Mn2+ dependent, 1G	-1,41	0,012143
17780691	---	---	-1,41	0,020517
17688114	*LOC685989*	hypothetical protein LOC685989	-1,41	0,031754
17727939	*Fam192a*	family with sequence similarity 192, member A	-1,41	0,027036
17800058	*Efcab7*	EF-hand calcium binding domain 7	-1,41	0,004935
17869946	---	---	-1,41	0,003583
17673942	*Ift81*	intraflagellar transport 81	-1,41	0,029115
17723355	*Megf10*	multiple EGF-like domains 10	-1,42	0,016683
17681144	*Sec16b*	SEC16 homolog B, endoplasmic reticulum export factor	-1,42	0,047617
17883067	---	---	-1,42	0,045706
17793809	*Jagn1*	jagunal homolog 1	-1,42	0,015431
17870670	*Klhl13*	kelch-like family member 13	-1,42	0,037597
17720784	*Phyh*	phytanoyl-CoA 2-hydroxylase	-1,42	0,007127
17843475	*Lnc215*	long non-coding RNA 215	-1,42	0,021552
17699369	*Cab39l*	calcium binding protein 39-like	-1,42	0,031353
17678502	---	---	-1,42	0,012144
17777877	*Rbbp9*	retinoblastoma binding protein 9	-1,42	0,039099
17744363	*Aggf1*	angiogenic factor with G patch and FHA domains 1	-1,42	0,016524
17867516	---	---	-1,42	0,005021
17823707	*Foxn3*	forkhead box N3	-1,43	0,022648

17784334	*Eif2ak3*	eukaryotic translation initiation factor 2 alpha kinase 3	-1,43	0,010577
17801650	*Zmynd12*	zinc finger, MYND-type containing 12	-1,43	0,029906
17817470	*LOC685221*	hypothetical protein LOC685221 [Source:RGD Symbol;Acc:1596475]	-1,43	0,023931
17618818	*Arap1*	ArfGAP with RhoGAP domain, ankyrin repeat and PH domain 1	-1,43	0,005844
17770774	*Med22*	mediator complex subunit 22	-1,43	0,007801
17614681	*Blvrb*	biliverdin reductase B	-1,43	0,034797
17824388	*Cinp*	cyclin-dependent kinase 2-interacting protein	-1,43	0,018375
17727183	*Haus1*	HAUS augmin-like complex, subunit 1	-1,43	0,012741
17835825	*Tmem19*	transmembrane protein 19	-1,43	0,012477
17700015	*Med4*	mediator complex subunit 4	-1,43	0,001946
17795195	*Rhno1*	RAD9-HUS1-RAD1 interacting nuclear orphan 1	-1,43	0,042455
17838798	*Lrrc24*	leucine rich repeat containing 24	-1,44	0,039208
17762760	*Erich2*	glutamate-rich 2	-1,44	0,005462
17756130	*Rps2-ps1*	ribosomal protein S2, pseudogene 1	-1,44	0,011752
17813311	*Cptp*	ceramide-1-phosphate transfer protein	-1,44	0,046683
17876745	*Foxr2*	forkhead box R2	-1,44	0,034086
17658384	*Tekt1*	tektin 1	-1,44	0,026906
17743578	*Slc44a5*	solute carrier family 44, member 5	-1,44	0,026511
17731432	*Ccdc113*	coiled-coil domain containing 113	-1,44	0,043286
17656149	*Slc22a5*	solute carrier family 22 (organic cation/carnitine transporter), member 5	-1,44	0,030463
17833606	*Sf3a2*	splicing factor 3a, subunit 2	-1,44	0,03342
17678915	*Mn1*	meningioma 1	-1,44	0,039968
17782681	*Tas2r108*	taste receptor, type 2, member 108	-1,44	0,002872
17844818	---	---	-1,44	0,018487
17725099	*Klhl14*	kelch-like family member 14	-1,44	0,042518
17624427	*Nmrk1*	nicotinamide riboside kinase 1	-1,44	0,023243
17722313	*Kif20a*	kinesin family member 20A	-1,45	0,00943
17705225	*Abcc4*	ATP-binding cassette, subfamily C (CFTR/MRP), member 4	-1,45	0,024637
17703490	*Cdadc1*	cytidine and dCMP deaminase domain containing 1	-1,45	0,030727

17683672	*Tmem177*	transmembrane protein 177	-1,45	0,045249
17777484	*Rassf2*	Ras association (RalGDS/AF-6) domain family member 2	-1,45	0,017161
17834057	*Tpgs1*	tubulin polyglutamylase complex subunit 1	-1,45	0,00578
17830336	*Commd5*	COMM domain containing 5	-1,45	0,034456
17776077	*Zfp106*	zinc finger protein 106	-1,45	0,00521
17721894	*MGC116121*	similar to RIKEN cDNA 2700062C07	-1,46	0,031373
17848680	*Ctdspl*	CTD (carboxy-terminal domain, RNA polymerase II, polypeptide A) small phosphatase-like	-1,46	0,001619
17844649	*RGD1562618*	similar to RIKEN cDNA 6030419C18 gene	-1,46	0,014806
17809123	*Tceanc2*	transcription elongation factor A (SII) N-terminal and central domain containing 2	-1,46	0,011851
17656698	*Snap47*	synaptosomal-associated protein, 47	-1,46	0,022642
17780480	*Wdr86*	WD repeat domain 86	-1,46	0,039971
17818441	---	---	-1,46	0,007415
17818705	*Mir496*	microRNA 496	-1,46	0,026896
17783384	*Zfp775*	zinc finger protein 775	-1,46	0,008015
17684221	*Sox13*	SRY box 13	-1,46	0,01742
17818103	*Ppp4r4*	protein phosphatase 4, regulatory subunit 4	-1,46	0,009326
17762996	*Cdca7*	cell division cycle associated 7	-1,46	0,021707
17697477	---	---	-1,46	0,02858
17770484	*Ubac1*	UBA domain containing 1	-1,47	0,023713
17881919	---	---	-1,47	0,029332
17671379	---	---	-1,47	0,026248
17666640	*Tbccd1*	TBCC domain containing 1	-1,47	0,029968
17616470	*Dbp*	D site of albumin promoter (albumin D-box) binding protein	-1,47	0,029446
17674349	*Suds3*	SDS3 homolog, SIN3A corepressor complex component	-1,47	0,030206
17873337	*Pin4*	peptidylprolyl cis/trans isomerase, NIMA-interacting 4	-1,47	0,041346
17734042	*Fbxo31*	F-box protein 31	-1,47	0,031137
17806595	*RGD1309821*	similar to KIAA1161 protein	-1,47	0,049553
17883149	---	---	-1,48	0,007815
17832006	*Slc48a1*	solute carrier family 48 (heme transporter), member 1	-1,48	0,013044
17619705	---	---	-1,48	0,049932
17618305	---	---	-1,48	0,036425
17800514	*Pars2*	prolyl-tRNA synthetase 2, mitochondrial (putative)	-1,48	0,00632

17880404	*Sox4*	SRY box 4	-1,48	0,020371
17843304	*Olr1244*	olfactory receptor 1244	-1,48	0,006418
17636027	*Lmo1*	LIM domain only 1	-1,48	0,003289
17870189	*LOC685989*	hypothetical protein LOC685989	-1,48	0,039919
17745897	*Tars*	threonyl-tRNA synthetase	-1,48	0,029442
17843026	*Cdon*	cell adhesion associated, oncogene regulated	-1,49	0,006458
17789472	*LOC103692066*	sterile alpha motif domain-containing protein 9-like	-1,49	0,048185
17809656	*Kif2c*	kinesin family member 2C	-1,49	0,000006
17638756	*Osbpl5*	oxysterol binding protein-like 5	-1,49	0,007756
17785174	*RGD1306746*	similar to Hypothetical protein MGC25529	-1,49	0,028269
17768463	*Fam83d*	family with sequence similarity 83, member D	-1,49	0,000296
17643588	*Dexi*	dexamethasone-induced transcript	-1,49	0,030129
17639779	---	---	-1,49	0,041741
17677506	*Atp6v0a2*	ATPase, H+ transporting, lysosomal V0 subunit A2 [Source:RGD Symbol;Acc:621006]	-1,49	0,033491
17883045	---	---	-1,49	0,035385
17661411	*Dnajc7*	DnaJ (Hsp40) homolog, subfamily C, member 7	-1,49	0,004776
17620909	*Tmem265*	transmembrane protein 265	-1,49	0,004856
17839000	*Tst*	thiosulfate sulfurtransferase	-1,49	0,005561
17814737	*Osr1*	odd-skipped related transciption factor 1	-1,5	0,016389
17652831	*Sap30bp*	SAP30 binding protein	-1,5	0,00289
17663243	*Mif4gd*	MIF4G domain containing	-1,5	0,006137
17878870	*Zmat1*	zinc finger, matrin-type 1	-1,5	0,027131
17690828	*Nelfa*	negative elongation factor complex member A	-1,5	0,044722
17630461	*LOC102548695*	zinc finger protein 45-like	-1,5	0,03275
17825596	*Stat2*	signal transducer and activator of transcription 2	-1,5	0,03033
17749048	*Ints3*	integrator complex subunit 3	-1,5	0,011052
17790301	*Tmem209*	transmembrane protein 209	-1,5	0,041725
17633455	---	---	-1,5	0,030621
17742533	---	---	-1,5	0,037912
17755165	*Asf1a*	anti-silencing function 1A histone chaperone	-1,5	0,00941
17713789	*Slc35d2*	solute carrier family 35 (UDP-GlcNAc/UDP-glucose transporter), member D2	-1,51	0,001563

17745749	*Slc1a3*	solute carrier family 1 (glial high affinity glutamate transporter), member 3	-1,51	0,019371
17818675	*Mir667*	microRNA 667	-1,51	0,015349
17655423	*Wwc1*	WW and C2 domain containing 1	-1,51	0,046289
17882843	---	---	-1,51	0,03478
17734766	*Rmnd5b*	required for meiotic nuclear division 5 homolog B (S. cerevisiae)	-1,51	0,00534
17722537	*Slc35a4*	solute carrier family 35, member A4	-1,52	0,032665
17612922	*Zim1*	zinc finger, imprinted 1	-1,52	0,033346
17869659	*LOC685989*	hypothetical protein LOC685989	-1,52	0,011439
17865806	*Farsb*	phenylalanyl-tRNA synthetase, beta subunit	-1,52	0,022082
17780694	---	---	-1,52	0,0074
17882717	---	---	-1,52	0,011047
17852378	*Layn*	layilin	-1,53	0,007501
17882075	---	---	-1,53	0,018611
17778813	*Dsn1*	DSN1 homolog, MIS12 kinetochore complex component	-1,53	0,028875
17848969	*Trak1*	trafficking protein, kinesin binding 1	-1,53	0,04518
17780561	*Tmub1*	transmembrane and ubiquitin-like domain containing 1	-1,53	0,028944
17713373	*Ccdc71*	coiled-coil domain containing 71	-1,53	0,047645
17789479	*LOC102553788*	uncharacterized LOC102553788	-1,53	0,028896
17867699	*LOC679711*	similar to RIKEN cDNA 5031410I06	-1,53	0,005237
17670745	*P2rx6*	purinergic receptor P2X, ligand-gated ion channel, 6	-1,53	0,000256
17743078	*Adh5*	alcohol dehydrogenase 5 (class III), chi polypeptide	-1,53	0,029301
17678752	*Ankrd13a*	ankyrin repeat domain 13a	-1,53	0,041833
17696845	*LOC102552540*	uncharacterized LOC102552540	-1,53	0,022493
17753780	*Zfand3*	zinc finger, AN1-type domain 3	-1,54	0,007001
17662657	*Cacng5*	calcium channel, voltage-dependent, gamma subunit 5	-1,54	0,035554
17801480	*Slc6a9*	solute carrier family 6 (neurotransmitter transporter, glycine), member 9	-1,54	0,028141
17869418	*LOC103690177*	uncharacterized LOC103690177	-1,54	0,026891

17848911	*Myrip*	myosin VIIA and Rab interacting protein	-1,54	0,031348
17651899	---	---	-1,54	0,009958
17791437	*Tril*	TLR4 interactor with leucine-rich repeats	-1,54	0,016182
17635371	*Inppl1*	inositol polyphosphate phosphatase-like 1	-1,55	0,012003
17880740	---	---	-1,55	0,044644
17715250	*Rpp40*	ribonuclease P/MRP 40 subunit	-1,55	0,010866
17726556	*Pcyox1l*	prenylcysteine oxidase 1 like	-1,55	0,002412
17667043	*Prodh*	proline dehydrogenase (oxidase) 1	-1,55	0,029768
17869593	---	---	-1,55	0,002123
17704681	*Fam216b*	family with sequence similarity 216, member B	-1,56	0,020628
17777051	*Nphp1*	nephronophthisis 1 (juvenile)	-1,56	0,028261
17828565	*Cdk4*	cyclin-dependent kinase 4	-1,56	0,015809
17875937	*Nyx*	nyctalopin	-1,56	0,015478
17726109	*LOC100359715*	NADH dehydrogenase (ubiquinone) 1 beta subcomplex 3-like [Source:RGD Symbol;Acc:2323525]	-1,56	0,033988
17854693	*Chst2*	carbohydrate (N-acetylglucosamine-6-O) sulfotransferase 2	-1,57	0,031479
17737004	*LOC100913005*	zinc finger protein 99-like	-1,57	0,01265
17817851	*Tdp1*	tyrosyl-DNA phosphodiesterase 1	-1,57	0,013877
17703332	*N6amt2*	N-6 adenine-specific DNA methyltransferase 2 (putative)	-1,57	0,045165
17766807	*Mgme1*	mitochondrial genome maintenance exonuclease 1	-1,57	0,01638
17676705	*Pop7*	POP7 homolog, ribonuclease P/MRP subunit	-1,57	0,031573
17868281	*LOC501346*	hypothetical LOC501346	-1,57	0,04345
17769238	*Dok5*	docking protein 5	-1,58	0,008342
17881120	---	---	-1,58	0,010893
17671866	*Zdhhc4*	zinc finger, DHHC-type containing 4	-1,58	0,032465
17827179	*Mterf2*	mitochondrial transcription termination factor 2	-1,58	0,049429
17780805	*Dnajc2*	DnaJ (Hsp40) homolog, subfamily C, member 2	-1,58	0,000273
17810932	*Zcchc17*	zinc finger, CCHC domain containing 17	-1,58	0,044348
17708921	*Smim19*	small integral membrane protein 19	-1,58	0,011057
17782382	*Agbl3*	ATP/GTP binding protein-like 3	-1,58	0,037992

17612473	*LOC103691005*	zinc finger protein 679-like	-1,59	0,036747
17825947	---	---	-1,59	0,046743
17702753	*Homez*	homeobox and leucine zipper encoding	-1,59	0,034986
17882369	---	---	-1,6	0,030009
17633748	*Tjp1*	tight junction protein 1	-1,6	0,003826
17697020	*Camk2g*	calcium/calmodulin-dependent protein kinase II gamma	-1,6	0,047666
17703574	*Tnfrsf19*	tumor necrosis factor receptor superfamily, member 19	-1,6	0,032493
17806600	*RGD1561916*	similar to testes development-related NYD-SP22 isoform 1	-1,6	0,046262
17827186	*Fhl4*	four and a half LIM domains 4	-1,6	0,029334
17637570	*LOC103691238*	zinc finger protein 239-like	-1,61	0,033567
17715267	*Eci2*	enoyl-CoA delta isomerase 2	-1,61	0,020472
17856909	*Sgol1*	shugoshin-like 1 (S. pombe)	-1,61	0,045952
17762924	*Pdk1*	pyruvate dehydrogenase kinase, isozyme 1	-1,61	0,023832
17711284	---	---	-1,61	0,003088
17734475	*RGD1562218*	similar to RIKEN cDNA 0610039J04	-1,62	0,029198
17748863	*Efna4*	ephrin A4	-1,62	0,003247
17636674	---	---	-1,62	0,015057
17864955	*Wdr12*	WD repeat domain 12	-1,62	0,018035
17747605	*P2ry12*	purinergic receptor P2Y, G-protein coupled, 12	-1,63	0,040903
17618760	*Coa4*	cytochrome c oxidase assembly factor 4 homolog	-1,63	0,028491
17696484	*Cep68*	centrosomal protein 68	-1,63	0,009305
17883037	---	---	-1,63	0,009869
17883041	---	---	-1,63	0,009869
17677613	*RGD1563482*	similar to hypothetical protein FLJ38663	-1,63	0,017086
17696692	*Vrk2*	vaccinia related kinase 2	-1,64	0,020185
17701494	*Abhd6*	abhydrolase domain containing 6	-1,64	0,006592
17784767	*Rtkn*	rhotekin	-1,64	0,026416
17746941	*Ccna2*	cyclin A2	-1,64	0,036447
17756036	*Znrd1as1*	ZNRD1 antisense RNA 1	-1,64	0,01476
17838118	---	---	-1,64	0,018292
17883077	---	---	-1,64	0,023407
17612377	---	---	-1,64	0,008948
17873593	*Pou3f4*	POU class 3 homeobox 4	-1,65	0,026503
17851230	*Olr1253*	olfactory receptor 1253	-1,65	0,038475
17832535	*Tns2*	tensin 2	-1,66	0,023211
17624043	*Cyb561a3*	cytochrome b561 family, member A3	-1,66	0,014284

17811774	*Ubxn10*	UBX domain protein 10	-1,66	0,034084
17882021	---	---	-1,66	0,032882
17688122	*LOC501297*	hypothetical LOC501297	-1,66	0,011644
17697472	*Oxsm*	3-oxoacyl-ACP synthase, mitochondrial	-1,66	0,031923
17633732	---	---	-1,66	0,016797
17883091	---	---	-1,66	0,030711
17623055	*Cd248*	CD248 molecule, endosialin	-1,67	0,00498
17883051	---	---	-1,67	0,005195
17883087	---	---	-1,67	0,005195
17818411	---	---	-1,68	0,000111
17684321	*Adora1*	adenosine A1 receptor	-1,68	0,035522
17869387	*LOC103690177*	uncharacterized LOC103690177	-1,68	0,029793
17673079	*Limk1*	LIM domain kinase 1	-1,69	0,035072
17744118	*Atg10*	autophagy related 10	-1,69	0,040248
17698701	---	---	-1,69	0,030604
17869685	---	---	-1,7	0,005798
17667929	*Dnajc28*	DnaJ (Hsp40) homolog, subfamily C, member 28	-1,7	0,040255
17703995	*Cdca2*	cell division cycle associated 2	-1,7	0,008674
17645602	*Clk4*	CDC-like kinase 4	-1,7	0,034168
17817308	*Acot1*	acyl-CoA thioesterase 1	-1,7	0,026905
17771687	*Ttll11*	tubulin tyrosine ligase-like family, member 11	-1,71	0,027283
17802251	*Dlgap3*	discs, large (Drosophila) homolog-associated protein 3	-1,71	0,029827
17697685	---	---	-1,72	0,049138
17654808	*Sox8*	SRY (sex determining region Y)-box 8	-1,72	0,037226
17803550	*Akr7a2*	aldo-keto reductase family 7, member A2	-1,72	0,045788
17883081	---	---	-1,72	0,030942
17867695	---	---	-1,72	0,01941
17819200	*RGD1307315*	LOC362793	-1,73	0,009861
17620368	*Plk1*	polo-like kinase 1	-1,73	0,004776
17860426	*Plcd4*	phospholipase C, delta 4	-1,73	0,041372
17850757	*St14*	suppression of tumorigenicity 14 (colon carcinoma)	-1,74	0,044168
17850354	*Kank2*	KN motif and ankyrin repeat domains 2	-1,74	0,001505
17753738	*Rnf8*	ring finger protein 8, E3 ubiquitin protein ligase	-1,74	0,033579
17764385	*Cstf3*	cleavage stimulation factor, 3 pre-RNA, subunit 3, 77kDa	-1,74	0,031749
17759845	*Tmem203*	transmembrane protein 203	-1,74	0,032282
17792068	---	---	-1,74	0,012284
17863382	*Gsta1*	glutathione S-transferase alpha 1	-1,75	0,00224

17717679	*RGD1566325*	similar to regulator of sex-limitation candidate 16	-1,75	0,013246
17677508	*Atp6v0a2*	ATPase, H+ transporting, lysosomal V0 subunit A2	-1,76	0,043791
17818715	*Mir369*	microRNA 369	-1,76	0,039587
17883131	---	---	-1,77	0,021999
17869956	*LOC679711*	similar to RIKEN cDNA 5031410I06	-1,77	0,000719
17635675	*Trim5*	tripartite motif-containing 5	-1,77	0,010681
17831179	*Mcat*	malonyl CoA:ACP acyltransferase (mitochondrial)	-1,77	0,000172
17868025	*LOC102552540*	uncharacterized LOC102552540	-1,77	0,014609
17868103	*LOC679711*	similar to RIKEN cDNA 5031410I06	-1,78	0,041603
17636900	*Palb2*	partner and localizer of BRCA2	-1,78	0,037886
17853194	*Senp8*	SUMO/sentrin peptidase family member, NEDD8 specific	-1,79	0,024414
17800968	*Spata6*	spermatogenesis associated 6	-1,79	0,013101
17717692	*RGD1566325*	similar to regulator of sex-limitation candidate 16	-1,79	0,02741
17677198	*Tpst1*	tyrosylprotein sulfotransferase 1	-1,79	0,009487
17870827	*Ndp*	Norrie disease (pseudoglioma)	-1,8	0,010512
17818443	*LOC102555254*	uncharacterized LOC102555254	-1,81	0,019766
17868175	*LOC363301*	hypothetical LOC363301	-1,81	0,035266
17868106	---	---	-1,81	0,019476
17737014	---	---	-1,82	0,049348
17804162	*Mad2l2*	MAD2 mitotic arrest deficient-like 2 (yeast)	-1,83	0,045129
17692717	---	---	-1,83	0,026014
17748050	*Golim4*	golgi integral membrane protein 4	-1,84	0,001282
17833710	*Reep6*	receptor accessory protein 6	-1,84	0,025564
17663823	*Lgals3bp*	lectin, galactoside-binding, soluble, 3 binding protein	-1,84	0,037992
17818439	---	---	-1,84	0,048934
17659210	*Tefm*	transcription elongation factor, mitochondrial	-1,84	0,019301
17612464	*Vom1r-ps112*	vomeronasal 1 receptor pseudogene 112	-1,84	0,040623
17840672	*Asb8*	ankyrin repeat and SOCS box-containing 8	-1,85	0,000814
17813989	*Ston1*	stonin 1	-1,85	0,018934
17818695	*Mir382*	microRNA 382	-1,85	0,011769
17623800	*Lbhd1*	LBH domain containing 1	-1,85	0,032317

17769526	*Mtg2*	mitochondrial ribosome-associated GTPase 2	-1,85	0,021165
17719555	*Zkscan4*	zinc finger with KRAB and SCAN domains 4	-1,85	0,014992
17723515	*RGD1309362*	similar to interferon-inducible GTPase	-1,86	0,032816
17703307	*Gjb2*	gap junction protein, beta 2	-1,87	0,036258
17819266	*Crip2*	cysteine-rich protein 2	-1,88	0,031332
17735984	*RGD1562550*	similar to hypothetical protein FLJ21657	-1,89	0,010081
17659303	---	---	-1,89	0,037551
17813485	*Nbl1*	neuroblastoma 1, DAN family BMP antagonist	-1,9	0,002312
17776976	*LOC102547230*	uncharacterized LOC102547230	-1,91	0,024084
17617488	*Rgma*	repulsive guidance molecule family member A	-1,91	0,039314
17780027	*Dido1*	death inducer-obliterator 1	-1,91	0,033387
17883057	---	---	-1,91	0,000143
17883065	---	---	-1,91	0,000143
17836714	*Mterf3*	mitochondrial transcription termination factor 3	-1,92	0,027054
17882273	---	---	-1,93	0,020974
17743230	*Gtf2b*	general transcription factor IIB	-1,93	0,046317
17796396	*Itpr2*	inositol 1,4,5-trisphosphate receptor, type 2	-1,94	0,002743
17668368	*Mina*	myc induced nuclear antigen	-1,94	0,004214
17883039	---	---	-1,95	0,004366
17693575	*Slc4a4*	solute carrier family 4, sodium bicarbonate cotransporter, member 4	-1,95	0,02596
17658719	*Gemin4*	gem (nuclear organelle) associated protein 4	-1,97	0,004596
17749998	*Vangl1*	VANGL planar cell polarity protein 1	-1,98	0,044725
17823958	*Itpk1*	inositol-tetrakisphosphate 1-kinase	-1,98	0,046096
17687448	*Lyplal1*	lysophospholipase-like 1	-1,98	0,020557
17868037	*LOC363337*	similar to RIKEN cDNA 1700081O22	-1,99	0,046396
17811417	*Man1c1*	mannosidase, alpha, class 1C, member 1	-2	0,031986
17818084	*Otub2*	OTU deubiquitinase, ubiquitin aldehyde binding 2	-2,01	0,049957
17647982	*Wscd1*	WSC domain containing 1	-2,01	0,014985
17780457	*Prkag2*	protein kinase, AMP-activated, gamma 2 non-catalytic subunit	-2,01	0,039038
17637913	*Mki67*	marker of proliferation Ki-67	-2,03	0,036377
17766437	*Mavs*	mitochondrial antiviral signaling protein	-2,1	0,031824

17739157	---	---	-2,11	0,028453
17769025	*Eya2*	EYA transcriptional coactivator and phosphatase 2	-2,13	0,035164
17883071	---	---	-2,13	0,000657
17697869	*Rcor2*	REST corepressor 2	-2,15	0,00898
17712286	*Zfp703*	zinc finger protein 703	-2,16	0,047628
17869670	---	---	-2,17	0,040113
17719794	*RGD1308147*	similar to expressed sequence AW209491	-2,19	0,000092
17680560	*Mir181b1*	microRNA 181b-1	-2,21	0,038541
17683127	*Ptpn14*	protein tyrosine phosphatase, non-receptor type 14	-2,21	0,03322
17742949	*Ddit4l*	DNA-damage-inducible transcript 4-like	-2,23	0,029361
17878258	*Mir421*	microRNA 421	-2,24	0,019258
17701093	*Nudt13*	nudix (nucleoside diphosphate linked moiety X)-type motif 13	-2,25	0,020326
17869440	---	---	-2,25	0,00068
17883061	---	---	-2,26	0,001239
17818717	*Mir410*	microRNA 410	-2,27	0,000698
17792272	---	---	-2,28	0,028786
17680319	*Rabif*	RAB interacting factor	-2,28	0,03624
17856014	*Pth1r*	parathyroid hormone 1 receptor	-2,29	0,000959
17881095	*Gdpgp1*	GDP-D-glucose phosphorylase 1	-2,31	0,010957
17843480	*Vof16*	ischemia related factor vof-16	-2,35	0,006096
17633545	---	---	-2,35	0,043126
17728592	*LOC100359748*	zinc finger CCCH type, antiviral 1	-2,36	0,004982
17818693	*Mir544*	microRNA 544	-2,36	0,010114
17709299	*Grtp1*	growth hormone regulated TBC protein 1	-2,41	0,036684
17790039	*Iqub*	IQ motif and ubiquitin domain containing	-2,42	0,027833
17623593	*Rcor2*	REST corepressor 2	-2,44	0,035033
17760470	*Rxra*	retinoid X receptor alpha	-2,48	0,028688
17621880	*Adgra1*	adhesion G protein-coupled receptor A1	-2,48	0,000943
17818413	*Mir434*	microRNA 434	-2,51	0,004484
17784364	*Krcc1*	lysine-rich coiled-coil 1	-2,57	0,012588
17883055	---	---	-2,65	0,030044
17792236	*Atoh8*	atonal bHLH transcription factor 8	-2,68	0,03377
17714278	---	---	-2,69	0,022131
17818437	---	---	-2,84	0,000067
17868304	---	---	-2,91	0,004084
