# Supplementary material for: Transcriptomic changes triggered by ouabain in rat cerebellum granule cells: Role of α3- and α1-Na+,K+-ATPase-mediated signaling
Source: PLoS One. 2019 Sep 26;14(9):e0222767. doi: 10.1371/journal.pone.0222767 (PMC6762055; doi:10.1371/journal.pone.0222767)
Supplement: S3 Table — (DOCX) [file pone.0222767.s015.docx]

**Table S3. Upregulated gene sets (GeneOntology – Biological Process) in 1mM ouabain-treated granular neurons significant at FDR < 1%.**

| **NAME** | **SIZE** | **ES** | **NES** | **NOM p-val** | **FDR q-val** |
| --- | --- | --- | --- | --- | --- |
| SENSORY PERCEPTION OF CHEMICAL STIMULUS | 326 | -0.74318 | -3.32094 | 0 | 0 |
| DETECTION OF STIMULUS | 445 | -0.65875 | -2.988 | 0 | 0 |
| POSITIVE REGULATION OF LEUKOCYTE MIGRATION | 93 | -0.66299 | -2.54665 | 0 | 0 |
| REGULATION OF NITRIC OXIDE BIOSYNTHETIC PROCESS | 47 | -0.73218 | -2.50511 | 0 | 0 |
| POSITIVE REGULATION OF LEUKOCYTE CHEMOTAXIS | 69 | -0.67831 | -2.49034 | 0 | 0 |
| REGULATION OF LEUKOCYTE MIGRATION | 126 | -0.61163 | -2.48937 | 0 | 0 |
| REGULATION OF LEUKOCYTE CHEMOTAXIS | 81 | -0.64734 | -2.47285 | 0 | 0 |
| REGULATION OF REACTIVE OXYGEN SPECIES BIOSYNTHETIC PROCESS | 58 | -0.67935 | -2.42414 | 0 | 0 |
| CHEMOKINE MEDIATED SIGNALING PATHWAY | 53 | -0.68824 | -2.40123 | 0 | 0 |
| ACUTE INFLAMMATORY RESPONSE | 62 | -0.6748 | -2.4008 | 0 | 0 |
| ACUTE PHASE RESPONSE | 34 | -0.75997 | -2.39645 | 0 | 0 |
| DEFENSE RESPONSE TO BACTERIUM | 142 | -0.57976 | -2.38635 | 0 | 0 |
| INFLAMMATORY RESPONSE | 359 | -0.53146 | -2.38624 | 0 | 0 |
| POSITIVE REGULATION OF INFLAMMATORY RESPONSE | 98 | -0.60397 | -2.35203 | 0 | 0 |
| POSITIVE REGULATION OF REACTIVE OXYGEN SPECIES BIOSYNTHETIC PROCESS | 43 | -0.69805 | -2.35149 | 0 | 0 |
| REGULATION OF INFLAMMATORY RESPONSE | 246 | -0.53246 | -2.31947 | 0 | 0 |
| RESPONSE TO BACTERIUM | 396 | -0.51325 | -2.31176 | 0 | 0 |
| REGULATION OF NEUTROPHIL CHEMOTAXIS | 21 | -0.78039 | -2.30859 | 0 | 0 |
| CELLULAR RESPONSE TO INTERLEUKIN 1 | 69 | -0.6359 | -2.30565 | 0 | 0 |
| RESPONSE TO MOLECULE OF BACTERIAL ORIGIN | 282 | -0.52854 | -2.30098 | 0 | 0 |
| RESPONSE TO INTERLEUKIN 1 | 94 | -0.60176 | -2.29521 | 0 | 0 |
| POSITIVE REGULATION OF CHEMOTAXIS | 102 | -0.58074 | -2.29282 | 0 | 0 |
| NEGATIVE REGULATION OF PEPTIDASE ACTIVITY | 177 | -0.54813 | -2.28885 | 0 | 0 |
| MONOCYTE CHEMOTAXIS | 29 | -0.73258 | -2.28722 | 0 | 0 |
| REGULATION OF TYROSINE PHOSPHORYLATION OF STAT PROTEIN | 65 | -0.6279 | -2.2863 | 0 | 0 |
| CELL CHEMOTAXIS | 131 | -0.56652 | -2.28614 | 0 | 0 |
| POSITIVE REGULATION OF STAT CASCADE | 69 | -0.61657 | -2.27652 | 0 | 0 |
| POSITIVE REGULATION OF NEUTROPHIL MIGRATION | 23 | -0.76449 | -2.26713 | 0 | 0 |
| POSITIVE REGULATION OF REACTIVE OXYGEN SPECIES METABOLIC PROCESS | 77 | -0.60299 | -2.26339 | 0 | 0 |
| LYMPHOCYTE CHEMOTAXIS | 25 | -0.75198 | -2.26091 | 0 | 0 |
| LYMPHOCYTE MIGRATION | 35 | -0.71433 | -2.25141 | 0 | 0 |
| POSITIVE REGULATION OF RESPONSE TO WOUNDING | 138 | -0.55142 | -2.24161 | 0 | 0 |
| DIGESTION | 102 | -0.57046 | -2.22797 | 0 | 2.84E-05 |
| REGULATION OF LYMPHOCYTE MIGRATION | 31 | -0.70948 | -2.22892 | 0 | 2.92E-05 |
| REGULATION OF STAT CASCADE | 120 | -0.56068 | -2.22912 | 0 | 3.01E-05 |
| REGULATION OF NEUTROPHIL MIGRATION | 26 | -0.73628 | -2.23782 | 0 | 3.10E-05 |
| REGULATION OF GRANULOCYTE CHEMOTAXIS | 30 | -0.71678 | -2.21751 | 0 | 5.38E-05 |
| REGULATION OF CYTOKINE BIOSYNTHETIC PROCESS | 80 | -0.56733 | -2.158 | 0 | 5.47E-05 |
| REGULATION OF LEUKOCYTE DIFFERENTIATION | 197 | -0.52383 | -2.22267 | 0 | 5.53E-05 |
| POSITIVE REGULATION OF ERK1 AND ERK2 CASCADE | 143 | -0.53078 | -2.16845 | 0 | 5.57E-05 |
| MYELOID LEUKOCYTE MIGRATION | 77 | -0.57611 | -2.16906 | 0 | 5.67E-05 |
| POSITIVE REGULATION OF LEUKOCYTE DIFFERENTIATION | 115 | -0.54501 | -2.17109 | 0 | 5.78E-05 |
| REGULATION OF ERK1 AND ERK2 CASCADE | 199 | -0.5175 | -2.1721 | 0 | 5.89E-05 |
| REGULATION OF T CELL DIFFERENTIATION | 91 | -0.56777 | -2.18 | 0 | 6.01E-05 |
| LEUKOCYTE CHEMOTAXIS | 94 | -0.56363 | -2.18743 | 0 | 6.13E-05 |
| REGULATION OF ENDOCRINE PROCESS | 43 | -0.64091 | -2.18864 | 0 | 6.25E-05 |
| REGULATION OF LYMPHOCYTE DIFFERENTIATION | 112 | -0.55813 | -2.18876 | 0 | 6.38E-05 |
| POSITIVE REGULATION OF PEPTIDYL TYROSINE PHOSPHORYLATION | 153 | -0.53128 | -2.18935 | 0 | 6.52E-05 |
| REGULATION OF TYPE 2 IMMUNE RESPONSE | 24 | -0.73757 | -2.18954 | 0 | 6.66E-05 |
| SENSORY PERCEPTION OF TASTE | 46 | -0.64582 | -2.19078 | 0 | 6.81E-05 |
| POSITIVE REGULATION OF ACUTE INFLAMMATORY RESPONSE | 27 | -0.71951 | -2.19185 | 0 | 6.96E-05 |
| REGULATION OF ACUTE INFLAMMATORY RESPONSE | 64 | -0.58985 | -2.15 | 0 | 7.03E-05 |
| RESPONSE TO CORTICOSTEROID | 163 | -0.52858 | -2.19338 | 0 | 7.12E-05 |
| NEGATIVE REGULATION OF HORMONE SECRETION | 66 | -0.59721 | -2.15514 | 0 | 7.15E-05 |
| REGULATION OF RESPONSE TO WOUNDING | 348 | -0.48855 | -2.1946 | 0 | 7.29E-05 |
| POSITIVE REGULATION OF HEMOPOIESIS | 143 | -0.53816 | -2.19529 | 0 | 7.47E-05 |
| OVULATION | 16 | -0.81935 | -2.19906 | 0 | 7.66E-05 |
| POSITIVE REGULATION OF RESPONSE TO EXTERNAL STIMULUS | 245 | -0.50645 | -2.20031 | 0 | 7.85E-05 |
| POSITIVE REGULATION OF TYROSINE PHOSPHORYLATION OF STAT3 PROTEIN | 36 | -0.66239 | -2.14438 | 0 | 1.38E-04 |
| HUMORAL IMMUNE RESPONSE | 100 | -0.55236 | -2.14006 | 0 | 1.53E-04 |
| DEFENSE RESPONSE TO GRAM POSITIVE BACTERIUM | 48 | -0.62733 | -2.13896 | 0 | 1.83E-04 |
| REGULATION OF TYROSINE PHOSPHORYLATION OF STAT3 PROTEIN | 42 | -0.63789 | -2.13157 | 0 | 2.46E-04 |
| REGULATION OF INTERFERON GAMMA PRODUCTION | 77 | -0.56687 | -2.12851 | 0 | 2.74E-04 |
| REGULATION OF VASCULAR ENDOTHELIAL GROWTH FACTOR PRODUCTION | 28 | -0.69438 | -2.12667 | 0 | 2.86E-04 |
| GRANULOCYTE MIGRATION | 57 | -0.59261 | -2.11984 | 0 | 3.75E-04 |
| HORMONE TRANSPORT | 67 | -0.59371 | -2.11043 | 0 | 3.83E-04 |
| REGULATION OF VASCULATURE DEVELOPMENT | 198 | -0.50059 | -2.11513 | 0 | 3.85E-04 |
| REGULATION OF PEPTIDYL TYROSINE PHOSPHORYLATION | 196 | -0.49866 | -2.11048 | 0 | 3.88E-04 |
| REGULATION OF LYMPHOCYTE CHEMOTAXIS | 16 | -0.79371 | -2.11207 | 0 | 3.94E-04 |
| REGULATION OF LEUKOCYTE PROLIFERATION | 174 | -0.50855 | -2.10512 | 0 | 4.04E-04 |
| NEUROPEPTIDE SIGNALING PATHWAY | 86 | -0.54708 | -2.10519 | 0 | 4.09E-04 |
| POSITIVE REGULATION OF LEUKOCYTE PROLIFERATION | 119 | -0.52708 | -2.10637 | 0 | 4.15E-04 |
| FEEDING BEHAVIOR | 78 | -0.55832 | -2.10724 | 0 | 4.21E-04 |
| REGULATION OF CYTOKINE SECRETION | 119 | -0.52835 | -2.09799 | 0 | 4.81E-04 |
| NEGATIVE REGULATION OF ADAPTIVE IMMUNE RESPONSE | 30 | -0.66359 | -2.09518 | 0 | 5.01E-04 |
| DETECTION OF CHEMICAL STIMULUS INVOLVED IN SENSORY PERCEPTION OF TASTE | 29 | -0.67413 | -2.0902 | 0 | 5.54E-04 |
| POSITIVE REGULATION OF LYMPHOCYTE MIGRATION | 22 | -0.72652 | -2.08832 | 0 | 5.60E-04 |
| CYTOKINE MEDIATED SIGNALING PATHWAY | 357 | -0.46551 | -2.0907 | 0 | 5.61E-04 |
| REGULATION OF REACTIVE OXYGEN SPECIES METABOLIC PROCESS | 134 | -0.51169 | -2.08703 | 0 | 5.66E-04 |
| POSITIVE REGULATION OF T CELL PROLIFERATION | 82 | -0.54335 | -2.08301 | 0 | 6.11E-04 |
| ALPHA BETA T CELL DIFFERENTIATION | 38 | -0.64456 | -2.08213 | 0 | 6.16E-04 |
| T CELL ACTIVATION INVOLVED IN IMMUNE RESPONSE | 38 | -0.63457 | -2.08089 | 0 | 6.21E-04 |
| RESPONSE TO VITAMIN D | 30 | -0.66359 | -2.07821 | 0 | 6.50E-04 |
| REGULATION OF CD4 POSITIVE ALPHA BETA T CELL ACTIVATION | 32 | -0.64912 | -2.07658 | 0 | 6.55E-04 |
| NEGATIVE REGULATION OF COAGULATION | 45 | -0.61423 | -2.07315 | 0 | 7.19E-04 |
| POSITIVE REGULATION OF LYMPHOCYTE DIFFERENTIATION | 70 | -0.55913 | -2.07198 | 0 | 7.35E-04 |
| POSITIVE REGULATION OF PROTEIN KINASE B SIGNALING | 68 | -0.57025 | -2.06646 | 0 | 7.97E-04 |
| NEGATIVE REGULATION OF CYTOKINE SECRETION | 36 | -0.64041 | -2.06334 | 0 | 8.34E-04 |
| CELLULAR DEFENSE RESPONSE | 43 | -0.6095 | -2.05708 | 0 | 8.43E-04 |
| POSITIVE REGULATION OF LYMPHOCYTE MEDIATED IMMUNITY | 61 | -0.57193 | -2.05606 | 0 | 8.45E-04 |
| REGULATION OF T CELL PROLIFERATION | 123 | -0.51734 | -2.06204 | 0 | 8.48E-04 |
| ADAPTIVE IMMUNE RESPONSE | 190 | -0.48656 | -2.05822 | 0 | 8.52E-04 |
| NEGATIVE REGULATION OF HOMOTYPIC CELL CELL ADHESION | 82 | -0.54367 | -2.05341 | 0 | 8.58E-04 |
| POSITIVE REGULATION OF LEUKOCYTE MEDIATED IMMUNITY | 77 | -0.54889 | -2.05832 | 0 | 8.62E-04 |
| ALPHA BETA T CELL ACTIVATION | 46 | -0.59902 | -2.05273 | 0 | 8.70E-04 |
| REGULATION OF HEMOPOIESIS | 256 | -0.47516 | -2.05163 | 0 | 8.83E-04 |
| LIPID DIGESTION | 18 | -0.73423 | -2.04905 | 0.0017699 | 8.96E-04 |
| NEGATIVE REGULATION OF INTERFERON GAMMA PRODUCTION | 28 | -0.6691 | -2.04916 | 0 | 9.05E-04 |
| NEGATIVE REGULATION OF RESPONSE TO WOUNDING | 134 | -0.50442 | -2.04701 | 0 | 9.18E-04 |
| REGULATION OF LYMPHOCYTE MEDIATED IMMUNITY | 95 | -0.52858 | -2.04536 | 0 | 9.39E-04 |
| POSITIVE REGULATION OF BLOOD CIRCULATION | 87 | -0.53954 | -2.04277 | 0 | 9.81E-04 |
| POSITIVE REGULATION OF CYTOKINE BIOSYNTHETIC PROCESS | 49 | -0.59854 | -2.0419 | 0 | 9.91E-04 |
| REGULATION OF ALPHA BETA T CELL ACTIVATION | 58 | -0.57868 | -2.04056 | 0 | 0.0010012 |
| POSITIVE REGULATION OF VASCULATURE DEVELOPMENT | 114 | -0.51153 | -2.03581 | 0 | 0.0010994 |
| ENDOCRINE PROCESS | 39 | -0.61863 | -2.03483 | 0 | 0.001118 |
| REGULATION OF HOMOTYPIC CELL CELL ADHESION | 260 | -0.4669 | -2.03249 | 0 | 0.0011255 |
| POSITIVE REGULATION OF CYTOKINE PRODUCTION | 306 | -0.46249 | -2.03327 | 0 | 0.0011361 |
| POSITIVE REGULATION OF CALCIUM ION IMPORT | 46 | -0.58795 | -2.02744 | 0 | 0.0011957 |
| DEFENSE RESPONSE TO GRAM NEGATIVE BACTERIUM | 35 | -0.63679 | -2.03022 | 0 | 0.0012006 |
| CD4 POSITIVE ALPHA BETA T CELL ACTIVATION | 29 | -0.65596 | -2.02776 | 0 | 0.0012066 |
| REGULATION OF ALPHA BETA T CELL DIFFERENTIATION | 40 | -0.60779 | -2.0283 | 0 | 0.0012177 |
| RESPONSE TO FUNGUS | 37 | -0.62347 | -2.0254 | 0 | 0.0012486 |
| REGULATION OF CHEMOTAXIS | 151 | -0.49548 | -2.0205 | 0 | 0.0013162 |
| NEGATIVE REGULATION OF CELL CELL ADHESION | 109 | -0.51167 | -2.02104 | 0 | 0.0013187 |
| NEGATIVE REGULATION OF CELL ACTIVATION | 128 | -0.50666 | -2.01288 | 0 | 0.0014167 |
| POSITIVE REGULATION OF MYELOID CELL DIFFERENTIATION | 72 | -0.54701 | -2.01385 | 0 | 0.0014203 |
| POSITIVE REGULATION OF B CELL MEDIATED IMMUNITY | 24 | -0.67496 | -2.00967 | 0 | 0.0014575 |
| NEGATIVE REGULATION OF WOUND HEALING | 51 | -0.58101 | -2.00647 | 0 | 0.0015574 |
| CELLULAR RESPONSE TO VITAMIN | 25 | -0.67633 | -2.00441 | 0 | 0.0015949 |
| NEGATIVE REGULATION OF LEUKOCYTE DIFFERENTIATION | 65 | -0.5584 | -2.00485 | 0 | 0.0015997 |
| NEGATIVE REGULATION OF SECRETION | 173 | -0.48055 | -2.00528 | 0 | 0.0016044 |
| REGULATION OF CELL KILLING | 55 | -0.56769 | -2.00234 | 0 | 0.0016436 |
| REGULATION OF B CELL ACTIVATION | 88 | -0.53067 | -2.00272 | 0 | 0.0016485 |
| T CELL DIFFERENTIATION INVOLVED IN IMMUNE RESPONSE | 24 | -0.67428 | -2.0013 | 0 | 0.0016551 |
| POSITIVE REGULATION OF SMOOTH MUSCLE CELL PROLIFERATION | 57 | -0.55745 | -1.99683 | 0 | 0.0017748 |
| REGULATION OF LEUKOCYTE MEDIATED CYTOTOXICITY | 45 | -0.58194 | -1.99705 | 0 | 0.001789 |
| REGULATION OF MULTICELLULAR ORGANISMAL METABOLIC PROCESS | 35 | -0.6206 | -1.99251 | 0 | 0.0019299 |
| LEUKOCYTE CELL CELL ADHESION | 214 | -0.46944 | -1.99048 | 0 | 0.0020028 |
| REGULATION OF TRANSCRIPTION INVOLVED IN CELL FATE COMMITMENT | 18 | -0.73213 | -1.98896 | 0 | 0.0020033 |
| INFLAMMATORY RESPONSE TO ANTIGENIC STIMULUS | 21 | -0.69716 | -1.98849 | 0 | 0.0020269 |
| G PROTEIN COUPLED RECEPTOR SIGNALING PATHWAY COUPLED TO CYCLIC NUCLEOTIDE SECOND MESSENGER | 152 | -0.48329 | -1.98747 | 0 | 0.0020422 |
| REGULATION OF B CELL MEDIATED IMMUNITY | 36 | -0.61008 | -1.98545 | 0 | 0.002065 |
| POSITIVE REGULATION OF LOCOMOTION | 353 | -0.44333 | -1.98584 | 0 | 0.0020652 |
| REGULATION OF T HELPER CELL DIFFERENTIATION | 22 | -0.69764 | -1.98233 | 0 | 0.0021095 |
| REGULATION OF CYCLIC NUCLEOTIDE METABOLIC PROCESS | 134 | -0.48976 | -1.98359 | 0 | 0.0021107 |
| POSITIVE REGULATION OF IMMUNE EFFECTOR PROCESS | 132 | -0.49277 | -1.98264 | 0 | 0.0021251 |
| NEGATIVE REGULATION OF LYMPHOCYTE DIFFERENTIATION | 31 | -0.61766 | -1.98067 | 0 | 0.0021684 |
| REGULATION OF ADAPTIVE IMMUNE RESPONSE | 104 | -0.50647 | -1.97768 | 0 | 0.0022094 |
| CELLULAR RESPONSE TO CYTOKINE STIMULUS | 485 | -0.42953 | -1.97883 | 0 | 0.0022106 |
| REGULATION OF CYTOKINE PRODUCTION | 455 | -0.43456 | -1.9799 | 0 | 0.0022117 |
| POSITIVE REGULATION OF B CELL ACTIVATION | 59 | -0.55951 | -1.97578 | 0 | 0.0022145 |
| REGULATION OF CELL CELL ADHESION | 322 | -0.44265 | -1.97587 | 0 | 0.0022302 |
| OVULATION CYCLE | 104 | -0.50875 | -1.97515 | 0 | 0.0022346 |
| MORPHOGENESIS OF A BRANCHING STRUCTURE | 145 | -0.4838 | -1.97156 | 0 | 0.0023114 |
| T CELL DIFFERENTIATION | 109 | -0.50214 | -1.96969 | 0 | 0.0023588 |
| REGULATION OF CELL ACTIVATION | 396 | -0.43504 | -1.96919 | 0 | 0.0023637 |
| ORGAN OR TISSUE SPECIFIC IMMUNE RESPONSE | 22 | -0.69498 | -1.96634 | 0 | 0.0024382 |
| FEMALE GAMETE GENERATION | 79 | -0.52198 | -1.96367 | 0 | 0.0025319 |
| NEGATIVE REGULATION OF HYDROLASE ACTIVITY | 300 | -0.44628 | -1.96103 | 0 | 0.0025762 |
| REGULATION OF MYELOID LEUKOCYTE DIFFERENTIATION | 94 | -0.51036 | -1.96106 | 0 | 0.0025934 |
| MULTI MULTICELLULAR ORGANISM PROCESS | 184 | -0.46383 | -1.95841 | 0 | 0.0025991 |
| REGULATION OF INTERLEUKIN 6 PRODUCTION | 85 | -0.51738 | -1.96145 | 0 | 0.0026108 |
| RESPONSE TO PROSTAGLANDIN | 29 | -0.64291 | -1.95867 | 0 | 0.0026161 |
| FIBRINOLYSIS | 20 | -0.69125 | -1.95892 | 0 | 0.0026333 |
| POSITIVE REGULATION OF CYTOKINE SECRETION | 79 | -0.52171 | -1.95734 | 0 | 0.0026815 |
| REGULATION OF LEUKOCYTE MEDIATED IMMUNITY | 131 | -0.4801 | -1.95591 | 0 | 0.002723 |
| RESPONSE TO INTERLEUKIN 6 | 24 | -0.65002 | -1.95381 | 0 | 0.0027578 |
| REGULATION OF IMMUNOGLOBULIN PRODUCTION | 42 | -0.58775 | -1.95314 | 0 | 0.0027921 |
| REGULATION OF SYSTEMIC ARTERIAL BLOOD PRESSURE BY HORMONE | 30 | -0.62467 | -1.95192 | 0 | 0.0028208 |
| OVULATION CYCLE PROCESS | 80 | -0.52479 | -1.95246 | 0 | 0.0028257 |
| REGULATION OF SMOOTH MUSCLE CELL PROLIFERATION | 94 | -0.50608 | -1.95122 | 0.0014451 | 0.0028285 |
| RESPONSE TO CORTICOSTERONE | 22 | -0.67263 | -1.94994 | 0 | 0.0028611 |
| POSITIVE REGULATION OF IMMUNOGLOBULIN PRODUCTION | 29 | -0.6311 | -1.94724 | 0 | 0.0029752 |
| REGULATION OF CALCIUM ION IMPORT | 83 | -0.5156 | -1.94644 | 0 | 0.0030068 |
| T CELL SELECTION | 32 | -0.61672 | -1.9454 | 0.0016584 | 0.0030133 |
| DEFENSE RESPONSE TO FUNGUS | 24 | -0.64524 | -1.94375 | 0 | 0.0030815 |
| NEGATIVE REGULATION OF PROTEOLYSIS | 249 | -0.44654 | -1.94093 | 0 | 0.0031479 |
| POSITIVE REGULATION OF PEPTIDYL SERINE PHOSPHORYLATION | 63 | -0.53315 | -1.94165 | 0 | 0.003161 |
| REGULATION OF CELL FATE COMMITMENT | 23 | -0.66701 | -1.94095 | 0 | 0.0031666 |
| TAXIS | 385 | -0.43231 | -1.94001 | 0 | 0.0031896 |
| POSITIVE REGULATION OF CELL CELL ADHESION | 210 | -0.45295 | -1.93659 | 0 | 0.0032028 |
| LEUKOCYTE MIGRATION | 221 | -0.44885 | -1.93679 | 0 | 0.0032034 |
| CELLULAR RESPONSE TO FLUID SHEAR STRESS | 17 | -0.71552 | -1.93906 | 0 | 0.0032193 |
| REGULATION OF PROTEIN KINASE B SIGNALING | 104 | -0.49366 | -1.9369 | 0 | 0.0032217 |
| REGULATION OF CAMP METABOLIC PROCESS | 111 | -0.48922 | -1.93757 | 0 | 0.0032224 |
| ANTIMICROBIAL HUMORAL RESPONSE | 27 | -0.63543 | -1.93778 | 0.0017271 | 0.0032292 |
| REGULATION OF EPITHELIAL CELL APOPTOTIC PROCESS | 50 | -0.56182 | -1.93594 | 0 | 0.0032306 |
| CELLULAR RESPONSE TO BIOTIC STIMULUS | 146 | -0.47406 | -1.93801 | 0 | 0.0032479 |
| BIOMINERAL TISSUE DEVELOPMENT | 66 | -0.52664 | -1.93348 | 0 | 0.0033207 |
| SKELETAL MUSCLE CELL DIFFERENTIATION | 46 | -0.56347 | -1.93143 | 0 | 0.0033517 |
| SARCOMERE ORGANIZATION | 21 | -0.65843 | -1.93149 | 0.0034904 | 0.0033647 |
| NEGATIVE REGULATION OF PROTEIN SECRETION | 91 | -0.50391 | -1.92973 | 0 | 0.0034346 |
| REGULATION OF PEPTIDASE ACTIVITY | 306 | -0.4347 | -1.92845 | 0 | 0.0034885 |
| REGULATION OF CELLULAR EXTRAVASATION | 21 | -0.65632 | -1.92522 | 0 | 0.0036255 |
| GLAND MORPHOGENESIS | 91 | -0.49394 | -1.92424 | 0 | 0.0036468 |
| REGULATION OF B CELL PROLIFERATION | 49 | -0.55907 | -1.92464 | 0 | 0.0036501 |
| EPITHELIAL TUBE BRANCHING INVOLVED IN LUNG MORPHOGENESIS | 18 | -0.70563 | -1.92211 | 0.0017986 | 0.0037559 |
| POSITIVE REGULATION OF CELL ACTIVATION | 251 | -0.44254 | -1.92229 | 0 | 0.0037759 |
| EATING BEHAVIOR | 28 | -0.62571 | -1.92244 | 0 | 0.0037852 |
| RESPONSE TO KETONE | 167 | -0.46255 | -1.92022 | 0 | 0.0038075 |
| LUNG MORPHOGENESIS | 37 | -0.58534 | -1.92061 | 0 | 0.0038168 |
| REGULATION OF MACROPHAGE DIFFERENTIATION | 20 | -0.68658 | -1.91869 | 0 | 0.0038727 |
| DETECTION OF VISIBLE LIGHT | 35 | -0.59593 | -1.91766 | 0 | 0.0039065 |
| RESPONSE TO DEXAMETHASONE | 31 | -0.59859 | -1.9179 | 0 | 0.0039109 |
| REGULATION OF ODONTOGENESIS | 24 | -0.65548 | -1.91478 | 0.0017575 | 0.0040231 |
| FERTILIZATION | 116 | -0.47972 | -1.91341 | 0 | 0.0041016 |
| REGULATION OF SYSTEM PROCESS | 451 | -0.41868 | -1.91303 | 0 | 0.0041063 |
| REGULATION OF IMMUNOGLOBULIN SECRETION | 17 | -0.7068 | -1.91344 | 0.0018018 | 0.0041172 |
| POSITIVE REGULATION OF CYCLIC NUCLEOTIDE METABOLIC PROCESS | 98 | -0.4944 | -1.9077 | 0 | 0.0043462 |
| DECIDUALIZATION | 19 | -0.68934 | -1.90805 | 0.0017762 | 0.0043579 |
| POSITIVE REGULATION OF VASODILATION | 30 | -0.61774 | -1.90475 | 0.0016393 | 0.0044362 |
| REGULATION OF METANEPHROS DEVELOPMENT | 20 | -0.67301 | -1.90219 | 0.0034247 | 0.0045717 |
| POSITIVE REGULATION OF CD4 POSITIVE ALPHA BETA T CELL ACTIVATION | 25 | -0.63437 | -1.9017 | 0 | 0.0045743 |
| STAT CASCADE | 41 | -0.57548 | -1.89719 | 0 | 0.0048254 |
| RESPONSE TO TUMOR NECROSIS FACTOR | 190 | -0.44688 | -1.89721 | 0 | 0.0048489 |
| REGULATION OF INSULIN RECEPTOR SIGNALING PATHWAY | 38 | -0.5731 | -1.89723 | 0.0016556 | 0.0048727 |
| KERATINOCYTE DIFFERENTIATION | 60 | -0.52792 | -1.89442 | 0 | 0.0049847 |
| POSITIVE REGULATION OF INTERLEUKIN 6 PRODUCTION | 56 | -0.53793 | -1.89303 | 0 | 0.0050595 |
| NEGATIVE REGULATION OF ION TRANSPORT | 107 | -0.47519 | -1.88908 | 0 | 0.0052596 |
| POSITIVE REGULATION OF CELL KILLING | 34 | -0.58309 | -1.88847 | 0 | 0.0052682 |
| PHENOL CONTAINING COMPOUND BIOSYNTHETIC PROCESS | 30 | -0.60553 | -1.88863 | 0 | 0.0052786 |
| LYMPHOCYTE DIFFERENTIATION | 171 | -0.45451 | -1.88909 | 0 | 0.0052848 |
| REGULATION OF CAMP MEDIATED SIGNALING | 22 | -0.65513 | -1.88795 | 0.0017452 | 0.0053009 |
| NEGATIVE REGULATION OF PROTEIN MATURATION | 25 | -0.62895 | -1.88668 | 0 | 0.0053381 |
| LEUKOCYTE DIFFERENTIATION | 243 | -0.43414 | -1.88512 | 0 | 0.0054274 |
| DEFENSE RESPONSE TO OTHER ORGANISM | 323 | -0.42493 | -1.88321 | 0 | 0.0055348 |
| RESPONSE TO INTERFERON GAMMA | 100 | -0.48297 | -1.8807 | 0 | 0.0057211 |
| POSITIVE REGULATION OF NUCLEOTIDE METABOLIC PROCESS | 120 | -0.46938 | -1.87706 | 0 | 0.0060329 |
| REGULATION OF HORMONE LEVELS | 416 | -0.41056 | -1.87376 | 0 | 0.0062806 |
| CATECHOLAMINE BIOSYNTHETIC PROCESS | 17 | -0.68313 | -1.87281 | 0.0035651 | 0.0062985 |
| ADENYLATE CYCLASE MODULATING G PROTEIN COUPLED RECEPTOR SIGNALING PATHWAY | 128 | -0.47062 | -1.87214 | 0 | 0.0063347 |
| RESPONSE TO PAIN | 27 | -0.61473 | -1.8712 | 0 | 0.006403 |
| RESPONSE TO MINERALOCORTICOID | 30 | -0.60523 | -1.87054 | 0 | 0.00642 |
| POSITIVE REGULATION OF ADAPTIVE IMMUNE RESPONSE | 66 | -0.51757 | -1.86898 | 0 | 0.0065141 |
| REGULATION OF REPRODUCTIVE PROCESS | 117 | -0.47765 | -1.86687 | 0 | 0.0066575 |
| REGULATION OF ENDOTHELIAL CELL APOPTOTIC PROCESS | 35 | -0.57398 | -1.86506 | 0 | 0.0067828 |
| REGULATION OF NUCLEOTIDE METABOLIC PROCESS | 182 | -0.44312 | -1.86443 | 0 | 0.0067904 |
| CALCIUM INDEPENDENT CELL CELL ADHESION VIA PLASMA MEMBRANE CELL ADHESION MOLECULES | 21 | -0.65641 | -1.86462 | 0.0056604 | 0.006807 |
| REGULATION OF VASOCONSTRICTION | 62 | -0.5176 | -1.86288 | 0 | 0.0068381 |
| LEUKOCYTE ACTIVATION | 336 | -0.41676 | -1.8632 | 0 | 0.0068548 |
| POSITIVE REGULATION OF SYNAPTIC TRANSMISSION GLUTAMATERGIC | 15 | -0.69886 | -1.86095 | 0.0018215 | 0.0069585 |
| BLOOD COAGULATION FIBRIN CLOT FORMATION | 22 | -0.63746 | -1.86026 | 0.0017889 | 0.0069814 |
| HYALURONAN METABOLIC PROCESS | 27 | -0.61454 | -1.85895 | 0.0034965 | 0.0070569 |
| NEGATIVE REGULATION OF INFLAMMATORY RESPONSE | 85 | -0.48836 | -1.85679 | 0.0014684 | 0.0071838 |
| CELLULAR RESPONSE TO ALCOHOL | 106 | -0.47212 | -1.85548 | 0 | 0.0072796 |
| NEGATIVE REGULATION OF IMMUNE SYSTEM PROCESS | 293 | -0.42269 | -1.85357 | 0 | 0.0074439 |
| RESPONSE TO PURINE CONTAINING COMPOUND | 143 | -0.45262 | -1.85297 | 0 | 0.0074643 |
| NEGATIVE REGULATION OF MYELOID LEUKOCYTE DIFFERENTIATION | 36 | -0.57336 | -1.85077 | 0 | 0.0075725 |
| REGULATION OF MYELOID CELL DIFFERENTIATION | 149 | -0.44838 | -1.85088 | 0 | 0.0076 |
| NEGATIVE REGULATION OF T CELL DIFFERENTIATION | 25 | -0.62812 | -1.84941 | 0.0017007 | 0.0076984 |
| REGULATION OF DIGESTIVE SYSTEM PROCESS | 32 | -0.58373 | -1.8486 | 0 | 0.007736 |
| POSITIVE REGULATION OF INTERFERON GAMMA PRODUCTION | 52 | -0.53707 | -1.8487 | 0 | 0.0077639 |
| CELL FATE COMMITMENT | 200 | -0.43812 | -1.84795 | 0 | 0.0077673 |
| NEGATIVE REGULATION OF ALPHA BETA T CELL ACTIVATION | 17 | -0.68641 | -1.84724 | 0.0017544 | 0.0077984 |
| REGULATION OF SYSTEMIC ARTERIAL BLOOD PRESSURE MEDIATED BY A CHEMICAL SIGNAL | 40 | -0.56516 | -1.84631 | 0 | 0.0079377 |
| POSITIVE REGULATION OF CAMP METABOLIC PROCESS | 80 | -0.49746 | -1.84613 | 0 | 0.007943 |
| POSITIVE REGULATION OF ALPHA BETA T CELL DIFFERENTIATION | 34 | -0.58209 | -1.84563 | 0.0016949 | 0.0079567 |
| NEGATIVE REGULATION OF MAP KINASE ACTIVITY | 60 | -0.50799 | -1.84333 | 0 | 0.0080938 |
| POSITIVE REGULATION OF RELEASE OF SEQUESTERED CALCIUM ION INTO CYTOSOL | 32 | -0.58061 | -1.84393 | 0.0016949 | 0.0080976 |
| FEMALE SEX DIFFERENTIATION | 104 | -0.47077 | -1.84157 | 0 | 0.0082992 |
| POSITIVE REGULATION OF B CELL PROLIFERATION | 34 | -0.57912 | -1.84062 | 0 | 0.0083559 |
| NEGATIVE REGULATION OF BLOOD CIRCULATION | 28 | -0.6048 | -1.83854 | 0.0016287 | 0.008578 |
| REGULATION OF ALPHA BETA T CELL PROLIFERATION | 21 | -0.64187 | -1.83544 | 0.0017241 | 0.0089354 |
| POSITIVE REGULATION OF PROTEIN SECRETION | 179 | -0.43247 | -1.83369 | 0 | 0.0089746 |
| EXTRACELLULAR MATRIX DISASSEMBLY | 63 | -0.51083 | -1.83331 | 0 | 0.0089753 |
| REGULATION OF HORMONE SECRETION | 234 | -0.42172 | -1.83388 | 0 | 0.0089818 |
| NEGATIVE REGULATION OF HEMOPOIESIS | 95 | -0.47015 | -1.83449 | 0.0014225 | 0.008997 |
| REGULATION OF LYASE ACTIVITY | 73 | -0.50237 | -1.83158 | 0 | 0.0090953 |
| NEGATIVE REGULATION OF CYTOKINE BIOSYNTHETIC PROCESS | 24 | -0.62181 | -1.83114 | 0 | 0.0091112 |
| POSITIVE REGULATION OF T HELPER CELL DIFFERENTIATION | 16 | -0.67326 | -1.8316 | 0.0054945 | 0.0091265 |
| ENDOCRINE PANCREAS DEVELOPMENT | 33 | -0.5732 | -1.83 | 0.0033501 | 0.0091706 |
| CELLULAR RESPONSE TO INTERLEUKIN 6 | 20 | -0.64815 | -1.82957 | 0 | 0.0091902 |
| LEUKOCYTE MEDIATED IMMUNITY | 123 | -0.45616 | -1.83 | 0 | 0.0092057 |
| POSITIVE REGULATION OF TYROSINE PHOSPHORYLATION OF STAT5 PROTEIN | 16 | -0.66061 | -1.8282 | 0.001845 | 0.0092989 |
| POSITIVE REGULATION OF MYELOID LEUKOCYTE DIFFERENTIATION | 45 | -0.54061 | -1.82707 | 0.004644 | 0.0094019 |
| CELLULAR RESPONSE TO KETONE | 67 | -0.50609 | -1.82726 | 0 | 0.0094183 |
| NEGATIVE REGULATION OF B CELL ACTIVATION | 24 | -0.60692 | -1.82571 | 0.005386 | 0.0095426 |
| REGULATION OF FAT CELL DIFFERENTIATION | 88 | -0.48074 | -1.82418 | 0.002849 | 0.0097083 |
| REGULATION OF VASODILATION | 44 | -0.54348 | -1.82201 | 0.0016103 | 0.0099944 |
