# Supplementary material for: Transcriptomic changes triggered by ouabain in rat cerebellum granule cells: Role of α3- and α1-Na+,K+-ATPase-mediated signaling
Source: PLoS One. 2019 Sep 26;14(9):e0222767. doi: 10.1371/journal.pone.0222767 (PMC6762055; doi:10.1371/journal.pone.0222767)
Supplement: S4 Table — (DOCX) [file pone.0222767.s016.docx]

**Table S4. Downregulated gene sets (GeneOntology – Biological Process) in 1mM ouabain-treated granular neurons significant at FDR < 1%.**

| **NAME** | **SIZE** | **ES** | **NES** | **NOM p-val** | **FDR q-val** |
| --- | --- | --- | --- | --- | --- |
| TRNA METABOLIC PROCESS | 139 | 0.570832 | 2.617057 | 0 | 0 |
| NCRNA PROCESSING | 292 | 0.516053 | 2.594902 | 0 | 0 |
| RIBOSOME BIOGENESIS | 232 | 0.516068 | 2.552908 | 0 | 0 |
| RIBONUCLEOPROTEIN COMPLEX BIOGENESIS | 336 | 0.488808 | 2.539933 | 0 | 0 |
| TELOMERE ORGANIZATION | 65 | 0.630175 | 2.52917 | 0 | 0 |
| NCRNA METABOLIC PROCESS | 412 | 0.481516 | 2.518002 | 0 | 0 |
| MITOCHONDRIAL TRANSLATION | 94 | 0.56708 | 2.438949 | 0 | 0 |
| DNA BIOSYNTHETIC PROCESS | 94 | 0.567739 | 2.43363 | 0 | 0 |
| RRNA METABOLIC PROCESS | 190 | 0.501994 | 2.427901 | 0 | 0 |
| TRANSLESION SYNTHESIS | 35 | 0.666229 | 2.370357 | 0 | 9.89E-05 |
| TRANSLATIONAL TERMINATION | 85 | 0.56047 | 2.351869 | 0 | 8.99E-05 |
| TRNA PROCESSING | 88 | 0.549497 | 2.343358 | 0 | 1.63E-04 |
| NUCLEOTIDE EXCISION REPAIR DNA INCISION | 33 | 0.647733 | 2.324865 | 0 | 2.24E-04 |
| TRANSLATIONAL ELONGATION | 98 | 0.538238 | 2.315272 | 0 | 2.08E-04 |
| AMINO ACID ACTIVATION | 45 | 0.625651 | 2.307543 | 0 | 2.59E-04 |
| DNA TEMPLATED TRANSCRIPTION TERMINATION | 81 | 0.545419 | 2.292567 | 0 | 2.42E-04 |
| SNRNA METABOLIC PROCESS | 68 | 0.572254 | 2.28437 | 0 | 2.28E-04 |
| TRANSCRIPTION COUPLED NUCLEOTIDE EXCISION REPAIR | 65 | 0.559098 | 2.277123 | 0 | 2.15E-04 |
| POSTREPLICATION REPAIR | 46 | 0.60622 | 2.272803 | 0 | 2.04E-04 |
| DNA SYNTHESIS INVOLVED IN DNA REPAIR | 58 | 0.570738 | 2.258943 | 0 | 2.42E-04 |
| CELLULAR PROTEIN COMPLEX DISASSEMBLY | 110 | 0.498005 | 2.257505 | 0 | 2.30E-04 |
| DNA STRAND ELONGATION | 28 | 0.674303 | 2.22757 | 0 | 3.94E-04 |
| TELOMERE MAINTENANCE VIA RECOMBINATION | 28 | 0.691863 | 2.223045 | 0 | 3.77E-04 |
| RNA SPLICING VIA TRANSESTERIFICATION REACTIONS | 220 | 0.455342 | 2.214026 | 0 | 3.61E-04 |
| NCRNA TRANSCRIPTION | 72 | 0.5389 | 2.212296 | 0 | 3.47E-04 |
| RNA MODIFICATION | 90 | 0.510838 | 2.189754 | 0 | 4.08E-04 |
| NUCLEOTIDE EXCISION REPAIR | 96 | 0.505201 | 2.17966 | 0 | 4.30E-04 |
| SPLICEOSOMAL SNRNP ASSEMBLY | 30 | 0.632487 | 2.16062 | 0 | 4.82E-04 |
| REGULATION OF CELLULAR RESPONSE TO HEAT | 59 | 0.548341 | 2.158394 | 0 | 4.98E-04 |
| TRANSCRIPTION FROM RNA POLYMERASE III PROMOTER | 32 | 0.622696 | 2.157662 | 0 | 4.82E-04 |
| RIBONUCLEOPROTEIN COMPLEX SUBUNIT ORGANIZATION | 153 | 0.458013 | 2.156773 | 0 | 4.66E-04 |
| NUCLEOTIDE EXCISION REPAIR DNA GAP FILLING | 22 | 0.67704 | 2.152033 | 0 | 5.12E-04 |
| DNA REPAIR | 361 | 0.41548 | 2.150649 | 0 | 4.96E-04 |
| MATURATION OF SSU RRNA | 33 | 0.60758 | 2.140051 | 0 | 5.97E-04 |
| DNA REPLICATION | 164 | 0.452899 | 2.139244 | 0 | 6.08E-04 |
| NUCLEIC ACID PHOSPHODIESTER BOND HYDROLYSIS | 202 | 0.436466 | 2.125899 | 0 | 8.02E-04 |
| TRNA MODIFICATION | 47 | 0.553644 | 2.125761 | 0 | 7.80E-04 |
| RNA SPLICING | 287 | 0.426138 | 2.123179 | 0 | 7.59E-04 |
| DNA STRAND ELONGATION INVOLVED IN DNA REPLICATION | 23 | 0.666931 | 2.116285 | 0 | 8.37E-04 |
| DNA TEMPLATED TRANSCRIPTION ELONGATION | 78 | 0.495229 | 2.114468 | 0 | 8.64E-04 |
| NON RECOMBINATIONAL REPAIR | 43 | 0.57228 | 2.11254 | 0 | 8.89E-04 |
| ERROR PRONE TRANSLESION SYNTHESIS | 16 | 0.742897 | 2.107186 | 0 | 0.001004 |
| MICROTUBULE ORGANIZING CENTER ORGANIZATION | 67 | 0.51611 | 2.100217 | 0 | 0.001002 |
| PROTEIN TRANSMEMBRANE TRANSPORT | 43 | 0.56132 | 2.094941 | 0 | 0.001001 |
| CENTROSOME DUPLICATION | 26 | 0.632408 | 2.09088 | 0 | 0.001065 |
| MITOTIC RECOMBINATION | 36 | 0.590478 | 2.089188 | 0 | 0.001084 |
| MATURATION OF SSU RRNA FROM TRICISTRONIC RRNA TRANSCRIPT SSU RRNA 5 8S RRNA LSU RRNA | 28 | 0.60835 | 2.088791 | 0 | 0.001081 |
| TRANSCRIPTION FROM RNA POLYMERASE I PROMOTER | 30 | 0.605667 | 2.063864 | 0 | 0.001563 |
| MITOCHONDRION ORGANIZATION | 469 | 0.389861 | 2.063585 | 0 | 0.001551 |
| RIBOSOMAL SMALL SUBUNIT BIOGENESIS | 41 | 0.561527 | 2.040083 | 0 | 0.002216 |
| PROTEIN TARGETING TO MITOCHONDRION | 38 | 0.568699 | 2.03857 | 0 | 0.002229 |
| DNA GEOMETRIC CHANGE | 62 | 0.51362 | 2.037928 | 0 | 0.002187 |
| PTERIDINE CONTAINING COMPOUND METABOLIC PROCESS | 31 | 0.598299 | 2.026195 | 0 | 0.002688 |
| SNRNA PROCESSING | 18 | 0.691017 | 2.025354 | 0 | 0.002673 |
| MRNA PROCESSING | 347 | 0.390753 | 2.021955 | 0 | 0.002729 |
| RIBONUCLEOTIDE CATABOLIC PROCESS | 26 | 0.618648 | 2.021674 | 0 | 0.002697 |
| RNA DEPENDENT DNA BIOSYNTHETIC PROCESS | 19 | 0.67279 | 2.019105 | 0 | 0.002784 |
| NUCLEOBASE CONTAINING COMPOUND TRANSPORT | 160 | 0.426958 | 2.017472 | 0 | 0.002851 |
| DOUBLE STRAND BREAK REPAIR | 120 | 0.4464 | 2.0126 | 0 | 0.002966 |
| RNA LOCALIZATION | 147 | 0.434707 | 2.005663 | 0 | 0.003238 |
| NUCLEAR TRANSCRIBED MRNA CATABOLIC PROCESS EXONUCLEOLYTIC | 25 | 0.601577 | 2.002148 | 0.002488 | 0.003341 |
| MACROMOLECULAR COMPLEX DISASSEMBLY | 159 | 0.427685 | 1.994052 | 0 | 0.003817 |
| COFACTOR BIOSYNTHETIC PROCESS | 131 | 0.430488 | 1.992989 | 0 | 0.003819 |
| MITOTIC SISTER CHROMATID SEGREGATION | 69 | 0.485325 | 1.990519 | 0 | 0.003893 |
| MITOTIC NUCLEAR DIVISION | 283 | 0.39544 | 1.990436 | 0 | 0.003834 |
| AMIDE BIOSYNTHETIC PROCESS | 394 | 0.380251 | 1.985749 | 0 | 0.004066 |
| DNA DAMAGE RESPONSE DETECTION OF DNA DAMAGE | 34 | 0.565226 | 1.985462 | 0 | 0.00402 |
| NUCLEOTIDE EXCISION REPAIR PREINCISION COMPLEX ASSEMBLY | 24 | 0.627433 | 1.984462 | 0 | 0.003975 |
| MATURATION OF 5 8S RRNA | 25 | 0.591183 | 1.979144 | 0 | 0.004265 |
| NEGATIVE REGULATION OF PROTEIN MODIFICATION BY SMALL PROTEIN CONJUGATION OR REMOVAL | 120 | 0.435461 | 1.977925 | 0 | 0.00426 |
| NEGATIVE REGULATION OF VIRAL RELEASE FROM HOST CELL | 15 | 0.692587 | 1.97174 | 0 | 0.00462 |
| RNA METHYLATION | 39 | 0.543005 | 1.969674 | 0 | 0.004677 |
| TRANSCRIPTION ELONGATION FROM RNA POLYMERASE II PROMOTER | 65 | 0.490563 | 1.967555 | 0 | 0.004731 |
| NCRNA 3 END PROCESSING | 18 | 0.662523 | 1.966855 | 0 | 0.00468 |
| MITOCHONDRIAL RESPIRATORY CHAIN COMPLEX ASSEMBLY | 56 | 0.494883 | 1.963864 | 0 | 0.004746 |
| SISTER CHROMATID SEGREGATION | 135 | 0.432082 | 1.951075 | 0 | 0.005405 |
| CILIUM MORPHOGENESIS | 147 | 0.416516 | 1.93964 | 0 | 0.006109 |
| ANAPHASE PROMOTING COMPLEX DEPENDENT CATABOLIC PROCESS | 65 | 0.481907 | 1.938777 | 0.00277 | 0.006068 |
| NCRNA CATABOLIC PROCESS | 19 | 0.639921 | 1.93782 | 0 | 0.006088 |
| NONMOTILE PRIMARY CILIUM ASSEMBLY | 21 | 0.629855 | 1.936729 | 0.002326 | 0.006109 |
| TRNA TRANSPORT | 28 | 0.589273 | 1.936197 | 0 | 0.006057 |
| RIBOSOMAL LARGE SUBUNIT BIOGENESIS | 36 | 0.545452 | 1.933939 | 0 | 0.006148 |
| NUCLEOTIDE EXCISION REPAIR PREINCISION COMPLEX STABILIZATION | 17 | 0.645313 | 1.927754 | 0.002331 | 0.006514 |
| MRNA METABOLIC PROCESS | 476 | 0.363917 | 1.923003 | 0 | 0.006902 |
| CILIUM ORGANIZATION | 135 | 0.429565 | 1.922645 | 0 | 0.006889 |
| SPLICEOSOMAL COMPLEX ASSEMBLY | 42 | 0.520997 | 1.914696 | 0 | 0.007522 |
| RNA 3 END PROCESSING | 86 | 0.456832 | 1.91374 | 0 | 0.007535 |
| MITOCHONDRIAL RNA METABOLIC PROCESS | 26 | 0.578603 | 1.908249 | 0.002457 | 0.008201 |
| RNA PHOSPHODIESTER BOND HYDROLYSIS ENDONUCLEOLYTIC | 50 | 0.505149 | 1.907193 | 0 | 0.008249 |
| REGULATION OF TELOMERE MAINTENANCE VIA TELOMERE LENGTHENING | 41 | 0.51695 | 1.905924 | 0 | 0.008264 |
| FOLIC ACID CONTAINING COMPOUND METABOLIC PROCESS | 25 | 0.601401 | 1.904765 | 0 | 0.008331 |
| ERROR FREE TRANSLESION SYNTHESIS | 16 | 0.67984 | 1.904258 | 0 | 0.008282 |
| STRAND DISPLACEMENT | 20 | 0.613718 | 1.903767 | 0 | 0.008234 |
| PURINE CONTAINING COMPOUND CATABOLIC PROCESS | 43 | 0.50812 | 1.903259 | 0 | 0.008206 |
| PEROXISOME ORGANIZATION | 31 | 0.561151 | 1.900856 | 0.002353 | 0.008344 |
| PROTEIN UBIQUITINATION INVOLVED IN UBIQUITIN DEPENDENT PROTEIN CATABOLIC PROCESS | 103 | 0.432554 | 1.90014 | 0 | 0.008338 |
| DNA DEPENDENT DNA REPLICATION | 75 | 0.447963 | 1.899658 | 0 | 0.008311 |
| TELOMERE MAINTENANCE VIA TELOMERE LENGTHENING | 23 | 0.580468 | 1.899133 | 0.004474 | 0.008265 |
| PEPTIDYL LYSINE MODIFICATION | 240 | 0.386208 | 1.894993 | 0 | 0.008658 |
| TELOMERE MAINTENANCE VIA TELOMERASE | 16 | 0.657511 | 1.891147 | 0 | 0.008987 |
| MATURATION OF 5 8S RRNA FROM TRICISTRONIC RRNA TRANSCRIPT SSU RRNA 5 8S RRNA LSU RRNA | 19 | 0.620855 | 1.890155 | 0.002294 | 0.008992 |
| RNA PHOSPHODIESTER BOND HYDROLYSIS | 101 | 0.428005 | 1.888555 | 0 | 0.009054 |
| PROTEIN ACETYLATION | 97 | 0.427449 | 1.880749 | 0 | 0.009692 |
