# Supplementary material for: Transcriptomic changes triggered by ouabain in rat cerebellum granule cells: Role of α3- and α1-Na+,K+-ATPase-mediated signaling
Source: PLoS One. 2019 Sep 26;14(9):e0222767. doi: 10.1371/journal.pone.0222767 (PMC6762055; doi:10.1371/journal.pone.0222767)
Supplement: S5 Table — (DOCX) [file pone.0222767.s017.docx]

**Table S5. Upregulated gene sets (GeneOntology – Molecular Function) in 1mM ouabain-treated granular neurons significant at FDR < 1%.**

| **NAME** | **SIZE** | **ES** | **NES** | **NOM p-val** | **FDR q-val** |
| --- | --- | --- | --- | --- | --- |
| OLFACTORY RECEPTOR ACTIVITY | 254 | -0.78783 | -3.39699 | 0 | 0 |
| ODORANT BINDING | 65 | -0.78912 | -2.87298 | 0 | 0 |
| CYTOKINE ACTIVITY | 165 | -0.67492 | -2.80984 | 0 | 0 |
| SERINE TYPE ENDOPEPTIDASE INHIBITOR ACTIVITY | 67 | -0.73878 | -2.6832 | 0 | 0 |
| CYTOKINE RECEPTOR BINDING | 217 | -0.58571 | -2.51033 | 0 | 0 |
| GROWTH FACTOR ACTIVITY | 151 | -0.61575 | -2.50779 | 0 | 0 |
| PEPTIDASE INHIBITOR ACTIVITY | 122 | -0.62523 | -2.49405 | 0 | 0 |
| CHEMOKINE ACTIVITY | 33 | -0.78144 | -2.4858 | 0 | 0 |
| HORMONE ACTIVITY | 94 | -0.63773 | -2.46452 | 0 | 0 |
| CHEMOKINE RECEPTOR BINDING | 41 | -0.72892 | -2.43612 | 0 | 0 |
| GROWTH FACTOR RECEPTOR BINDING | 116 | -0.58224 | -2.29862 | 0 | 0 |
| PEPTIDASE REGULATOR ACTIVITY | 154 | -0.5475 | -2.25616 | 0 | 0 |
| TRANSCRIPTIONAL ACTIVATOR ACTIVITY RNA POLYMERASE II TRANSCRIPTION REGULATORY REGION SEQUENCE SPECIFIC BINDING | 280 | -0.51195 | -2.24291 | 0 | 0 |
| CCR CHEMOKINE RECEPTOR BINDING | 24 | -0.73418 | -2.17498 | 0 | 1.84E-04 |
| TRANSCRIPTIONAL ACTIVATOR ACTIVITY RNA POLYMERASE II CORE PROMOTER PROXIMAL REGION SEQUENCE SPECIFIC BINDING | 202 | -0.50991 | -2.16486 | 0 | 1.72E-04 |
| TRANSCRIPTION FACTOR ACTIVITY RNA POLYMERASE II CORE PROMOTER PROXIMAL REGION SEQUENCE SPECIFIC BINDING | 288 | -0.48046 | -2.10624 | 0 | 3.15E-04 |
| TASTE RECEPTOR ACTIVITY | 19 | -0.75304 | -2.08065 | 0 | 5.34E-04 |
| ENZYME INHIBITOR ACTIVITY | 284 | -0.47006 | -2.05973 | 0 | 8.69E-04 |
| SERINE HYDROLASE ACTIVITY | 179 | -0.48896 | -2.05595 | 0 | 9.14E-04 |
| NEUROPEPTIDE RECEPTOR BINDING | 25 | -0.67698 | -2.0486 | 0 | 9.08E-04 |
| BITTER TASTE RECEPTOR ACTIVITY | 15 | -0.77175 | -2.03341 | 0 | 0.001096 |
| G PROTEIN COUPLED AMINE RECEPTOR ACTIVITY | 43 | -0.59572 | -1.99305 | 0 | 0.002009 |
| CORE PROMOTER PROXIMAL REGION DNA BINDING | 278 | -0.45012 | -1.97008 | 0 | 0.002638 |
| HEPARIN BINDING | 123 | -0.47861 | -1.93202 | 0 | 0.004435 |
| NEUROPEPTIDE HORMONE ACTIVITY | 25 | -0.64726 | -1.93169 | 0.001748 | 0.004257 |
| CHEMOATTRACTANT ACTIVITY | 20 | -0.67591 | -1.92136 | 0 | 0.004814 |
| METALLOCARBOXYPEPTIDASE ACTIVITY | 21 | -0.65977 | -1.91249 | 0 | 0.005113 |
| RNA POLYMERASE II ACTIVATING TRANSCRIPTION FACTOR BINDING | 31 | -0.60524 | -1.89547 | 0.001661 | 0.006481 |
| G PROTEIN COUPLED RECEPTOR BINDING | 212 | -0.44272 | -1.89444 | 0 | 0.006313 |
| AMMONIUM TRANSMEMBRANE TRANSPORTER ACTIVITY | 19 | -0.67299 | -1.87562 | 0.001754 | 0.007842 |
| PEPTIDE RECEPTOR ACTIVITY | 114 | -0.46854 | -1.86013 | 0 | 0.008908 |
| TRANSCRIPTIONAL REPRESSOR ACTIVITY RNA POLYMERASE II TRANSCRIPTION REGULATORY REGION SEQUENCE SPECIFIC BINDING | 143 | -0.4493 | -1.85647 | 0 | 0.008986 |
