# Supplementary material for: Transcriptomic changes triggered by ouabain in rat cerebellum granule cells: Role of α3- and α1-Na+,K+-ATPase-mediated signaling
Source: PLoS One. 2019 Sep 26;14(9):e0222767. doi: 10.1371/journal.pone.0222767 (PMC6762055; doi:10.1371/journal.pone.0222767)
Supplement: S6 Table — (DOCX) [file pone.0222767.s018.docx]

**Table S6. Downregulated gene sets (GeneOntology – Molecular Function) in 1mM ouabain-treated granular neurons significant at FDR < 1%.**

| **NAME** | **SIZE** | **ES** | **NES** | **NOM p-val** | **FDR q-val** |
| --- | --- | --- | --- | --- | --- |
| LIGASE ACTIVITY FORMING CARBON OXYGEN BONDS | 38 | 0.646618 | 2.349837 | 0 | 0.001333 |
| NUCLEOTIDYLTRANSFERASE ACTIVITY | 105 | 0.48574 | 2.15403 | 0 | 0.001358 |
| ENDORIBONUCLEASE ACTIVITY PRODUCING 5 PHOSPHOMONOESTERS | 24 | 0.648268 | 2.112124 | 0 | 0.002607 |
| UBIQUITIN LIKE PROTEIN BINDING | 100 | 0.475267 | 2.107704 | 0 | 0.001955 |
| TRNA BINDING | 36 | 0.595884 | 2.102997 | 0 | 0.0018 |
| ENDONUCLEASE ACTIVITY ACTIVE WITH EITHER RIBO OR DEOXYRIBONUCLEIC ACIDS AND PRODUCING 5 PHOSPHOMONOESTERS | 29 | 0.628701 | 2.099893 | 0 | 0.0015 |
| DAMAGED DNA BINDING | 57 | 0.526313 | 2.068193 | 0 | 0.002571 |
| PURINE NTP DEPENDENT HELICASE ACTIVITY | 69 | 0.504849 | 2.05641 | 0 | 0.002869 |
| DNA HELICASE ACTIVITY | 40 | 0.566178 | 2.055326 | 0 | 0.002707 |
| SNORNA BINDING | 24 | 0.63125 | 2.043829 | 0 | 0.003086 |
| RNA POLYMERASE ACTIVITY | 35 | 0.578073 | 2.030861 | 0 | 0.003407 |
| INTRAMOLECULAR TRANSFERASE ACTIVITY | 23 | 0.645477 | 2.008386 | 0 | 0.004836 |
| HELICASE ACTIVITY | 110 | 0.444331 | 1.996572 | 0 | 0.005584 |
| RNA METHYLTRANSFERASE ACTIVITY | 33 | 0.571829 | 1.957154 | 0 | 0.008335 |
| ATP DEPENDENT DNA HELICASE ACTIVITY | 25 | 0.595029 | 1.948409 | 0 | 0.008562 |
| RRNA BINDING | 48 | 0.499027 | 1.927377 | 0 | 0.009779 |
