# Supplementary material for: Transcriptomic changes triggered by ouabain in rat cerebellum granule cells: Role of α3- and α1-Na+,K+-ATPase-mediated signaling
Source: PLoS One. 2019 Sep 26;14(9):e0222767. doi: 10.1371/journal.pone.0222767 (PMC6762055; doi:10.1371/journal.pone.0222767)
Supplement: S7 Table — (DOCX) [file pone.0222767.s019.docx]

**Table S7. Upregulated gene sets (GeneOntology – Cellular Component) in 1mM ouabain-treated granular neurons significant at FDR < 1%.**

| **NAME** | **SIZE** | **ES** | **NES** | **NOM p-val** | **FDR q-val** |
| --- | --- | --- | --- | --- | --- |
| EXTERNAL SIDE OF PLASMA MEMBRANE | 178 | -0.51719 | -2.17342 | 0 | 0 |
