# Supplementary material for: Transcriptomic changes triggered by ouabain in rat cerebellum granule cells: Role of α3- and α1-Na+,K+-ATPase-mediated signaling
Source: PLoS One. 2019 Sep 26;14(9):e0222767. doi: 10.1371/journal.pone.0222767 (PMC6762055; doi:10.1371/journal.pone.0222767)
Supplement: S8 Table — (DOCX) [file pone.0222767.s020.docx]

**Table S8. Downregulated gene sets (GeneOntology – Cellular Component) in 1mM ouabain-treated granular neurons significant at FDR < 1%.**

| **NAME** | **SIZE** | **ES** | **NES** | **NOM p-val** | **FDR q-val** |
| --- | --- | --- | --- | --- | --- |
| MITOCHONDRIAL MATRIX | 352 | 0.462934 | 2.417649 | 0 | 0 |
| PRERIBOSOME | 46 | 0.635679 | 2.374016 | 0 | 0 |
| NUCLEOID | 40 | 0.639275 | 2.357507 | 0 | 0 |
| CENTROSOME | 380 | 0.431319 | 2.225648 | 0 | 0 |
| ORGANELLAR RIBOSOME | 63 | 0.559033 | 2.218964 | 0 | 0 |
| MICROTUBULE ORGANIZING CENTER | 485 | 0.414164 | 2.218154 | 0 | 0 |
| ACETYLTRANSFERASE COMPLEX | 69 | 0.545295 | 2.206129 | 0 | 4.79E-04 |
| NUCLEOLAR PART | 51 | 0.566722 | 2.163123 | 0 | 8.84E-04 |
