# Supplementary material for: Transcriptomic changes triggered by ouabain in rat cerebellum granule cells: Role of α3- and α1-Na+,K+-ATPase-mediated signaling
Source: PLoS One. 2019 Sep 26;14(9):e0222767. doi: 10.1371/journal.pone.0222767 (PMC6762055; doi:10.1371/journal.pone.0222767)
Supplement: S9 Table — (DOCX) [file pone.0222767.s021.docx]

**Table S9. Upregulated gene sets (GeneOntology – Biological Process) in 100nM ouabain-treated granular neurons at NES < -1.35.**

| **NAME** | **SIZE** | **ES** | **NES** | **NOM p-val** | **FDR q-val** |
| --- | --- | --- | --- | --- | --- |
| ENTRAINMENT OF CIRCADIAN CLOCK BY PHOTOPERIOD | 16 | -0.78993 | -2.12833 | 0 | 0.029364 |
| POSITIVE REGULATION OF FAT CELL DIFFERENTIATION | 38 | -0.58573 | -1.98148 | 0 | 0.183615 |
| REGULATION OF CELL CYCLE ARREST | 87 | -0.50989 | -1.96171 | 0 | 0.156726 |
| LUNG MORPHOGENESIS | 37 | -0.58713 | -1.92298 | 0 | 0.199341 |
| REGULATION OF PRI MIRNA TRANSCRIPTION FROM RNA POLYMERASE II PROMOTER | 16 | -0.70312 | -1.91282 | 0 | 0.179494 |
| B CELL RECEPTOR SIGNALING PATHWAY | 30 | -0.59554 | -1.90206 | 0 | 0.173668 |
| POSITIVE REGULATION OF CELL CYCLE ARREST | 68 | -0.50517 | -1.8768 | 0 | 0.21001 |
| EPITHELIAL TUBE BRANCHING INVOLVED IN LUNG MORPHOGENESIS | 18 | -0.66808 | -1.8659 | 0.002217 | 0.213571 |
| NEGATIVE REGULATION OF SIGNAL TRANSDUCTION IN ABSENCE OF LIGAND | 26 | -0.61903 | -1.86281 | 0.004357 | 0.198425 |
| REGULATION OF DNA DAMAGE RESPONSE SIGNAL TRANSDUCTION BY P53 CLASS MEDIATOR | 23 | -0.63303 | -1.83562 | 0 | 0.248551 |
| G1 DNA DAMAGE CHECKPOINT | 54 | -0.50962 | -1.82389 | 0 | 0.255569 |
| ENTRAINMENT OF CIRCADIAN CLOCK | 22 | -0.62925 | -1.81435 | 0.006522 | 0.263273 |
| CENTROMERE COMPLEX ASSEMBLY | 24 | -0.61936 | -1.81225 | 0.002309 | 0.248568 |
| FAT CELL DIFFERENTIATION | 94 | -0.46191 | -1.79948 | 0 | 0.262103 |
| REGULATION OF FAT CELL DIFFERENTIATION | 88 | -0.47043 | -1.7957 | 0 | 0.255915 |
| NEGATIVE REGULATION OF MAP KINASE ACTIVITY | 60 | -0.49188 | -1.77516 | 0 | 0.297092 |
| REGULATION OF LYMPHOCYTE DIFFERENTIATION | 112 | -0.43754 | -1.77362 | 0 | 0.284332 |
| MAST CELL MEDIATED IMMUNITY | 15 | -0.67582 | -1.77283 | 0.002137 | 0.271067 |
| NEGATIVE REGULATION OF LEUKOCYTE DIFFERENTIATION | 65 | -0.48113 | -1.77059 | 0 | 0.26363 |
| REGULATION OF B CELL DIFFERENTIATION | 20 | -0.62231 | -1.76929 | 0.006565 | 0.253668 |
| REGULATION OF CARBOHYDRATE BIOSYNTHETIC PROCESS | 77 | -0.46592 | -1.76462 | 0 | 0.253945 |
| SIGNAL TRANSDUCTION IN RESPONSE TO DNA DAMAGE | 78 | -0.46493 | -1.76453 | 0 | 0.242641 |
| REGULATION OF ANOIKIS | 19 | -0.63169 | -1.76062 | 0.006479 | 0.24302 |
| LENS MORPHOGENESIS IN CAMERA TYPE EYE | 17 | -0.63003 | -1.7585 | 0.006682 | 0.237554 |
| INACTIVATION OF MAPK ACTIVITY | 23 | -0.60885 | -1.75842 | 0.008529 | 0.228266 |
| PHOTOPERIODISM | 21 | -0.60837 | -1.75377 | 0.006928 | 0.229219 |
| MITOTIC DNA INTEGRITY CHECKPOINT | 73 | -0.45954 | -1.7536 | 0.002591 | 0.221124 |
| CELLULAR RESPONSE TO FLUID SHEAR STRESS | 17 | -0.62443 | -1.72358 | 0.009324 | 0.281468 |
| CELL ACTIVATION INVOLVED IN IMMUNE RESPONSE | 106 | -0.43664 | -1.71806 | 0 | 0.28647 |
| PATTERN RECOGNITION RECEPTOR SIGNALING PATHWAY | 87 | -0.4466 | -1.71757 | 0 | 0.277905 |
| HISTONE EXCHANGE | 26 | -0.56465 | -1.71367 | 0.008929 | 0.278679 |
| REGULATION OF T HELPER CELL DIFFERENTIATION | 22 | -0.5921 | -1.71288 | 0.008909 | 0.272512 |
| REGULATION OF ALPHA BETA T CELL DIFFERENTIATION | 40 | -0.50786 | -1.70842 | 0.006608 | 0.275754 |
| NEGATIVE REGULATION OF CELL CYCLE G1 S PHASE TRANSITION | 74 | -0.45779 | -1.70088 | 0.005089 | 0.288324 |
| MITOTIC CELL CYCLE CHECKPOINT | 104 | -0.41975 | -1.70078 | 0 | 0.280198 |
| SULFUR COMPOUND CATABOLIC PROCESS | 36 | -0.51233 | -1.70073 | 0.011848 | 0.272563 |
| MYD88 DEPENDENT TOLL LIKE RECEPTOR SIGNALING PATHWAY | 23 | -0.58789 | -1.69122 | 0.020316 | 0.289306 |
| POSITIVE REGULATION OF B CELL ACTIVATION | 59 | -0.47518 | -1.69069 | 0.002237 | 0.283058 |
| NEGATIVE REGULATION OF PROTEIN SERINE THREONINE KINASE ACTIVITY | 105 | -0.42753 | -1.68589 | 0.002571 | 0.288867 |
| REGULATION OF EXTRINSIC APOPTOTIC SIGNALING PATHWAY IN ABSENCE OF LIGAND | 39 | -0.51009 | -1.68472 | 0.009281 | 0.284718 |
| ACTIVATION OF INNATE IMMUNE RESPONSE | 164 | -0.39615 | -1.68329 | 0 | 0.281455 |
| MAST CELL ACTIVATION | 17 | -0.6227 | -1.68179 | 0.011416 | 0.278234 |
| NEGATIVE REGULATION OF LYMPHOCYTE DIFFERENTIATION | 31 | -0.53125 | -1.68071 | 0.002336 | 0.274327 |
| SPROUTING ANGIOGENESIS | 39 | -0.51258 | -1.68039 | 0 | 0.268663 |
| DNA INTEGRITY CHECKPOINT | 113 | -0.41084 | -1.67682 | 0 | 0.27145 |
| POSITIVE REGULATION OF B CELL PROLIFERATION | 34 | -0.51917 | -1.67632 | 0.015625 | 0.266507 |
| REGULATION OF CELL MATURATION | 15 | -0.62894 | -1.67559 | 0.006742 | 0.262728 |
| MYELOID CELL ACTIVATION INVOLVED IN IMMUNE RESPONSE | 35 | -0.51818 | -1.67069 | 0 | 0.268583 |
| MYELOID LEUKOCYTE ACTIVATION | 81 | -0.43483 | -1.66531 | 0 | 0.275589 |
| REGULATION OF GLUCONEOGENESIS | 32 | -0.52297 | -1.66497 | 0.011161 | 0.271121 |
| DNA DAMAGE RESPONSE DETECTION OF DNA DAMAGE | 34 | -0.52053 | -1.66438 | 0.015801 | 0.266961 |
| NEGATIVE REGULATION OF DNA BIOSYNTHETIC PROCESS | 25 | -0.54376 | -1.66375 | 0.004808 | 0.263546 |
| REGULATION OF B CELL ACTIVATION | 88 | -0.42367 | -1.66105 | 0.005115 | 0.265704 |
| MYELOID DENDRITIC CELL ACTIVATION | 21 | -0.58455 | -1.65996 | 0.020501 | 0.263263 |
| POSITIVE REGULATION OF LYMPHOCYTE DIFFERENTIATION | 70 | -0.43713 | -1.65961 | 0.004854 | 0.259322 |
| DNA REPLICATION INDEPENDENT NUCLEOSOME ORGANIZATION | 28 | -0.53082 | -1.64463 | 0.014118 | 0.291504 |
| RESPONSE TO FOLIC ACID | 15 | -0.61908 | -1.63829 | 0.024176 | 0.301449 |
| TOLL LIKE RECEPTOR SIGNALING PATHWAY | 70 | -0.43653 | -1.63814 | 0.004505 | 0.296481 |
| REGULATION OF B CELL PROLIFERATION | 49 | -0.47073 | -1.63635 | 0.007092 | 0.295973 |
| B CELL ACTIVATION | 102 | -0.41417 | -1.6334 | 0.004651 | 0.299326 |
| CELLULAR RESPONSE TO PEPTIDE | 236 | -0.36839 | -1.62802 | 0 | 0.309099 |
| CELL CYCLE CHECKPOINT | 152 | -0.38948 | -1.6272 | 0 | 0.306047 |
| REGULATION OF MAST CELL ACTIVATION | 33 | -0.51872 | -1.62593 | 0.010395 | 0.30468 |
| FUCOSYLATION | 16 | -0.59709 | -1.62318 | 0.017738 | 0.306689 |
| MATURE B CELL DIFFERENTIATION | 17 | -0.59776 | -1.61733 | 0.022624 | 0.316538 |
| REGULATION OF T CELL DIFFERENTIATION | 91 | -0.41847 | -1.61344 | 0 | 0.322385 |
| ACTIVATION OF IMMUNE RESPONSE | 313 | -0.3489 | -1.61252 | 0 | 0.320301 |
| NEGATIVE REGULATION OF MAPK CASCADE | 123 | -0.39209 | -1.61001 | 0.002695 | 0.322571 |
| LEUKOCYTE DEGRANULATION | 26 | -0.52962 | -1.6098 | 0.01559 | 0.318357 |
| REGULATION OF FIBROBLAST GROWTH FACTOR RECEPTOR SIGNALING PATHWAY | 21 | -0.56364 | -1.60895 | 0.029279 | 0.316235 |
| REGULATION OF DNA BINDING | 74 | -0.42731 | -1.60723 | 0.004773 | 0.316127 |
| NEGATIVE REGULATION OF HEMOPOIESIS | 95 | -0.40666 | -1.60467 | 0 | 0.318336 |
| NEGATIVE REGULATION OF LYMPHOCYTE APOPTOTIC PROCESS | 23 | -0.54498 | -1.60457 | 0.021645 | 0.314103 |
| REGULATION OF SMOOTH MUSCLE CELL PROLIFERATION | 94 | -0.40594 | -1.60295 | 0 | 0.314303 |
| REGULATION OF GLUCOSE METABOLIC PROCESS | 89 | -0.40068 | -1.59973 | 0.002639 | 0.3181 |
| NEGATIVE REGULATION OF T CELL DIFFERENTIATION | 25 | -0.5387 | -1.59694 | 0.015873 | 0.321257 |
| LYSOSOME LOCALIZATION | 19 | -0.5739 | -1.59675 | 0.020045 | 0.317655 |
| MACROPHAGE ACTIVATION | 26 | -0.50941 | -1.59657 | 0.021429 | 0.314027 |
| MAMMARY GLAND MORPHOGENESIS | 39 | -0.47186 | -1.59298 | 0.014151 | 0.318399 |
| POSITIVE REGULATION OF VASCULAR ENDOTHELIAL GROWTH FACTOR PRODUCTION | 24 | -0.53317 | -1.59187 | 0.016241 | 0.317319 |
| NEGATIVE REGULATION OF DNA BINDING | 37 | -0.48051 | -1.58865 | 0.011655 | 0.321367 |
| NEGATIVE REGULATION OF CELL CYCLE ARREST | 16 | -0.60225 | -1.58854 | 0.021692 | 0.317776 |
| POSITIVE REGULATION OF SMOOTH MUSCLE CELL PROLIFERATION | 57 | -0.444 | -1.58389 | 0.013575 | 0.325982 |
| ATP DEPENDENT CHROMATIN REMODELING | 44 | -0.47015 | -1.58304 | 0.018223 | 0.324578 |
| REGULATION OF LEUKOCYTE DIFFERENTIATION | 197 | -0.36274 | -1.58096 | 0 | 0.326047 |
| POSITIVE REGULATION OF MAST CELL ACTIVATION | 16 | -0.59738 | -1.58028 | 0.030108 | 0.32437 |
| RESPONSE TO PEPTIDE | 356 | -0.34087 | -1.57894 | 0 | 0.32398 |
| POSITIVE REGULATION OF MYELOID LEUKOCYTE MEDIATED IMMUNITY | 18 | -0.57738 | -1.57881 | 0.030043 | 0.32068 |
| POSITIVE REGULATION OF CHEMOKINE PRODUCTION | 44 | -0.46867 | -1.57661 | 0.011521 | 0.322799 |
| ENDOCHONDRAL BONE MORPHOGENESIS | 39 | -0.47784 | -1.57479 | 0.015 | 0.32364 |
| BLOOD VESSEL ENDOTHELIAL CELL MIGRATION | 21 | -0.55548 | -1.57408 | 0.024609 | 0.321952 |
| REPLACEMENT OSSIFICATION | 25 | -0.52283 | -1.57077 | 0.01573 | 0.326868 |
| NEGATIVE REGULATION OF MYELOID LEUKOCYTE DIFFERENTIATION | 36 | -0.48572 | -1.56986 | 0.018957 | 0.3257 |
| POSITIVE REGULATION OF INTERLEUKIN 2 PRODUCTION | 29 | -0.51501 | -1.56575 | 0.021505 | 0.333012 |
| B CELL DIFFERENTIATION | 66 | -0.42035 | -1.56077 | 0.018913 | 0.342037 |
| REGULATION OF INTERLEUKIN 2 PRODUCTION | 44 | -0.45921 | -1.55718 | 0.011655 | 0.348548 |
| NEGATIVE REGULATION OF STEROID METABOLIC PROCESS | 18 | -0.55701 | -1.55422 | 0.033898 | 0.352733 |
| REGULATION OF ACTIN CYTOSKELETON REORGANIZATION | 30 | -0.49889 | -1.55145 | 0.022272 | 0.356507 |
| REGULATION OF CARBOHYDRATE METABOLIC PROCESS | 142 | -0.36781 | -1.55144 | 0 | 0.352906 |
| REGULATION OF CD4 POSITIVE ALPHA BETA T CELL ACTIVATION | 32 | -0.48627 | -1.55043 | 0.021028 | 0.352125 |
| SIGNAL TRANSDUCTION BY P53 CLASS MEDIATOR | 103 | -0.38684 | -1.549 | 0.002315 | 0.352415 |
| NEUROBLAST PROLIFERATION | 25 | -0.51907 | -1.54683 | 0.022676 | 0.354994 |
| POSITIVE REGULATION OF CARBOHYDRATE METABOLIC PROCESS | 67 | -0.42014 | -1.54529 | 0.0075 | 0.355908 |
| NEGATIVE REGULATION OF CELL CYCLE PROCESS | 168 | -0.36273 | -1.54354 | 0 | 0.35709 |
| NEGATIVE REGULATION OF KINASE ACTIVITY | 217 | -0.35241 | -1.54049 | 0 | 0.361132 |
| G PROTEIN COUPLED PURINERGIC RECEPTOR SIGNALING PATHWAY | 18 | -0.55586 | -1.5402 | 0.052632 | 0.35858 |
| ENDOTHELIAL CELL MIGRATION | 45 | -0.44707 | -1.54019 | 0.018561 | 0.355241 |
| REGULATION OF FATTY ACID OXIDATION | 28 | -0.5098 | -1.53962 | 0.031963 | 0.353149 |
| NEGATIVE REGULATION OF T CELL APOPTOTIC PROCESS | 15 | -0.59618 | -1.53289 | 0.032895 | 0.367279 |
| EMBRYONIC EYE MORPHOGENESIS | 30 | -0.48679 | -1.53111 | 0.011574 | 0.368914 |
| REGULATION OF ALPHA BETA T CELL ACTIVATION | 58 | -0.42814 | -1.53094 | 0.009217 | 0.365926 |
| REGULATION OF GRANULOCYTE CHEMOTAXIS | 30 | -0.48164 | -1.53033 | 0.024775 | 0.364148 |
| CELLULAR RESPONSE TO INTERLEUKIN 6 | 20 | -0.5497 | -1.52816 | 0.048117 | 0.366931 |
| REGULATION OF RUFFLE ASSEMBLY | 19 | -0.54331 | -1.52736 | 0.042056 | 0.365595 |
| NEGATIVE REGULATION OF MITOTIC CELL CYCLE | 149 | -0.36212 | -1.5267 | 0.002747 | 0.364459 |
| FC RECEPTOR SIGNALING PATHWAY | 161 | -0.3548 | -1.51581 | 0 | 0.390304 |
| POSITIVE T CELL SELECTION | 19 | -0.53779 | -1.51533 | 0.064073 | 0.388291 |
| REGULATION OF ACUTE INFLAMMATORY RESPONSE | 64 | -0.42071 | -1.5143 | 0.005168 | 0.387743 |
| NEGATIVE REGULATION OF CARBOHYDRATE METABOLIC PROCESS | 38 | -0.46826 | -1.51186 | 0.036446 | 0.390815 |
| RETINOIC ACID RECEPTOR SIGNALING PATHWAY | 17 | -0.55988 | -1.5109 | 0.051836 | 0.390316 |
| REGULATION OF CELL CYCLE G1 S PHASE TRANSITION | 115 | -0.37967 | -1.51085 | 0.005263 | 0.38719 |
| MYELOID DENDRITIC CELL DIFFERENTIATION | 17 | -0.55125 | -1.51082 | 0.050439 | 0.384116 |
| STEM CELL PROLIFERATION | 54 | -0.41872 | -1.50923 | 0.016393 | 0.385274 |
| BRANCHING MORPHOGENESIS OF AN EPITHELIAL TUBE | 111 | -0.37711 | -1.5091 | 0.002538 | 0.382521 |
| NEGATIVE REGULATION OF PHOSPHORYLATION | 361 | -0.32371 | -1.50716 | 0 | 0.384896 |
| OSTEOBLAST DEVELOPMENT | 17 | -0.56654 | -1.5068 | 0.049145 | 0.38268 |
| INTRACELLULAR RECEPTOR SIGNALING PATHWAY | 145 | -0.3588 | -1.50425 | 0.007732 | 0.386784 |
| REGULATION OF MUSCLE ADAPTATION | 59 | -0.41557 | -1.50384 | 0.013187 | 0.384834 |
| RESPONSE TO IMMOBILIZATION STRESS | 18 | -0.54321 | -1.50159 | 0.06865 | 0.388135 |
| REGULATION OF FATTY ACID TRANSPORT | 26 | -0.49257 | -1.4999 | 0.03066 | 0.390193 |
| REGULATION OF TRANSCRIPTION INVOLVED IN G1 S TRANSITION OF MITOTIC CELL CYCLE | 23 | -0.51637 | -1.49471 | 0.046358 | 0.401145 |
| REGULATION OF HEMOPOIESIS | 256 | -0.33365 | -1.49151 | 0.002941 | 0.407253 |
| POSITIVE REGULATION OF T HELPER CELL DIFFERENTIATION | 16 | -0.55678 | -1.49111 | 0.055066 | 0.405382 |
| CARBOHYDRATE HOMEOSTASIS | 141 | -0.35426 | -1.48965 | 0.007792 | 0.406133 |
| HUMORAL IMMUNE RESPONSE | 100 | -0.37695 | -1.48878 | 0.009804 | 0.405595 |
| NEGATIVE REGULATION OF DNA REPLICATION | 40 | -0.45123 | -1.48796 | 0.036697 | 0.404749 |
| RENAL SYSTEM VASCULATURE DEVELOPMENT | 15 | -0.56988 | -1.48778 | 0.058315 | 0.402339 |
| NEGATIVE REGULATION OF CELL CYCLE PHASE TRANSITION | 115 | -0.36498 | -1.48711 | 0.012723 | 0.401383 |
| REGULATION OF SULFUR METABOLIC PROCESS | 20 | -0.53462 | -1.48672 | 0.051919 | 0.399531 |
| INSULIN RECEPTOR SIGNALING PATHWAY | 70 | -0.40126 | -1.48582 | 0.019851 | 0.399192 |
| NEGATIVE REGULATION OF TRANSFERASE ACTIVITY | 295 | -0.32173 | -1.48458 | 0 | 0.399524 |
| CELLULAR RESPONSE TO MECHANICAL STIMULUS | 72 | -0.3882 | -1.48057 | 0.012136 | 0.407922 |
| RESPONSE TO MOLECULE OF BACTERIAL ORIGIN | 282 | -0.32454 | -1.47545 | 0 | 0.420136 |
| POSITIVE REGULATION OF NEURON APOPTOTIC PROCESS | 43 | -0.43422 | -1.47348 | 0.026253 | 0.422827 |
| POSITIVE REGULATION OF GLUCOSE METABOLIC PROCESS | 31 | -0.47776 | -1.47331 | 0.03972 | 0.420335 |
| MAMMARY GLAND DEVELOPMENT | 111 | -0.36198 | -1.47246 | 0.010127 | 0.420072 |
| REGULATION OF TRANSCRIPTION REGULATORY REGION DNA BINDING | 30 | -0.47382 | -1.46974 | 0.044393 | 0.424967 |
| ESTABLISHMENT OF MITOTIC SPINDLE LOCALIZATION | 23 | -0.50474 | -1.46927 | 0.040773 | 0.423386 |
| FC EPSILON RECEPTOR SIGNALING PATHWAY | 110 | -0.36836 | -1.46788 | 0.010101 | 0.424169 |
| CELL SURFACE RECEPTOR SIGNALING PATHWAY INVOLVED IN HEART DEVELOPMENT | 15 | -0.54673 | -1.46573 | 0.062762 | 0.427404 |
| NEGATIVE REGULATION OF SMOOTH MUSCLE CELL PROLIFERATION | 34 | -0.45444 | -1.46381 | 0.048998 | 0.430247 |
| POSITIVE REGULATION OF IMMUNE RESPONSE | 429 | -0.3081 | -1.4629 | 0 | 0.430067 |
| REGULATION OF GLUCOSE TRANSPORT | 86 | -0.37672 | -1.46146 | 0.017241 | 0.431284 |
| REGULATION OF LEUKOCYTE PROLIFERATION | 174 | -0.34282 | -1.46111 | 0.00274 | 0.429763 |
| NEGATIVE REGULATION OF FIBROBLAST PROLIFERATION | 22 | -0.50312 | -1.46073 | 0.040089 | 0.427867 |
| CELLULAR RESPONSE TO BIOTIC STIMULUS | 146 | -0.34808 | -1.45926 | 0.005236 | 0.429346 |
| MEMBRANE DISASSEMBLY | 40 | -0.43715 | -1.45608 | 0.042155 | 0.435462 |
| STEROID HORMONE MEDIATED SIGNALING PATHWAY | 109 | -0.36089 | -1.45603 | 0.007481 | 0.432841 |
| REGULATION OF PROTEIN SERINE THREONINE KINASE ACTIVITY | 398 | -0.31209 | -1.45592 | 0 | 0.430397 |
| TRANSCRIPTION INITIATION FROM RNA POLYMERASE II PROMOTER | 124 | -0.35273 | -1.45257 | 0.004975 | 0.438015 |
| IMMUNE RESPONSE REGULATING CELL SURFACE RECEPTOR SIGNALING PATHWAY | 235 | -0.32338 | -1.45247 | 0 | 0.435648 |
| LYMPH VESSEL DEVELOPMENT | 17 | -0.52228 | -1.45131 | 0.067245 | 0.436427 |
| REGULATION OF INTERLEUKIN 2 BIOSYNTHETIC PROCESS | 16 | -0.54835 | -1.44923 | 0.075922 | 0.440117 |
| NEGATIVE REGULATION OF NF KAPPAB TRANSCRIPTION FACTOR ACTIVITY | 46 | -0.42054 | -1.44801 | 0.040449 | 0.441179 |
| NEGATIVE REGULATION OF ALCOHOL BIOSYNTHETIC PROCESS | 15 | -0.53513 | -1.44698 | 0.070064 | 0.441466 |
| VENTRICULAR CARDIAC MUSCLE CELL DIFFERENTIATION | 18 | -0.53123 | -1.44662 | 0.078431 | 0.439965 |
| INNATE IMMUNE RESPONSE | 410 | -0.30606 | -1.44623 | 0 | 0.438399 |
| RESPONSE TO FLUID SHEAR STRESS | 32 | -0.46425 | -1.4459 | 0.057471 | 0.436813 |
| POSITIVE REGULATION OF GLUCOSE TRANSPORT | 38 | -0.43538 | -1.44333 | 0.026128 | 0.441628 |
| IMMUNE EFFECTOR PROCESS | 338 | -0.31258 | -1.44248 | 0 | 0.441599 |
| REGULATION OF UBIQUITIN PROTEIN LIGASE ACTIVITY | 15 | -0.54985 | -1.44225 | 0.071264 | 0.439844 |
| POST EMBRYONIC DEVELOPMENT | 72 | -0.39017 | -1.44219 | 0.03066 | 0.437512 |
| REGULATION OF EXTRINSIC APOPTOTIC SIGNALING PATHWAY | 133 | -0.34744 | -1.442 | 0.014354 | 0.435501 |
| NEGATIVE REGULATION OF RESPONSE TO DNA DAMAGE STIMULUS | 44 | -0.42581 | -1.44171 | 0.038902 | 0.433774 |
| REGULATION OF CARDIAC MUSCLE CELL CONTRACTION | 22 | -0.49712 | -1.43971 | 0.056054 | 0.437595 |
| REGULATION OF CELL CYCLE CHECKPOINT | 23 | -0.48478 | -1.43925 | 0.064854 | 0.436489 |
| POSITIVE REGULATION OF REACTIVE OXYGEN SPECIES METABOLIC PROCESS | 77 | -0.37606 | -1.43637 | 0.023256 | 0.441993 |
| REGULATION OF ACTIVATED T CELL PROLIFERATION | 31 | -0.46198 | -1.43606 | 0.075893 | 0.440336 |
| NEGATIVE REGULATION OF RESPONSE TO REACTIVE OXYGEN SPECIES | 17 | -0.53623 | -1.43531 | 0.068966 | 0.440177 |
| POSITIVE REGULATION OF CELL ACTIVATION | 251 | -0.32138 | -1.43478 | 0.002976 | 0.439422 |
| ORGAN REGENERATION | 76 | -0.37829 | -1.43321 | 0.021176 | 0.441109 |
| POSITIVE REGULATION OF FATTY ACID METABOLIC PROCESS | 31 | -0.45281 | -1.4331 | 0.068127 | 0.439091 |
| GLIAL CELL MIGRATION | 29 | -0.45188 | -1.43306 | 0.0625 | 0.436851 |
| REGULATION OF REACTIVE OXYGEN SPECIES BIOSYNTHETIC PROCESS | 58 | -0.40152 | -1.43291 | 0.038554 | 0.434855 |
| REGULATION OF MAST CELL ACTIVATION INVOLVED IN IMMUNE RESPONSE | 26 | -0.48549 | -1.43155 | 0.049412 | 0.436449 |
| POSITIVE REGULATION OF RESPONSE TO BIOTIC STIMULUS | 31 | -0.45159 | -1.43092 | 0.058005 | 0.435927 |
| REGULATION OF OSSIFICATION | 158 | -0.33847 | -1.43082 | 0.00813 | 0.433924 |
| INSULIN SECRETION | 34 | -0.43909 | -1.43072 | 0.063492 | 0.431921 |
| NEGATIVE REGULATION OF MYELOID CELL DIFFERENTIATION | 60 | -0.39422 | -1.42832 | 0.028504 | 0.436706 |
| NEGATIVE REGULATION OF RESPONSE TO OXIDATIVE STRESS | 31 | -0.45287 | -1.42825 | 0.059603 | 0.434654 |
| AMINOGLYCAN CATABOLIC PROCESS | 55 | -0.40365 | -1.42761 | 0.045558 | 0.434135 |
| FC GAMMA RECEPTOR SIGNALING PATHWAY | 66 | -0.39159 | -1.42708 | 0.035629 | 0.433357 |
| NEGATIVE REGULATION OF ALPHA BETA T CELL ACTIVATION | 17 | -0.5231 | -1.42443 | 0.065789 | 0.438144 |
| POSITIVE REGULATION OF LEUKOCYTE DIFFERENTIATION | 115 | -0.34994 | -1.42432 | 0.01355 | 0.436112 |
| VITAMIN TRANSPORT | 27 | -0.45926 | -1.42358 | 0.056604 | 0.435895 |
| LYMPHOCYTE ACTIVATION INVOLVED IN IMMUNE RESPONSE | 72 | -0.38556 | -1.42259 | 0.029412 | 0.436459 |
| RESPONSE TO MANGANESE ION | 17 | -0.52014 | -1.42177 | 0.069351 | 0.436516 |
| BONE DEVELOPMENT | 131 | -0.34565 | -1.42137 | 0.010152 | 0.435591 |
| HEXOSE METABOLIC PROCESS | 128 | -0.34335 | -1.42115 | 0.007614 | 0.433981 |
| NEGATIVE REGULATION OF INTERLEUKIN 10 PRODUCTION | 15 | -0.55699 | -1.42003 | 0.086681 | 0.434789 |
| NEGATIVE REGULATION OF OSSIFICATION | 61 | -0.39051 | -1.4196 | 0.024752 | 0.434043 |
| REGULATION OF HYDROGEN PEROXIDE INDUCED CELL DEATH | 18 | -0.51746 | -1.41706 | 0.086364 | 0.43886 |
| CIRCADIAN REGULATION OF GENE EXPRESSION | 49 | -0.41198 | -1.41626 | 0.04662 | 0.439094 |
| DORSAL SPINAL CORD DEVELOPMENT | 18 | -0.50651 | -1.41617 | 0.08945 | 0.437158 |
| RNA STABILIZATION | 27 | -0.45769 | -1.4159 | 0.059701 | 0.435787 |
| MESODERM MORPHOGENESIS | 56 | -0.39863 | -1.41557 | 0.024814 | 0.434542 |
| RESPONSE TO LEPTIN | 18 | -0.52318 | -1.41428 | 0.06823 | 0.436123 |
| NUCLEAR ENVELOPE ORGANIZATION | 64 | -0.38621 | -1.41211 | 0.051095 | 0.440132 |
| NEGATIVE REGULATION OF CELL CELL ADHESION | 109 | -0.35018 | -1.41084 | 0.00995 | 0.441322 |
| REGULATION OF HAIR CYCLE | 21 | -0.49536 | -1.41065 | 0.064965 | 0.439773 |
| VASCULAR ENDOTHELIAL GROWTH FACTOR RECEPTOR SIGNALING PATHWAY | 64 | -0.38735 | -1.40968 | 0.023529 | 0.440513 |
| POSITIVE REGULATION OF LEUKOCYTE DEGRANULATION | 18 | -0.52078 | -1.40935 | 0.075431 | 0.439389 |
| REGULATION OF CYCLIN DEPENDENT PROTEIN KINASE ACTIVITY | 77 | -0.37564 | -1.40859 | 0.035629 | 0.439434 |
| POSITIVE REGULATION OF NUCLEOSIDE METABOLIC PROCESS | 22 | -0.48104 | -1.40856 | 0.076923 | 0.437462 |
| POSITIVE REGULATION OF IMMUNE EFFECTOR PROCESS | 132 | -0.34137 | -1.40809 | 0.010101 | 0.436826 |
| REGULATION OF MAP KINASE ACTIVITY | 274 | -0.31107 | -1.4069 | 0 | 0.437895 |
| REGULATION OF RECEPTOR BIOSYNTHETIC PROCESS | 17 | -0.5134 | -1.40507 | 0.075055 | 0.441113 |
| CELLULAR RESPONSE TO GROWTH HORMONE STIMULUS | 18 | -0.50654 | -1.40467 | 0.092342 | 0.440241 |
| POSITIVE REGULATION OF ALPHA BETA T CELL DIFFERENTIATION | 34 | -0.4428 | -1.4034 | 0.076923 | 0.441683 |
| POSITIVE REGULATION OF PHAGOCYTOSIS | 36 | -0.43412 | -1.40332 | 0.056948 | 0.439894 |
| RESPONSE TO CORTICOSTERONE | 22 | -0.48527 | -1.4029 | 0.093126 | 0.439147 |
| PURINERGIC NUCLEOTIDE RECEPTOR SIGNALING PATHWAY | 20 | -0.50456 | -1.40188 | 0.081081 | 0.439854 |
| CAMP MEDIATED SIGNALING | 31 | -0.44648 | -1.40019 | 0.069767 | 0.442435 |
| CELLULAR RESPONSE TO HORMONE STIMULUS | 477 | -0.29444 | -1.40015 | 0 | 0.440561 |
| REGULATION OF SIGNAL TRANSDUCTION BY P53 CLASS MEDIATOR | 130 | -0.33783 | -1.39915 | 0.020305 | 0.441532 |
| REGULATION OF TELOMERASE ACTIVITY | 36 | -0.42775 | -1.39872 | 0.077093 | 0.440773 |
| POSITIVE REGULATION OF CYTOKINE BIOSYNTHETIC PROCESS | 49 | -0.39422 | -1.39807 | 0.051724 | 0.440416 |
| FORMATION OF PRIMARY GERM LAYER | 95 | -0.35947 | -1.39782 | 0.023316 | 0.439244 |
| ADAPTIVE IMMUNE RESPONSE | 190 | -0.32119 | -1.39737 | 0.010309 | 0.438562 |
| REGULATION OF TRIGLYCERIDE METABOLIC PROCESS | 28 | -0.46198 | -1.39679 | 0.072261 | 0.43833 |
| MONOVALENT INORGANIC ANION HOMEOSTASIS | 18 | -0.49873 | -1.3956 | 0.077419 | 0.43978 |
| MODULATION OF TRANSCRIPTION IN OTHER ORGANISM INVOLVED IN SYMBIOTIC INTERACTION | 18 | -0.50361 | -1.39476 | 0.100418 | 0.440145 |
| BRANCHING INVOLVED IN SALIVARY GLAND MORPHOGENESIS | 15 | -0.52715 | -1.39344 | 0.112033 | 0.442245 |
| RESPONSE TO FUNGUS | 37 | -0.43127 | -1.39205 | 0.072368 | 0.444137 |
| REGULATION OF NITRIC OXIDE BIOSYNTHETIC PROCESS | 47 | -0.41008 | -1.39036 | 0.044917 | 0.44699 |
| REGULATION OF REACTIVE OXYGEN SPECIES METABOLIC PROCESS | 134 | -0.33717 | -1.38853 | 0.018373 | 0.450397 |
| MESODERMAL CELL DIFFERENTIATION | 20 | -0.4796 | -1.38818 | 0.103139 | 0.449374 |
| PROTEIN ACTIVATION CASCADE | 54 | -0.38309 | -1.38808 | 0.055427 | 0.447783 |
| SYNCYTIUM FORMATION | 24 | -0.46451 | -1.38687 | 0.08547 | 0.449161 |
| REGULATION OF BONE RESORPTION | 30 | -0.43415 | -1.38687 | 0.081858 | 0.44729 |
| MONOSACCHARIDE METABOLIC PROCESS | 157 | -0.32606 | -1.38644 | 0.015789 | 0.446663 |
| POSITIVE REGULATION OF LIPID TRANSPORT | 48 | -0.3962 | -1.38576 | 0.065882 | 0.446627 |
| HYALURONAN METABOLIC PROCESS | 27 | -0.44065 | -1.38551 | 0.098712 | 0.445328 |
| NEGATIVE REGULATION OF ERK1 AND ERK2 CASCADE | 45 | -0.40915 | -1.38548 | 0.05679 | 0.443552 |
| DEFENSE RESPONSE TO GRAM POSITIVE BACTERIUM | 48 | -0.39594 | -1.38107 | 0.059497 | 0.45409 |
| RESPONSE TO BACTERIUM | 396 | -0.29589 | -1.38022 | 0.006061 | 0.454489 |
| NEGATIVE REGULATION OF SYNAPTIC TRANSMISSION | 54 | -0.39048 | -1.37974 | 0.052995 | 0.454014 |
| NEGATIVE REGULATION OF MITOTIC NUCLEAR DIVISION | 25 | -0.46722 | -1.37961 | 0.07489 | 0.452565 |
| POSITIVE REGULATION OF SYNAPTIC TRANSMISSION GLUTAMATERGIC | 15 | -0.51464 | -1.37895 | 0.092213 | 0.452591 |
| CELLULAR RESPONSE TO FATTY ACID | 47 | -0.39869 | -1.37797 | 0.047059 | 0.453383 |
| EMBRYONIC CAMERA TYPE EYE MORPHOGENESIS | 22 | -0.4802 | -1.37706 | 0.078161 | 0.454196 |
| ALPHA BETA T CELL ACTIVATION | 46 | -0.40198 | -1.37552 | 0.049763 | 0.4567 |
| PHOSPHATIDYLINOSITOL 3 KINASE SIGNALING | 22 | -0.47891 | -1.37547 | 0.097403 | 0.455026 |
| KERATAN SULFATE METABOLIC PROCESS | 31 | -0.43668 | -1.37461 | 0.090069 | 0.455747 |
| T CELL ACTIVATION INVOLVED IN IMMUNE RESPONSE | 38 | -0.41649 | -1.37418 | 0.07243 | 0.455125 |
| ENDOCARDIAL CUSHION DEVELOPMENT | 26 | -0.45253 | -1.37358 | 0.08742 | 0.45487 |
| MULTI MULTICELLULAR ORGANISM PROCESS | 184 | -0.31687 | -1.37314 | 0.015113 | 0.454274 |
| T CELL DIFFERENTIATION INVOLVED IN IMMUNE RESPONSE | 24 | -0.46145 | -1.3722 | 0.065611 | 0.455013 |
| POSITIVE REGULATION OF LEUKOCYTE PROLIFERATION | 119 | -0.33784 | -1.37098 | 0.018913 | 0.456744 |
| LEUKOCYTE ACTIVATION | 336 | -0.29594 | -1.37045 | 0.002849 | 0.456659 |
| INFLAMMATORY RESPONSE | 359 | -0.29485 | -1.36965 | 0 | 0.457009 |
| NEGATIVE REGULATION OF TOLL LIKE RECEPTOR SIGNALING PATHWAY | 22 | -0.46983 | -1.36914 | 0.102397 | 0.456664 |
| REGULATION OF PHAGOCYTOSIS | 55 | -0.39536 | -1.36888 | 0.066975 | 0.455641 |
| REGULATION OF GLUCOSE IMPORT IN RESPONSE TO INSULIN STIMULUS | 16 | -0.51566 | -1.36871 | 0.114865 | 0.454389 |
| REGULATION OF HEMATOPOIETIC PROGENITOR CELL DIFFERENTIATION | 22 | -0.47955 | -1.36733 | 0.092473 | 0.456501 |
| PURINERGIC RECEPTOR SIGNALING PATHWAY | 26 | -0.44718 | -1.36631 | 0.08747 | 0.457775 |
| RESPONSE TO GAMMA RADIATION | 44 | -0.39801 | -1.36608 | 0.082725 | 0.456754 |
| REGULATION OF INTERLEUKIN 10 PRODUCTION | 39 | -0.42571 | -1.36501 | 0.082547 | 0.458045 |
| CELL ACTIVATION | 465 | -0.28622 | -1.36368 | 0 | 0.460023 |
| DNA TEMPLATED TRANSCRIPTION INITIATION | 156 | -0.32083 | -1.36354 | 0.013089 | 0.458674 |
| POSITIVE REGULATION OF ACTIVATED T CELL PROLIFERATION | 22 | -0.46908 | -1.36354 | 0.100427 | 0.456982 |
| REGULATION OF LEUKOCYTE DEGRANULATION | 36 | -0.40894 | -1.362 | 0.06772 | 0.459482 |
| REGULATION OF INTERLEUKIN 13 PRODUCTION | 15 | -0.51215 | -1.36199 | 0.110609 | 0.457824 |
| RESPONSE TO DIETARY EXCESS | 20 | -0.48537 | -1.36147 | 0.12 | 0.457559 |
| REGULATION OF CELL ACTIVATION | 396 | -0.28917 | -1.36103 | 0.003195 | 0.45709 |
| I KAPPAB KINASE NF KAPPAB SIGNALING | 56 | -0.38721 | -1.36101 | 0.056235 | 0.455511 |
| SPINDLE CHECKPOINT | 21 | -0.47088 | -1.36058 | 0.111857 | 0.455046 |
| EMBRYONIC DIGESTIVE TRACT DEVELOPMENT | 27 | -0.4418 | -1.35907 | 0.077295 | 0.457686 |
| NEGATIVE REGULATION OF CELL CYCLE | 346 | -0.29262 | -1.35849 | 0.003205 | 0.457464 |
| ESTABLISHMENT OF MITOTIC SPINDLE ORIENTATION | 20 | -0.48655 | -1.35707 | 0.104034 | 0.45982 |
| PEPTIDE HORMONE PROCESSING | 25 | -0.45516 | -1.35504 | 0.100687 | 0.463915 |
| REGULATION OF VASCULAR ENDOTHELIAL GROWTH FACTOR PRODUCTION | 28 | -0.44542 | -1.35331 | 0.082609 | 0.466883 |
| POSITIVE REGULATION OF CYTOKINE PRODUCTION | 306 | -0.2957 | -1.35302 | 0.005917 | 0.465998 |
| ENDOCARDIAL CUSHION MORPHOGENESIS | 17 | -0.50373 | -1.35268 | 0.106522 | 0.465263 |
| REGULATION OF CARDIAC MUSCLE CELL DIFFERENTIATION | 18 | -0.49017 | -1.35212 | 0.122807 | 0.465001 |
| HEMOSTASIS | 248 | -0.30291 | -1.35211 | 0.014663 | 0.463408 |
| MUCOPOLYSACCHARIDE METABOLIC PROCESS | 95 | -0.34597 | -1.35188 | 0.026316 | 0.462444 |
| OSTEOBLAST DIFFERENTIATION | 113 | -0.33546 | -1.35188 | 0.035533 | 0.460843 |
| POSITIVE REGULATION OF HEMOPOIESIS | 143 | -0.32627 | -1.35186 | 0.025575 | 0.459276 |
| REGULATION OF MUSCLE HYPERTROPHY | 36 | -0.41948 | -1.3517 | 0.088167 | 0.458088 |
| REGULATION OF DNA BIOSYNTHETIC PROCESS | 80 | -0.35175 | -1.35056 | 0.046838 | 0.459692 |
| CELLULAR RESPONSE TO INORGANIC SUBSTANCE | 133 | -0.32784 | -1.35042 | 0.028497 | 0.458532 |
| NECROTIC CELL DEATH | 24 | -0.44356 | -1.35006 | 0.098214 | 0.457984 |
