# Supplementary material for: Transcriptomic changes triggered by ouabain in rat cerebellum granule cells: Role of α3- and α1-Na+,K+-ATPase-mediated signaling
Source: PLoS One. 2019 Sep 26;14(9):e0222767. doi: 10.1371/journal.pone.0222767 (PMC6762055; doi:10.1371/journal.pone.0222767)
Supplement: S10 Table — (DOCX) [file pone.0222767.s022.docx]

**Table S10. Downregulated gene sets (GeneOntology – Biological Process) in 100nM ouabain-treated granular neurons at NES > 1.35.**

| **NAME** | **SIZE** | **ES** | **NES** | **NOM p-val** | **FDR q-val** |
| --- | --- | --- | --- | --- | --- |
| SYNAPSIS | 29 | 0.601897 | 1.78506 | 0 | 1 |
| CHROMOSOME ORGANIZATION INVOLVED IN MEIOTIC CELL CYCLE | 41 | 0.555237 | 1.779609 | 0.003515 | 1 |
| ADRENERGIC RECEPTOR SIGNALING PATHWAY | 19 | 0.66191 | 1.754688 | 0.001828 | 1 |
| MALE MEIOSIS | 31 | 0.573011 | 1.751349 | 0.003591 | 0.788833 |
| MEIOTIC CHROMOSOME SEGREGATION | 46 | 0.526363 | 1.747672 | 0.010676 | 0.660602 |
| DETECTION OF VISIBLE LIGHT | 35 | 0.557975 | 1.740465 | 0.001795 | 0.599958 |
| REGULATION OF RECEPTOR BINDING | 16 | 0.664977 | 1.73131 | 0.008945 | 0.573061 |
| HOMOLOGOUS CHROMOSOME SEGREGATION | 35 | 0.544431 | 1.720316 | 0.001761 | 0.565634 |
| FERTILIZATION | 116 | 0.446187 | 1.719202 | 0 | 0.509179 |
| DETECTION OF LIGHT STIMULUS | 49 | 0.511505 | 1.707221 | 0.001789 | 0.522929 |
| PHOTOTRANSDUCTION VISIBLE LIGHT | 17 | 0.650048 | 1.686091 | 0.019964 | 0.597973 |
| BILE ACID METABOLIC PROCESS | 31 | 0.546047 | 1.677484 | 0.012433 | 0.601043 |
| SODIUM ION TRANSMEMBRANE TRANSPORT | 79 | 0.464699 | 1.669343 | 0 | 0.602586 |
| SARCOMERE ORGANIZATION | 21 | 0.601216 | 1.668939 | 0.013986 | 0.561884 |
| SPERM MOTILITY | 43 | 0.507125 | 1.664065 | 0.003676 | 0.550136 |
| SENSORY PERCEPTION OF CHEMICAL STIMULUS | 326 | 0.385359 | 1.654027 | 0 | 0.568701 |
| REGULATION OF PROTEIN KINASE A SIGNALING | 16 | 0.638618 | 1.653147 | 0.014625 | 0.540511 |
| MEIOTIC CELL CYCLE PROCESS | 113 | 0.430408 | 1.639176 | 0.003328 | 0.586767 |
| DNA METHYLATION OR DEMETHYLATION | 42 | 0.50322 | 1.637377 | 0.010435 | 0.565601 |
| DNA MODIFICATION | 58 | 0.473467 | 1.629065 | 0.007067 | 0.582433 |
| CELLULAR MODIFIED AMINO ACID BIOSYNTHETIC PROCESS | 43 | 0.499461 | 1.624334 | 0.001828 | 0.578623 |
| PEPTIDYL ARGININE MODIFICATION | 15 | 0.634924 | 1.618916 | 0.014815 | 0.58086 |
| PHOTOTRANSDUCTION | 37 | 0.513672 | 1.612148 | 0.017857 | 0.591776 |
| PROTEIN ADP RIBOSYLATION | 16 | 0.615746 | 1.610382 | 0.022901 | 0.575645 |
| REGULATION OF TOR SIGNALING | 59 | 0.465317 | 1.609612 | 0.005245 | 0.556865 |
| MYOFIBRIL ASSEMBLY | 39 | 0.508238 | 1.607985 | 0.013865 | 0.543379 |
| ORGANIC HYDROXY COMPOUND TRANSPORT | 134 | 0.406385 | 1.607126 | 0 | 0.527291 |
| PEPTIDE CROSS LINKING | 22 | 0.5758 | 1.606003 | 0.014286 | 0.513522 |
| NEGATIVE REGULATION OF BLOOD PRESSURE | 43 | 0.488456 | 1.604015 | 0.0067 | 0.504836 |
| MEIOTIC CELL CYCLE | 139 | 0.401707 | 1.601392 | 0.003396 | 0.500177 |
| POSITIVE REGULATION OF TOR SIGNALING | 24 | 0.560778 | 1.594503 | 0.027675 | 0.515862 |
| DNA ALKYLATION | 34 | 0.515139 | 1.587881 | 0.027397 | 0.530431 |
| CHLORIDE TRANSPORT | 84 | 0.431329 | 1.587532 | 0.006944 | 0.515852 |
| ACID SECRETION | 61 | 0.453206 | 1.587341 | 0.005445 | 0.501777 |
| POSITIVE REGULATION OF RELEASE OF SEQUESTERED CALCIUM ION INTO CYTOSOL | 32 | 0.509694 | 1.584551 | 0.02381 | 0.500325 |
| REGULATION OF RHODOPSIN MEDIATED SIGNALING PATHWAY | 22 | 0.564689 | 1.582575 | 0.020446 | 0.494566 |
| LIPID DIGESTION | 18 | 0.601232 | 1.581045 | 0.030521 | 0.487945 |
| REGULATION OF CIRCADIAN SLEEP WAKE CYCLE | 24 | 0.547318 | 1.577794 | 0.03169 | 0.488889 |
| DIGESTION | 102 | 0.413139 | 1.56841 | 0.003436 | 0.51492 |
| MEMBRANE DEPOLARIZATION DURING ACTION POTENTIAL | 34 | 0.510714 | 1.561766 | 0.024955 | 0.530769 |
| POSITIVE REGULATION OF MULTICELLULAR ORGANISM GROWTH | 29 | 0.511952 | 1.560331 | 0.020677 | 0.523449 |
| REGULATION OF CALCIUM ION DEPENDENT EXOCYTOSIS | 73 | 0.433199 | 1.555594 | 0.011745 | 0.532305 |
| DETECTION OF STIMULUS | 445 | 0.345008 | 1.542224 | 0 | 0.582583 |
| CELL AGGREGATION | 19 | 0.560364 | 1.535772 | 0.044326 | 0.599102 |
| QUINONE METABOLIC PROCESS | 25 | 0.528349 | 1.529847 | 0.027778 | 0.614331 |
| MEIOSIS I | 63 | 0.441866 | 1.529737 | 0.015101 | 0.601503 |
| PEPTIDYL CYSTEINE MODIFICATION | 17 | 0.573427 | 1.524337 | 0.035382 | 0.615384 |
| ADAPTATION OF SIGNALING PATHWAY | 20 | 0.550199 | 1.520694 | 0.046099 | 0.619907 |
| REGULATION OF ANDROGEN RECEPTOR SIGNALING PATHWAY | 15 | 0.597853 | 1.515023 | 0.047619 | 0.636481 |
| HISTONE DEUBIQUITINATION | 15 | 0.579278 | 1.509352 | 0.04562 | 0.652594 |
| NEGATIVE REGULATION OF INTERLEUKIN 6 PRODUCTION | 28 | 0.50186 | 1.501685 | 0.031858 | 0.678622 |
| SINGLE FERTILIZATION | 87 | 0.407187 | 1.499684 | 0.008278 | 0.675063 |
| WATER TRANSPORT | 18 | 0.55212 | 1.497741 | 0.050089 | 0.672518 |
| DIGESTIVE SYSTEM PROCESS | 51 | 0.441457 | 1.491736 | 0.018998 | 0.692039 |
| REGULATION OF G PROTEIN COUPLED RECEPTOR PROTEIN SIGNALING PATHWAY | 112 | 0.388679 | 1.487616 | 0.006289 | 0.702295 |
| POSITIVE REGULATION OF G PROTEIN COUPLED RECEPTOR PROTEIN SIGNALING PATHWAY | 21 | 0.529846 | 1.486852 | 0.049645 | 0.693582 |
| REGULATION OF SYNAPTIC VESICLE EXOCYTOSIS | 17 | 0.561655 | 1.484242 | 0.064695 | 0.695699 |
| INTESTINAL ABSORPTION | 22 | 0.534048 | 1.482027 | 0.051601 | 0.695662 |
| NEGATIVE REGULATION OF BLOOD VESSEL ENDOTHELIAL CELL MIGRATION | 19 | 0.552273 | 1.48049 | 0.051095 | 0.691846 |
| GLUTAMATE SECRETION | 26 | 0.509166 | 1.476463 | 0.040516 | 0.700878 |
| ADENYLATE CYCLASE ACTIVATING G PROTEIN COUPLED RECEPTOR SIGNALING PATHWAY | 63 | 0.424899 | 1.474982 | 0.022648 | 0.697714 |
| FAT SOLUBLE VITAMIN METABOLIC PROCESS | 30 | 0.489348 | 1.474153 | 0.042328 | 0.690586 |
| INORGANIC ANION TRANSPORT | 109 | 0.383676 | 1.470088 | 0.010152 | 0.700891 |
| NEURON NEURON SYNAPTIC TRANSMISSION | 51 | 0.437295 | 1.463329 | 0.018425 | 0.72523 |
| INTRASPECIES INTERACTION BETWEEN ORGANISMS | 41 | 0.456304 | 1.461062 | 0.046595 | 0.726323 |
| SODIUM ION TRANSPORT | 128 | 0.375325 | 1.455692 | 0.01 | 0.74447 |
| RESPONSE TO SALT STRESS | 17 | 0.555278 | 1.454789 | 0.061947 | 0.738206 |
| NEGATIVE REGULATION OF G PROTEIN COUPLED RECEPTOR PROTEIN SIGNALING PATHWAY | 38 | 0.455617 | 1.454738 | 0.049724 | 0.727673 |
| POSITIVE REGULATION OF CYTOKINESIS | 25 | 0.50551 | 1.450785 | 0.048825 | 0.737355 |
| MONOCARBOXYLIC ACID TRANSPORT | 107 | 0.379672 | 1.448758 | 0.010033 | 0.737216 |
| RECIPROCAL DNA RECOMBINATION | 29 | 0.492614 | 1.445074 | 0.049091 | 0.746979 |
| STEROL TRANSPORT | 42 | 0.443825 | 1.444347 | 0.029091 | 0.74029 |
| KERATINIZATION | 23 | 0.510682 | 1.440024 | 0.062857 | 0.753424 |
| NEGATIVE REGULATION OF PEPTIDYL TYROSINE PHOSPHORYLATION | 35 | 0.458348 | 1.438786 | 0.064338 | 0.750004 |
| PYRIDINE CONTAINING COMPOUND BIOSYNTHETIC PROCESS | 15 | 0.551068 | 1.422279 | 0.073724 | 0.833439 |
| DETECTION OF ABIOTIC STIMULUS | 94 | 0.383411 | 1.418915 | 0.027027 | 0.841902 |
| POTASSIUM ION TRANSPORT | 141 | 0.359488 | 1.41548 | 0.011327 | 0.850914 |
| CAMP METABOLIC PROCESS | 32 | 0.46672 | 1.414157 | 0.063291 | 0.848171 |
| AXONAL FASCICULATION | 20 | 0.512543 | 1.413179 | 0.10536 | 0.842832 |
| DENDRITE DEVELOPMENT | 72 | 0.3974 | 1.412049 | 0.022767 | 0.838788 |
| INORGANIC ION TRANSMEMBRANE TRANSPORT | 498 | 0.312395 | 1.411596 | 0.00277 | 0.831191 |
| STEROID CATABOLIC PROCESS | 20 | 0.516082 | 1.411453 | 0.074349 | 0.821811 |
| SPERM EGG RECOGNITION | 32 | 0.461439 | 1.411334 | 0.07635 | 0.812758 |
| CELL SURFACE RECEPTOR SIGNALING PATHWAY INVOLVED IN CELL CELL SIGNALING | 65 | 0.404229 | 1.4105 | 0.043261 | 0.807703 |
| GMP METABOLIC PROCESS | 16 | 0.539547 | 1.407868 | 0.094241 | 0.812828 |
| SYNAPTIC TRANSMISSION DOPAMINERGIC | 16 | 0.538345 | 1.40402 | 0.095841 | 0.825947 |
| CGMP METABOLIC PROCESS | 22 | 0.494039 | 1.402664 | 0.072727 | 0.824328 |
| SYNAPTONEMAL COMPLEX ORGANIZATION | 19 | 0.519383 | 1.402481 | 0.076786 | 0.815987 |
| REGULATION OF CELL FATE COMMITMENT | 23 | 0.500991 | 1.401239 | 0.068468 | 0.813813 |
| ANION TRANSMEMBRANE TRANSPORT | 217 | 0.336362 | 1.399556 | 0.007962 | 0.813836 |
| SENSORY PERCEPTION OF TASTE | 46 | 0.432546 | 1.398098 | 0.049618 | 0.813476 |
| PHOTORECEPTOR CELL DEVELOPMENT | 34 | 0.455826 | 1.396475 | 0.067138 | 0.813118 |
| CEREBELLAR CORTEX MORPHOGENESIS | 29 | 0.465581 | 1.396131 | 0.066176 | 0.806333 |
| MALE GENITALIA DEVELOPMENT | 21 | 0.503064 | 1.395461 | 0.086331 | 0.801701 |
| DENDRITIC SPINE ORGANIZATION | 17 | 0.530272 | 1.392158 | 0.073665 | 0.811198 |
| MALE GAMETE GENERATION | 361 | 0.314211 | 1.390275 | 0.002941 | 0.813501 |
| ACTOMYOSIN STRUCTURE ORGANIZATION | 63 | 0.402375 | 1.389196 | 0.051155 | 0.811323 |
| REGULATION OF NEUROTRANSMITTER TRANSPORT | 53 | 0.410707 | 1.389083 | 0.072391 | 0.803485 |
| POST ANAL TAIL MORPHOGENESIS | 16 | 0.549277 | 1.38706 | 0.091912 | 0.806728 |
| REGULATION OF RESPONSE TO FOOD | 17 | 0.527435 | 1.385492 | 0.101145 | 0.807191 |
| RECEPTOR INTERNALIZATION | 44 | 0.428942 | 1.381895 | 0.053004 | 0.818835 |
| NEGATIVE REGULATION OF PROTEIN POLYMERIZATION | 48 | 0.407502 | 1.37898 | 0.063973 | 0.82711 |
| RESPONSE TO CADMIUM ION | 33 | 0.447339 | 1.375152 | 0.084229 | 0.840333 |
| CELL DIFFERENTIATION IN HINDBRAIN | 21 | 0.487803 | 1.374236 | 0.092559 | 0.837139 |
| LIPID CATABOLIC PROCESS | 210 | 0.326045 | 1.369334 | 0.012882 | 0.856984 |
| CEREBELLAR CORTEX DEVELOPMENT | 43 | 0.424713 | 1.369211 | 0.063545 | 0.849579 |
| NEGATIVE REGULATION OF ACTIN FILAMENT POLYMERIZATION | 38 | 0.429025 | 1.367112 | 0.063604 | 0.853765 |
| DNA CATABOLIC PROCESS | 22 | 0.486415 | 1.362578 | 0.083333 | 0.871737 |
| FATTY ACID BETA OXIDATION USING ACYL COA DEHYDROGENASE | 15 | 0.52422 | 1.360476 | 0.114786 | 0.875786 |
| CEREBELLAR PURKINJE CELL LAYER DEVELOPMENT | 21 | 0.493064 | 1.360139 | 0.090744 | 0.869747 |
| POSITIVE REGULATION OF CALCIUM ION TRANSPORT INTO CYTOSOL | 45 | 0.410848 | 1.359458 | 0.074783 | 0.865546 |
| AROMATIC AMINO ACID FAMILY METABOLIC PROCESS | 25 | 0.468106 | 1.359311 | 0.099828 | 0.85861 |
| DETECTION OF CHEMICAL STIMULUS INVOLVED IN SENSORY PERCEPTION OF TASTE | 29 | 0.456259 | 1.351475 | 0.082734 | 0.895309 |
| CYCLIC NUCLEOTIDE METABOLIC PROCESS | 53 | 0.401919 | 1.35122 | 0.061329 | 0.888983 |
