# Supplementary material for: Transcriptomic changes triggered by ouabain in rat cerebellum granule cells: Role of α3- and α1-Na+,K+-ATPase-mediated signaling
Source: PLoS One. 2019 Sep 26;14(9):e0222767. doi: 10.1371/journal.pone.0222767 (PMC6762055; doi:10.1371/journal.pone.0222767)
Supplement: S11 Table — (DOCX) [file pone.0222767.s023.docx]

**Table S11. Upregulated gene sets (GeneOntology – Molecular Function) in 100nM ouabain-treated granular neurons at NES < -1.35.**

| **NAME** | **SIZE** | **ES** | **NES** | **NOM p-val** | **FDR q-val** |
| --- | --- | --- | --- | --- | --- |
| TRANSCRIPTIONAL ACTIVATOR ACTIVITY RNA POLYMERASE II CORE PROMOTER PROXIMAL REGION SEQUENCE SPECIFIC BINDING | 202 | -0.42401 | -1.85809 | 0 | 0.312299 |
| INSULIN RECEPTOR BINDING | 28 | -0.59459 | -1.83521 | 0.00232 | 0.198826 |
| TRANSCRIPTION FACTOR ACTIVITY RNA POLYMERASE II CORE PROMOTER PROXIMAL REGION SEQUENCE SPECIFIC BINDING | 288 | -0.38586 | -1.75918 | 0 | 0.309005 |
| VITAMIN TRANSPORTER ACTIVITY | 21 | -0.61401 | -1.75729 | 0.006726 | 0.237031 |
| TRANSCRIPTION FACTOR ACTIVITY DIRECT LIGAND REGULATED SEQUENCE SPECIFIC DNA BINDING | 44 | -0.50401 | -1.70937 | 0.004902 | 0.293208 |
| TRANSCRIPTION COFACTOR BINDING | 23 | -0.58304 | -1.70078 | 0.019868 | 0.265918 |
| HISTONE ACETYLTRANSFERASE BINDING | 22 | -0.57901 | -1.67661 | 0.010965 | 0.282549 |
| TRANSCRIPTIONAL ACTIVATOR ACTIVITY RNA POLYMERASE II TRANSCRIPTION REGULATORY REGION SEQUENCE SPECIFIC BINDING | 280 | -0.35505 | -1.62139 | 0 | 0.400691 |
| HMG BOX DOMAIN BINDING | 16 | -0.59174 | -1.60376 | 0.029851 | 0.419 |
| 14 3 3 PROTEIN BINDING | 19 | -0.57103 | -1.59051 | 0.027907 | 0.42273 |
| STEROID HORMONE RECEPTOR ACTIVITY | 54 | -0.44055 | -1.58537 | 0.021898 | 0.399248 |
| KINASE INHIBITOR ACTIVITY | 79 | -0.40834 | -1.5746 | 0 | 0.394135 |
| SH3 DOMAIN BINDING | 101 | -0.39503 | -1.57265 | 0 | 0.368953 |
| GROWTH FACTOR BINDING | 107 | -0.37979 | -1.53173 | 0 | 0.468352 |
| CORE PROMOTER PROXIMAL REGION DNA BINDING | 278 | -0.34029 | -1.53143 | 0 | 0.43823 |
| ACTIVATING TRANSCRIPTION FACTOR BINDING | 49 | -0.42536 | -1.51553 | 0.020134 | 0.460178 |
| CYCLIN BINDING | 16 | -0.57432 | -1.50602 | 0.051836 | 0.463186 |
| TRANSCRIPTIONAL REPRESSOR ACTIVITY RNA POLYMERASE II CORE PROMOTER PROXIMAL REGION SEQUENCE SPECIFIC BINDING | 90 | -0.37701 | -1.48915 | 0.009975 | 0.490792 |
| CORE PROMOTER SEQUENCE SPECIFIC DNA BINDING | 81 | -0.38695 | -1.46522 | 0.012788 | 0.549536 |
| VIRUS RECEPTOR ACTIVITY | 58 | -0.39586 | -1.44109 | 0.030733 | 0.614719 |
| PROTEIN SERINE THREONINE TYROSINE KINASE ACTIVITY | 34 | -0.4464 | -1.43951 | 0.061674 | 0.590686 |
| CHROMATIN DNA BINDING | 63 | -0.39059 | -1.43521 | 0.029268 | 0.580926 |
| NEUROPEPTIDE BINDING | 21 | -0.49959 | -1.41502 | 0.074627 | 0.629672 |
| CORE PROMOTER BINDING | 128 | -0.34113 | -1.41206 | 0.012255 | 0.615505 |
| SERINE TYPE ENDOPEPTIDASE INHIBITOR ACTIVITY | 67 | -0.38058 | -1.39971 | 0.043902 | 0.636333 |
| PEPTIDASE INHIBITOR ACTIVITY | 122 | -0.34097 | -1.37587 | 0.029056 | 0.704853 |
| NON MEMBRANE SPANNING PROTEIN TYROSINE KINASE ACTIVITY | 41 | -0.40778 | -1.36374 | 0.063939 | 0.72976 |
| CHEMOKINE BINDING | 15 | -0.51313 | -1.35874 | 0.117914 | 0.725286 |
| PEPTIDASE REGULATOR ACTIVITY | 154 | -0.32263 | -1.35504 | 0.022843 | 0.714912 |
| FIBROBLAST GROWTH FACTOR BINDING | 19 | -0.4898 | -1.35254 | 0.098901 | 0.702197 |
