# Supplementary material for: Transcriptomic changes triggered by ouabain in rat cerebellum granule cells: Role of α3- and α1-Na+,K+-ATPase-mediated signaling
Source: PLoS One. 2019 Sep 26;14(9):e0222767. doi: 10.1371/journal.pone.0222767 (PMC6762055; doi:10.1371/journal.pone.0222767)
Supplement: S12 Table — (DOCX) [file pone.0222767.s024.docx]

**Table S12. Downregulated gene sets (GeneOntology – Molecular Function) in 100nM ouabain-treated granular neurons at NES > 1.35.**

| **NAME** | **SIZE** | **ES** | **NES** | **NOM p-val** | **FDR q-val** |
| --- | --- | --- | --- | --- | --- |
| TRANSFERASE ACTIVITY TRANSFERRING AMINO ACYL GROUPS | 15 | 0.717806 | 1.832937 | 0 | 0.333067 |
| SODIUM CHANNEL ACTIVITY | 33 | 0.581322 | 1.80828 | 0.006957 | 0.220898 |
| NAD ADP RIBOSYLTRANSFERASE ACTIVITY | 20 | 0.653594 | 1.782812 | 0.001802 | 0.201571 |
| CALCIUM ACTIVATED CATION CHANNEL ACTIVITY | 27 | 0.597782 | 1.781911 | 0.005545 | 0.152151 |
| ATPASE ACTIVITY COUPLED TO TRANSMEMBRANE MOVEMENT OF IONS PHOSPHORYLATIVE MECHANISM | 28 | 0.610405 | 1.77092 | 0.001757 | 0.139808 |
| ODORANT BINDING | 65 | 0.507432 | 1.769665 | 0 | 0.118474 |
| ION GATED CHANNEL ACTIVITY | 39 | 0.557356 | 1.763873 | 0 | 0.110319 |
| PHOSPHATIDYLCHOLINE BINDING | 17 | 0.666033 | 1.736169 | 0.005146 | 0.127972 |
| ACETYLGALACTOSAMINYLTRANSFERASE ACTIVITY | 26 | 0.590265 | 1.720589 | 0.007561 | 0.13632 |
| INTRACELLULAR LIGAND GATED ION CHANNEL ACTIVITY | 24 | 0.585515 | 1.676339 | 0.007313 | 0.198636 |
| QUATERNARY AMMONIUM GROUP BINDING | 42 | 0.521258 | 1.666159 | 0.006993 | 0.199919 |
| VOLTAGE GATED SODIUM CHANNEL ACTIVITY | 17 | 0.631126 | 1.649847 | 0.011132 | 0.213039 |
| AMMONIUM ION BINDING | 61 | 0.474485 | 1.641201 | 0 | 0.212378 |
| OLFACTORY RECEPTOR ACTIVITY | 254 | 0.382801 | 1.629552 | 0 | 0.219487 |
| OXYGEN BINDING | 32 | 0.530306 | 1.610061 | 0.018832 | 0.247611 |
| ANION CHANNEL ACTIVITY | 78 | 0.44351 | 1.604324 | 0 | 0.245978 |
| LIGAND GATED CHANNEL ACTIVITY | 123 | 0.408882 | 1.595323 | 0 | 0.249232 |
| CALCIUM ACTIVATED POTASSIUM CHANNEL ACTIVITY | 16 | 0.619547 | 1.59469 | 0.013035 | 0.236802 |
| CARBOXYLIC ESTER HYDROLASE ACTIVITY | 104 | 0.417009 | 1.593387 | 0.003384 | 0.227169 |
| SPECTRIN BINDING | 21 | 0.578876 | 1.578808 | 0.027624 | 0.244855 |
| EXTRACELLULAR LIGAND GATED ION CHANNEL ACTIVITY | 65 | 0.442115 | 1.556132 | 0.014493 | 0.285676 |
| GATED CHANNEL ACTIVITY | 281 | 0.361831 | 1.543546 | 0 | 0.302547 |
| MIRNA BINDING | 15 | 0.59321 | 1.535071 | 0.033392 | 0.309367 |
| ORGANIC HYDROXY COMPOUND TRANSMEMBRANE TRANSPORTER ACTIVITY | 49 | 0.445618 | 1.511353 | 0.025253 | 0.356031 |
| POLYPEPTIDE N ACETYLGALACTOSAMINYLTRANSFERASE ACTIVITY | 15 | 0.598032 | 1.511158 | 0.040367 | 0.342297 |
| TRIGLYCERIDE LIPASE ACTIVITY | 17 | 0.568744 | 1.5053 | 0.04936 | 0.346554 |
| LIPOPROTEIN PARTICLE RECEPTOR BINDING | 19 | 0.561719 | 1.502543 | 0.052632 | 0.340175 |
| MONOCARBOXYLIC ACID TRANSMEMBRANE TRANSPORTER ACTIVITY | 35 | 0.473419 | 1.497962 | 0.031088 | 0.339004 |
| PASSIVE TRANSMEMBRANE TRANSPORTER ACTIVITY | 397 | 0.333948 | 1.48978 | 0 | 0.349144 |
| EXCITATORY EXTRACELLULAR LIGAND GATED ION CHANNEL ACTIVITY | 47 | 0.444616 | 1.46822 | 0.04028 | 0.39854 |
| NEUROTRANSMITTER RECEPTOR ACTIVITY | 56 | 0.428568 | 1.465181 | 0.032534 | 0.394547 |
| G PROTEIN COUPLED AMINE RECEPTOR ACTIVITY | 43 | 0.451791 | 1.461602 | 0.035524 | 0.391426 |
| OXIDOREDUCTASE ACTIVITY ACTING ON PAIRED DONORS WITH INCORPORATION OR REDUCTION OF MOLECULAR OXYGEN REDUCED FLAVIN OR FLAVOPROTEIN AS ONE DONOR AND INCORPORATION OF ONE ATOM OF OXYGEN | 17 | 0.55133 | 1.453396 | 0.058052 | 0.402606 |
| LOW DENSITY LIPOPROTEIN PARTICLE RECEPTOR BINDING | 16 | 0.551253 | 1.423878 | 0.080734 | 0.482248 |
| CYCLIC NUCLEOTIDE BINDING | 28 | 0.477466 | 1.419546 | 0.06308 | 0.48207 |
| CALMODULIN BINDING | 150 | 0.350646 | 1.410162 | 0.009917 | 0.499668 |
| STRUCTURAL CONSTITUENT OF CYTOSKELETON | 77 | 0.390233 | 1.409522 | 0.030612 | 0.488391 |
| INORGANIC ANION TRANSMEMBRANE TRANSPORTER ACTIVITY | 108 | 0.36765 | 1.399268 | 0.021382 | 0.510379 |
| POLYSACCHARIDE BINDING | 15 | 0.558281 | 1.39765 | 0.089054 | 0.502891 |
| BICARBONATE TRANSMEMBRANE TRANSPORTER ACTIVITY | 18 | 0.524328 | 1.396561 | 0.087591 | 0.49383 |
| CATION TRANSPORTING ATPASE ACTIVITY | 47 | 0.420609 | 1.392774 | 0.05425 | 0.494346 |
| CAMP BINDING | 19 | 0.515038 | 1.39228 | 0.091228 | 0.484303 |
| POLYUBIQUITIN BINDING | 33 | 0.450202 | 1.38064 | 0.069324 | 0.510723 |
| ACID AMINO ACID LIGASE ACTIVITY | 15 | 0.540068 | 1.376263 | 0.114504 | 0.513887 |
| SODIUM ION TRANSMEMBRANE TRANSPORTER ACTIVITY | 122 | 0.351563 | 1.373302 | 0.03437 | 0.512456 |
| CYCLASE ACTIVITY | 21 | 0.499173 | 1.364864 | 0.104693 | 0.52911 |
| CYCLIC NUCLEOTIDE PHOSPHODIESTERASE ACTIVITY | 23 | 0.48195 | 1.362225 | 0.088339 | 0.527406 |
| SULFUR COMPOUND TRANSMEMBRANE TRANSPORTER ACTIVITY | 25 | 0.470981 | 1.354637 | 0.103321 | 0.54252 |
