# Supplementary material for: Transcriptomic changes triggered by ouabain in rat cerebellum granule cells: Role of α3- and α1-Na+,K+-ATPase-mediated signaling
Source: PLoS One. 2019 Sep 26;14(9):e0222767. doi: 10.1371/journal.pone.0222767 (PMC6762055; doi:10.1371/journal.pone.0222767)
Supplement: S13 Table — (DOCX) [file pone.0222767.s025.docx]

**Table S13. Upregulated gene sets (GeneOntology – Cellular Component) in 100nM ouabain-treated granular neurons at NES < -1.35.**

| **NAME** | **SIZE** | **ES** | **NES** | **NOM p-val** | **FDR q-val** |
| --- | --- | --- | --- | --- | --- |
| SPINDLE MIDZONE | 20 | -0.5512 | -1.54359 | 0.032609 | 1 |
| MAST CELL GRANULE | 18 | -0.54796 | -1.51842 | 0.051919 | 1 |
| COLLAGEN TRIMER | 64 | -0.40033 | -1.48592 | 0.028796 | 1 |
| CONDENSED CHROMOSOME CENTROMERIC REGION | 74 | -0.38911 | -1.48443 | 0.023641 | 1 |
| KINETOCHORE | 91 | -0.36153 | -1.41485 | 0.01995 | 1 |
| EXTERNAL SIDE OF PLASMA MEMBRANE | 178 | -0.32279 | -1.40541 | 0.012788 | 1 |
| PHAGOCYTIC CUP | 17 | -0.51311 | -1.40407 | 0.067538 | 1 |
| PROTON TRANSPORTING V TYPE ATPASE COMPLEX | 20 | -0.47844 | -1.38465 | 0.097872 | 1 |
