# Supplementary material for: Transcriptomic changes triggered by ouabain in rat cerebellum granule cells: Role of α3- and α1-Na+,K+-ATPase-mediated signaling
Source: PLoS One. 2019 Sep 26;14(9):e0222767. doi: 10.1371/journal.pone.0222767 (PMC6762055; doi:10.1371/journal.pone.0222767)
Supplement: S14 Table — (DOCX) [file pone.0222767.s026.docx]

**Table S14. Downregulated gene sets (GeneOntology – Cellular Component) in 100nM ouabain-treated granular neurons at NES > 1.35.**

| **NAME** | **SIZE** | **ES** | **NES** | **NOM p-val** | **FDR q-val** |
| --- | --- | --- | --- | --- | --- |
| ACROSOMAL VESICLE | 79 | 0.516458 | 1.883048 | 0 | 0.075062 |
| SPERM PART | 116 | 0.473134 | 1.808889 | 0 | 0.11708 |
| ATPASE COMPLEX | 23 | 0.624572 | 1.783406 | 0 | 0.105713 |
| CILIARY MEMBRANE | 66 | 0.493363 | 1.741397 | 0.003322 | 0.131289 |
| ACROSOMAL MEMBRANE | 18 | 0.628234 | 1.65613 | 0.010695 | 0.262146 |
| TRANSPORTER COMPLEX | 283 | 0.381837 | 1.654095 | 0 | 0.22246 |
| SYNAPTONEMAL COMPLEX | 28 | 0.549664 | 1.624497 | 0.013566 | 0.254463 |
| CATION TRANSPORTING ATPASE COMPLEX | 15 | 0.627047 | 1.610777 | 0.016514 | 0.25337 |
| CILIARY PART | 232 | 0.379161 | 1.607312 | 0 | 0.234786 |
| PRESYNAPTIC ACTIVE ZONE | 24 | 0.521862 | 1.508806 | 0.035714 | 0.493518 |
| CENTRIOLAR SATELLITE | 20 | 0.540269 | 1.493313 | 0.039076 | 0.509724 |
| CHLORIDE CHANNEL COMPLEX | 43 | 0.460846 | 1.490262 | 0.023609 | 0.477601 |
| INTERMEDIATE FILAMENT | 90 | 0.403844 | 1.48886 | 0.004934 | 0.445165 |
| SARCOPLASMIC RETICULUM MEMBRANE | 30 | 0.491586 | 1.479273 | 0.039474 | 0.446783 |
| SPERM FLAGELLUM | 45 | 0.453328 | 1.472928 | 0.026978 | 0.438738 |
| PRIMARY CILIUM | 163 | 0.36596 | 1.467026 | 0.001563 | 0.42912 |
| NONMOTILE PRIMARY CILIUM | 107 | 0.383104 | 1.459426 | 0.015306 | 0.426845 |
| CILIUM | 361 | 0.329521 | 1.454109 | 0 | 0.420262 |
| PSEUDOPODIUM | 17 | 0.539531 | 1.428878 | 0.074349 | 0.482485 |
| MOTILE CILIUM | 86 | 0.37953 | 1.400426 | 0.025729 | 0.562378 |
| SMALL SUBUNIT PROCESSOME | 28 | 0.46543 | 1.400407 | 0.06903 | 0.535598 |
| INTERMEDIATE FILAMENT CYTOSKELETON | 124 | 0.357717 | 1.387818 | 0.021776 | 0.556728 |
| NUCLEAR OUTER MEMBRANE | 19 | 0.50677 | 1.374554 | 0.092896 | 0.582908 |
| GABA RECEPTOR COMPLEX | 17 | 0.528219 | 1.373352 | 0.095331 | 0.563151 |
| POLYSOME | 35 | 0.446824 | 1.360911 | 0.098787 | 0.587873 |
| CONTRACTILE FIBER | 159 | 0.334546 | 1.354123 | 0.021922 | 0.592254 |
| PERIKARYON | 96 | 0.355368 | 1.35242 | 0.04698 | 0.576975 |
